# Design, synthesis, and in silico studies of quinoline-based-benzo[d]imidazole bearing different acetamide derivatives as potent α-glucosidase inhibitors

**DOI:** 10.1038/s41598-022-18455-7

**Published:** 2022-08-18

**Authors:** Milad Noori, Ali Davoodi, Aida Iraji, Navid Dastyafteh, Minoo Khalili, Mehdi Asadi, Maryam Mohammadi Khanaposhtani, Somayeh Mojtabavi, Mehdi Dianatpour, Mohammad Ali Faramarzi, Bagher Larijani, Massoud Amanlou, Mohammad Mahdavi

**Affiliations:** 1 Endocrinology and Metabolism Research Center, Endocrinology and Metabolism Clinical Sciences Institute, Tehran University of Medical Sciences, Tehran, Iran; 2 Department of Medicinal Chemistry, Faculty of Pharmacy, Tehran University of Medical Sciences, Tehran, Iran; 3 Stem Cells Technology Research Center, Shiraz University of Medical Sciences, Shiraz, Iran; 4 Central Research Laboratory, Shiraz University of Medical Sciences, Shiraz, Iran; 5 Cellular and Molecular Biology Research Center, Health Research Institute, Babol University of Medical Sciences, Babol, Iran; 6 Department of Pharmaceutical Biotechnology, Faculty of Pharmacy and Biotechnology Research Center, Tehran University of Medical Sciences, Tehran, Iran

**Keywords:** Enzymes, Chemical biology, Drug discovery, Medicinal chemistry, Drug discovery and development

## Abstract

In this study, 18 novel quinoline-based-benzo[d]imidazole derivatives were synthesized and screened for their α-glucosidase inhibitory potential. All compounds in the series except **9q** showed a significant α-glucosidase inhibition with IC_50_ values in the range of 3.2 ± 0.3–185.0 ± 0.3 µM, as compared to the standard drug acarbose (IC_50_ = 750.0 ± 5.0 µM). A kinetic study indicated that compound **9d** as the most potent derivative against α-glucosidase was a competitive type inhibitor. Furthermore, the molecular docking study revealed the effective binding interactions of **9d** with the active site of the α-glucosidase enzyme. The results indicate that the designed compounds have the potential to be further studied as new anti-diabetic agents.

## Introduction

Diabetes mellitus (DM) is a chronic metabolic disease characterized by hyperglycemia, with the disorder in carbohydrate, fat, and protein metabolism in the body^1^. DM is known as an important public health threat with around 450 million cases worldwide in 2019. This number is expected to rise to 700 million by 2045 worldwide confirming further action is required in this field^2,3^. Long-term DM can increase the risk of various health complications including blindness, renal failure, foot amputation, as well as cardiovascular, retinopathy, and renal diseases^4^. Type 2 diabetes mellitus (T2DM) with around 90% of all cases is categorized as a major sub-type of DM. It was considered that glycemic control could be effective prevention and treatment for T2DM^5–7^.

α-Glucosidase (EC 3.2.1.20) is a catalytic hydrolase enzyme present on the brush border of the small intestine which hydrolyzes oligosaccharides, trisaccharides, and disaccharides to glucose and other monosaccharides at their non-reducing ends^7–10^. The produced monosaccharides especially glucose enter the bloodstream, resulting in postprandial hyperglycemia thus causing diabetes^11–13^. Therefore, the inhibition of α-glucosidase might reduce carbohydrate digestion, delay glucose uptake, and consequently, decrease blood sugar levels^14,15^. The α-glucosidase enzyme can be inhibited by acarbose, voglibose, and miglitol with sub-optimal efficacy^16^. Also, long-term administration of mentioned inhibitor may cause several side effects, such as abdominal pain, diarrhea, and flatulence. As a result, a need of effective inhibitors to target α-glucosidase is highly needed^17–19^.

In the last few decades, different synthetic small molecules including benzo[d]imidazole^20^, isatin^21^ benzo[b]thiophene^22^ pyrimidine^23^, xanthone^24^, chromene^6^, azole^18,25^ against α-glucosidase attracted increasing attention.

Regarding promising anti-diabetic properties of quinolone heterocyclic scaffold and benzo[d]imidazole moiety, in this study, the novel series of quinoline-based-benzo[d]imidazole bearing different acetamide derivatives were synthesized, and evaluated for their inhibition potential against the α-glucosidase enzyme. Also, kinetic as well as molecular docking studies of the most potent compound were performed to evaluate their inhibition pattern against α-glucosidase.

## Results and discussion

### Design of quinoline-based-benzo[d]imidazole derivatives

**Figure 1:**
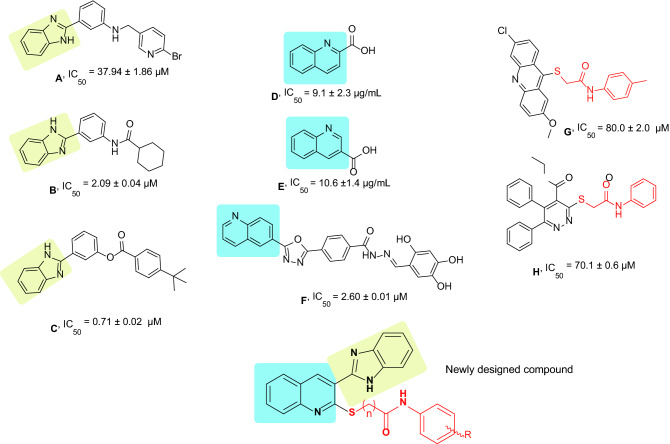
Schematic illustration of previously reported α-glucosidase inhibitors and newly designed compound.

During the last years, several non-sugar-based α-glucosidase inhibitors were identified. The random screening of the in-house library resulted in introducing compound **A** (Fig. 1) bearing benzo[d]imidazole moiety with good potency against α-glucosidase^20^. The follow-up structural optimization of **A** resulted in a series of novel 2-phenyl-1H-benzo[d]imidazole derivatives (compound **B** and **C**) with IC_50_ values in the range of 0.71 to > 100 µM compared to the acarbose as a positive control with an IC_50_ value of 258.53 ± 1.27 µM. Preliminary structure–activity relationships (SARs) study revealed that the benzo[d]imidazole core played key role in the inhibition of α-glucosidase activity^17^.

Also, recent studies demonstrated the α-glucosidase inhibitory activity of quinoline-containing compounds. The preliminary bioassay results revealed that compounds **D** and **E** (Fig. 1) had significant inhibitory potency compared to acarbose (IC_50_ = 66.5 ± 1.5 µg/mL)^26^. To further improve the α-glucosidase inhibitory activity of quinolone derivatives, the structural modification was carried out. These analogs exhibited inhibitory potential with IC_50_ values in the ranges between 2.60 and 102.12 μM (Compound **F,** Fig. 1)^27^.

Furthermore, it was reported that methyl-thioacetamide moiety (compounds **G** and **H**) can not only improve α-glucosidase inhibition through generating optimum structure to effectively participate within the active site but also provide a suitable site for derivatization^28–30^.

In this context, molecular hybridization as a powerful tool for drug designing was applied so that benzo[d]imidazole and quinoline as potent heterocyclic pharmacophores were conjugated to different acetamide derivatives. Novel designed compounds were synthesized and evaluated for their α-glucosidase inhibitory activities. Preliminary SAR studies were conducted. Further, kinetic study plus in silico assessments were performed to evaluate the binding of the active compound to the enzyme.

### Chemistry

**Figure 2:**
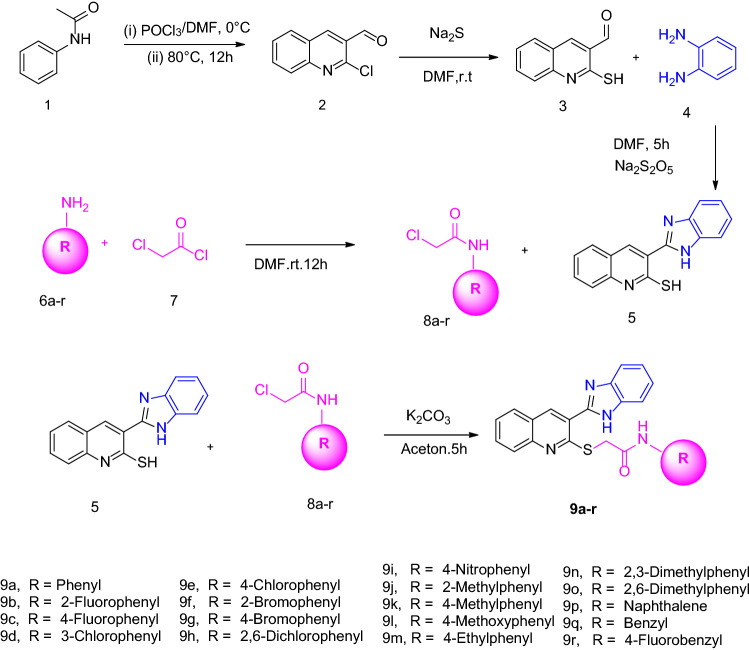
Synthesis of compounds **9a–r**.

The synthesis of compounds **9a–r** is schematically shown in Fig. 2. Briefly, phosphoryl chloride in *N,N*-dimethylformamide (DMF) was added dropwise to the cold *N*-phenylacetamide (**1**) under reflux conditions for 15 h to obtain 2-chloroquinoline-3-carbaldehyde (**2**)^31^. Compound **2** and sodium sulfide were then dissolved in DMF and stirred at room temperature for 2 h to achieve 3-formyl-2-mercaptoquinoline (**3**)^32^. Then, the reaction of O-phenylenediamines (**4**) and 3-formyl-2-mercaptoquinoline (**3**) in the presence of sodium metabisulfite in DMF at 150 °C for 2 h afforded the target compound **5**^33^. Synthesis of desired compounds **8a–r** was performed through the reaction of aniline derivatives (**6a–r**) with chloroacethylchloride (**7**) in DMF^34^. Finally, the reaction of compounds **8a–r** and compound **5** in acetone in presence of K_2_CO_3_ led to the formation of products **9a–r**^35^. The structure of all compounds was confirmed using NMR and IR spectroscopy as well as elemental analysis.

### Structure–activity relationship (SAR) exploration

The results of the α-glucosidase inhibitory assay are displayed in Table 1. In general, all compounds showed significant α-glucosidase inhibition with IC_50_ values in the range of 3.2 ± 0.3 to 185.0 ± 0.3 µM in comparison to acarbose with an IC_50_ value of 750.0 ± 5.0 µM. The exception come back to **9q** which showed IC_50_ > 750.

**Table 1:** α-Glucosidase inhibitory activity of compounds **9a–r**.


Compound	R	IC_50_ (μM)^a^	Concentrations of precipitation (µM)
**9a**	Phenyl	30.2 ± 0.4	≥ 200
**9b**	2-Fluorophenyl	61.3 ± 0.4	≥ 200
**9c**	4-Fluorophenyl	13.5 ± 0.6	≥ 200
**9d**	3-Chlorophenyl	3.2 ± 0.3	≥ 200
**9e**	4-Chlorophenyl	110.4 ± 0.2	≥ 200
**9f**	2-Bromophenyl	23.4 ± 0.2	≥ 200
**9g**	4-Bromophenyl	185.0 ± 0.3	≥ 200
**9h**	2,6-Dichlorophenyl	100.8 ± 0.1	≥ 200
**9i**	4-Nitrophenyl	19.7 ± 0.2	≥ 200
**9j**	2-Methylphenyl	16.5 ± 0.4	≥ 200
**9k**	4-Methylphenyl	12.3 ± 0.2	≥ 200
**9l**	4-Methoxyphenyl	5.7 ± 0.3	≥ 200
**9m**	4-Ethylphenyl	55.6 ± 0.2	≥ 200
**9n**	2,3-Dimethylphenyl	9.8 ± 0.5	≥ 200
**9o**	2,6-Dimethylphenyl	147.0 ± 0.2	≥ 200
**9p**	Naphthalene	17.7 ± 0.8	≥ 200
**9q**	Benzyl	750 <	≥ 200
**9r**	4-Fluorobenzyl	33.0 ± 0.1	≥ 200
**Acarbose**	–	750.0 ± 5.0	–

^a^Data represented in terms of mean ± SD.

As can be seen in Table 1, benzimidazole-thioquinoline structure bearing phenylacetamide exhibited good inhibitory activities against α-glucosidase (**9a**, IC_50_ = 30.2 ± 0.4 µM). The incorporation of a fluorine atom at the *ortho* position of phenylacetamide (**9b**) resulted in an around the twofold loss of potency compared to **9a.** Furthermore, changing the position from *ortho* to *para* in compound **9c** (IC_50_ = 13.5 ± 0.6 µM) resulted in the second potent derivative in the halogen-substituted set.

Impotently, the introduction of 3-chlorophenyl at R position, compounds **9d,** displayed a significant α-glucosidase inhibition (IC_50_ = 3.2 ± 0.3 µM) with around 250-fold improvement in the potency compared to the positive control, acarbose. Indeed, compound **9e** (R = 4-chlorophenyl, IC_50_ = 110.4 ± 0.2 µM) had inferior activity compared to **9d**. Replacement of chlorine substitution with bromine resulted in compounds **9f** and **9g**. Compound **9f** (R = 2-Bromophenyl) was another potent derivative in halogen-substituted set (IC_50_ = 23.4 ± 0.2 µM). However converting 2-Br substitution to 4-Br was not favorable (**9g**, IC_50_ = 185.0 ± 0.3 µM) compared to **9f.** Additionally, **9h** as the multi-substituted chlorine derivative with inferior activities compared with **9c,** still exhibited promising potency compared to acarbose.

Overall, the mono-electron withdrawing group (EWG) at *para* position had a destructive effect against α-glucosidase while *ortho* and *meta* position seems more favorable. The exception in this trend came back to **9c** bearing 4-fluorine. This could be due to the smaller size and better electronegativity compared to the rest of halogen derivatives.

The evaluations on **9j–m** as the mono electron-donating-substituted group (EDG) showed overall improvement in the potency so that **9l** (R = 4-methoxyphenyl) with an IC_50_ of 5.7 ± 0.3 μM was categorized as the top potent inhibitor in this group and second top potent entry among all derivatives followed by **9k** (R = 4-methyl phenyl) and **9j** (R = 2-methyl phenyl). Next, the assessment of compounds **9n** and **9o** were performed and, disappointingly, **9o** bearing symmetric multi-substituted moiety (R = 2,6-diCH_3_, IC_50_ = 147.0 µM) recorded the reduction in the activity compared to **9n**. However, **9o** derivative still demonstrated around eightfold improvement in the potency compared to acarbose with IC_50_ of 750.0 µM.

Precise assessments on the **9j–o** derivatives also indicated that the position of substitutions seems to have the most dominant role in the inhibition compared to the lipophilicity of moiety.

Ring substitution assessments were also performed in which phenyl (**9a**) was replaced with naphthyl (**9p**). An improvement in the activity showed that a bulk structure is more favorable.

Next, the investigation of SAR indicated that nitro (**9i**, IC_50_ = 19.7 ± 0.2 µM) and methoxy (**9l**, IC_50_ = 5.7 ± 0.3 µM) moieties were optimal substituents at the *para* position of phenylacetamide which improved the α-glucosidase inhibition. These results suggested that such substitution may probably enhance the ligand–protein interaction with the α-glucosidase active site.

**9q** and **9r** were also synthesized to evaluate the role of elongation of the linker between aryl substitutions and thioacteamide moiety. Compound **9q** with benzyl substitution exhibited dramatically reduction in the α-glucosidase inhibition compared to **9a** which exhibited the destructive effect of elongation of the linker in the unsubstituted derivatives. Also, there was a similar trend in the potency in **9r** bearing 4-fluorobenzyl compared to **9c** (R = 4-Fluorophenyl).

**Figure 3:**
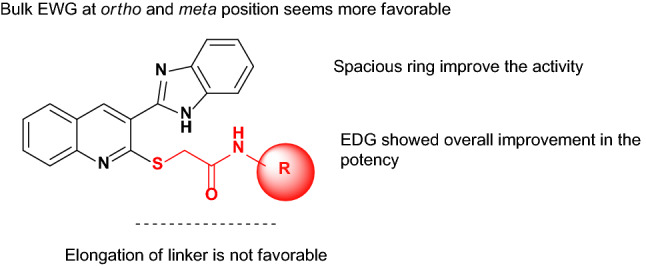
Summary of the SARs.

The summary of the SARs to improve α-glucosidase inhibitory activity was depicted in Fig. 3. Overall, it can be understood that the most potent derivative (**9d**) exhibited better inhibitory activity against a-glucosidase compared to lead compounds including **A** to **G** reported in Fig. 1 concerning their positive control.

### Enzyme kinetic studies

**Figure 4:**
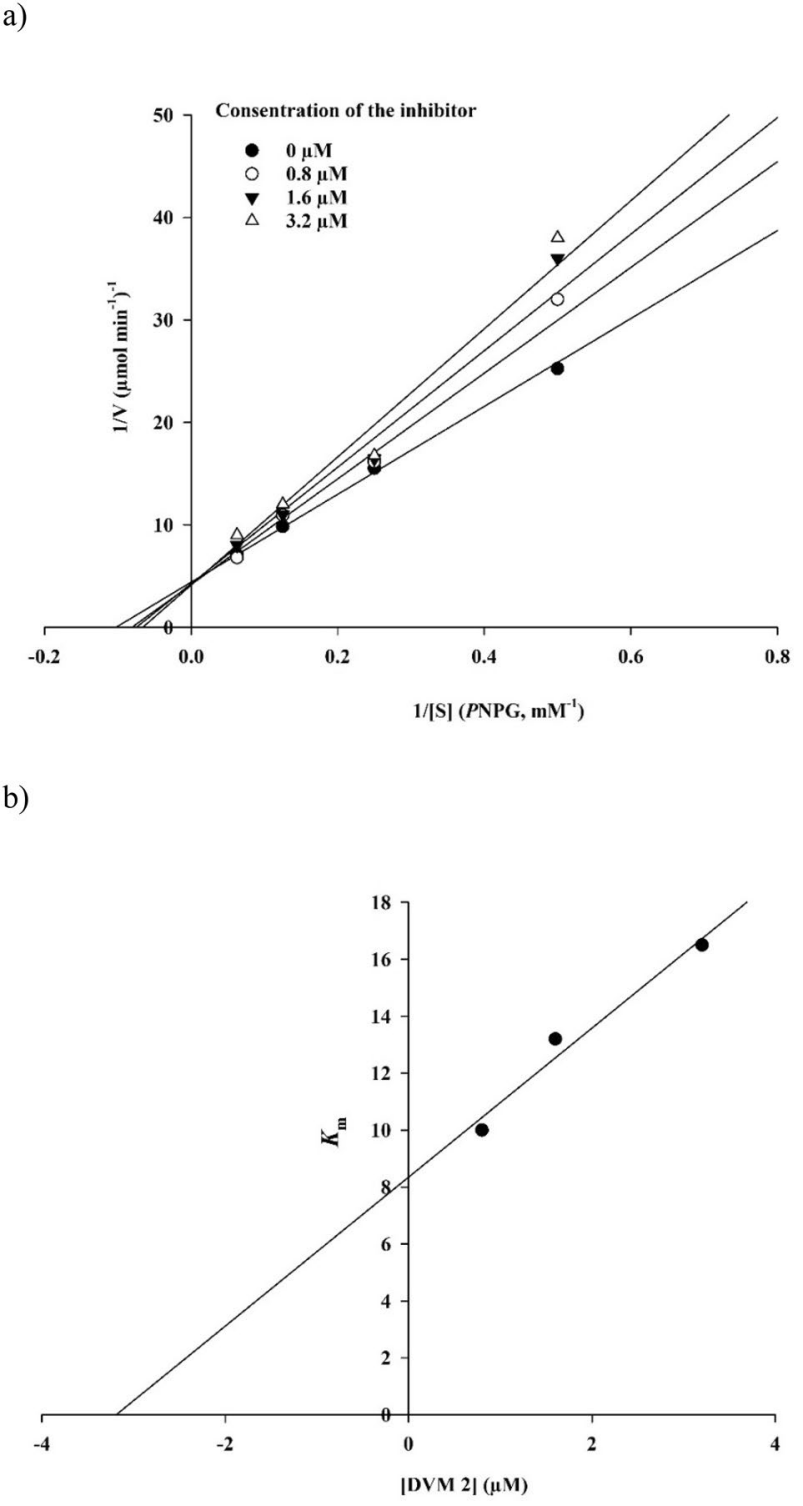
Kinetics of α-glucosidase inhibition by **9d**. (**a**) The Lineweaver–Burk plot in the absence and presence of different concentrations of **9d**; (**b**) the secondary plot between *K*_m_ and various concentrations of **9d**.

To gain insight into the mechanism of action of **9d** as the most potent α-glucosidase inhibitor, kinetic measurements were performed. According to Fig. 4a, the Lineweaver–Burk plot showed that the *K*_m_ gradually increased and *V*_*max*_ remained unchanged with increasing inhibitor concentration indicating a competitive inhibition. The results show **9d** bonded to the active site on the enzyme and compete with the substrate for binding to the active site. Furthermore, the plot of the *K*_m_ versus different concentrations of inhibitor gave an estimate of the inhibition constant, *K*_i_ of 3.2 µM (Fig. 4b).

### Docking analyses

To identify the accuracy and validation of docking procedures, the self-docking of acarbose (as a crystallographic ligand) was performed through induced fit docking of Schrödinger software. Alignment of the best pose of acarbose in the active site of α-glucosidase and crystallographic ligand recorded an RMSD value of 1.73 Å (RMSD should be less than 2 Å) which confirms the accuracy of docking. Next, the same docking procedure was repeated with all derivatives and their binding to α-glucosidase was analyzed. Results are summarized in Table 2.

**Table 2:** Docking scores and interactions of compounds against the α-glucosidase (PDB ID: 5NN8).

Compound	R	Glide score	Amino acid	Type of interaction
**9a**	Phenyl	− 6.43	Asp616	H-bound
			Leu678	H-bound
			Phe649	Pi–pi stacking
**9b**	2-Fluorophenyl	− 5.81	Asp616	H-bound
			Asp282	H-bound
**9c**	4-Fluorophenyl	− 6.59	Trp376	Pi–pi stacking
			Trp481	Pi–pi stacking
			Leu677	H-bound
			Asp616	H-bound
			Arg600	Pi–cation
			Asp518	Salt bridge
**9d**	3-Chlorophenyl	− 6.92	Arg600	Pi–cation
			Trp481	H-bound
			Asp616	H-bound
			Asp616	H-bound
			Leu677	Halogen bound
**9e**	4-Chlorophenyl	− 5.39	Arg600	Pi–cation
			Asp282	H-bound
			Trp481	H-bound
**9f**	2-Bromophenyl	− 6.14	Arg600	Pi–cation
			Asp282	H-bound
			Trp481	H-bound
**9g**	4-Bromophenyl	− 4.65	Asp282	H-bound
			Trp481	Pi–pi stacking
			Phe649	Pi–pi stacking
**9h**	2,6-Dichlorophenyl	− 4.99	Trp481	Pi–pi stacking
			Trp481	Pi–pi stacking
			Trp481	Pi–pi stacking
			Phe649	Pi–pi stacking
			Phe649	Pi–pi stacking
**9i**	4-Nitrophenyl	− 6.31	Asp616	H-bound
			Asp282	H-bound
			Arg281	Pi–cation
**9j**	2-Methylphenyl	− 6.13	Asp616	H-bound
			Asp282	H-bound
			Phe525	Pi–pi stacking
**9k**	4-Methylphenyl	− 6.72	Phe649	Pi–pi stacking
			Phe649	Pi–pi stacking
			Asp616	H-bound
			Ser676	H-bound
**9l**	4-Methoxyphenyl	− 6.33	Leu677	H-bound
			Asp616	H-bound
			Phe649	Pi–pi stacking
			Trp481	Pi–pi stacking
**9m**	4-Ethylphenyl	− 6.54	Asp282	H-bound
			Asp282	H-bound
			Trp481	H-bound
**9n**	2,3-Dimethylphenyl	− 6.900	Asp616	H-bound
			Trp481	H-bound
			Phe649	Pi–pi stacking
**9o**	2,6-Dimethylphenyl	− 5.53	Asp616	H-bound
			Asp282	H-bound
**9p**	Naphthalene	− 6.25	Asp616	H-bound
			Asp616	H-bound
			Trp481	H-bound
			Phe525	Pi–pi stacking
			Trp376	Pi–pi stacking
**9q**	Benzyl	− 4.30	Trp376	Pi–pi stacking
			Phe525	Pi–pi stacking
**9r**	4-Fluorobenzyl	− 5.90	Asp282	H-bound
			Asp282	H-bound
			Trp376	Pi–pi stacking
			Trp481	Pi–pi stacking
			Trp481	Pi–pi stacking
			Trp481	Pi–pi stacking
**Acarbose**	–	− 6.14	Asp616	H-bound
			Asp616	Salt bridge
			Asp518	H-bound
			Phe525	H-bound

The in silico studies showed the binding energy of acarbose was − 6.14 kcal/mol while the glide score value of **9a–r** ranges from − 4.65 to − 6.92 kcal/mol. As can be seen, the most potent derivative in in vitro assay was **9d** (IC_50_ = 3.2 ± 0.3) > **9l** (IC_50_ = 5.7 ± 0.3 µM) > **9n** (IC_50_ = 9.8 ± 0.5 µM) > **9k** (IC_50_ = 12.3 ± 0.2 µM) exhibited the best in silico results with glide score value of − 6.92, − 6.33, − 6.90 and − 6.72 kcal/mol, respectively. Assessments on lest potent derivatives, **9q** (IC_50_ > 750), **9g** (IC_50_ = 185.0 ± 0.3) and **9o** (IC_50_ = 147.0 ± 0.2) reveal low binding interaction with the targeted enzyme with binding energy of − 4.30, − 4.65 and − 4.99 value.

**Figure 5:**
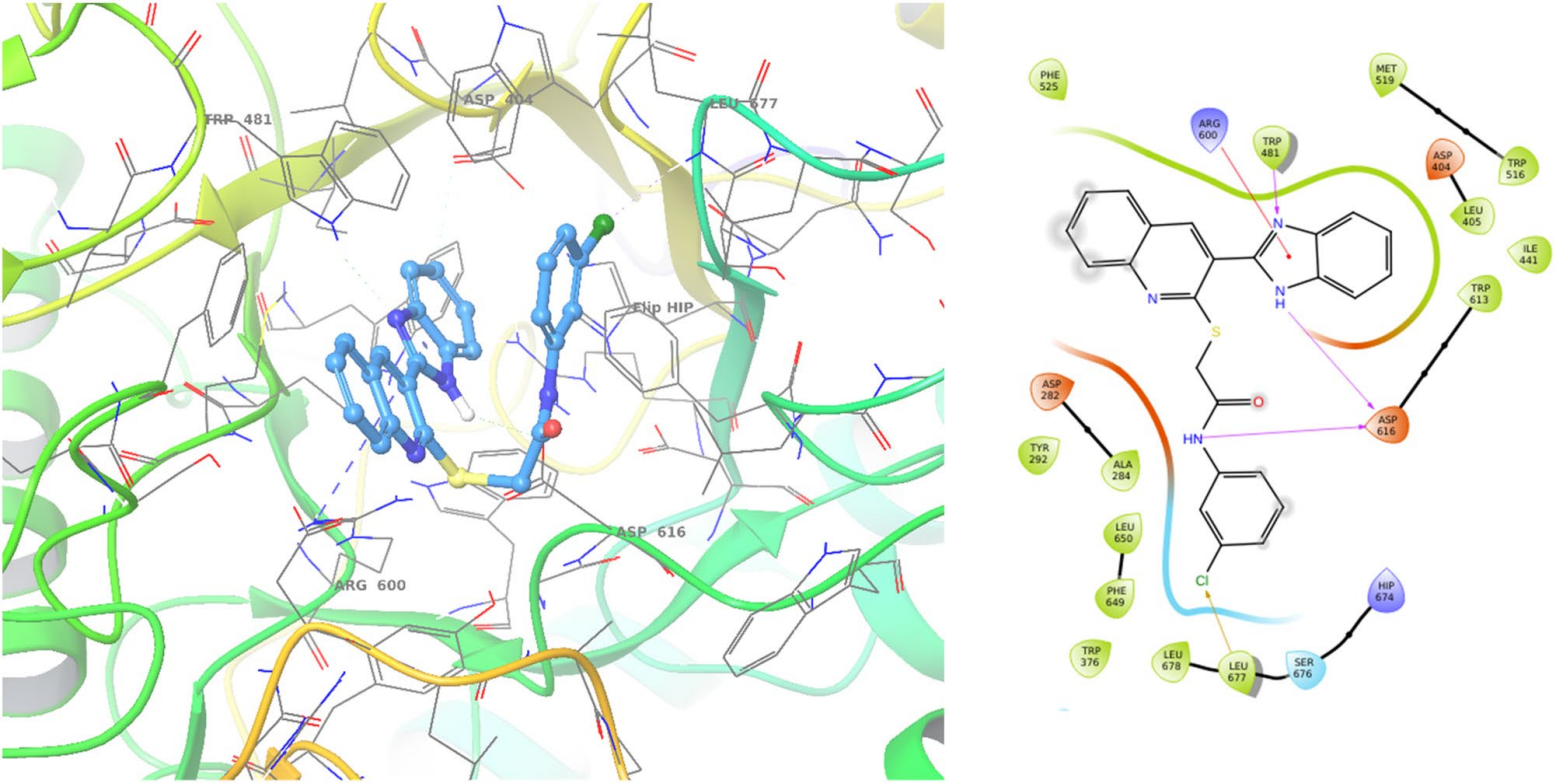
3D and 2D proposed binding modes of compounds **9d** (blue color) with α-glucosidase.

The docking results between α-glucosidase and compound **9d** was shown in Fig. 5. Compound **9d** was well inserted into the active site and recorded a Glide score of − 6.92. Compound **9d** established critical hydrogen bond interaction with Trp481 and benzimidazole. Also, benzimidazole participated in pi–cation interaction with Arg600. On the other side of the molecule, 3-chlorophenylacetamide established H-bound interaction with Asp616 and halogen-bound interaction with Leu677. Notably, in most derivatives, the designed scaffold participated in the critical interactions within the active site of the enzyme and showed similar kinds of interactions to the native ligand.

## Conclusion

In this study, a series of novel quinoline-based-benzo[d]imidazole bearing different acetamide derivatives were designed, synthesized and their inhibitory activity against α-glucosidase was performed. Most of these derivatives showed increased activity compared to acarbose as the positive control. The analysis of the SAR indicated that *meta*-chlorine substitution, as well as polar group with potential hydrogen interactions at the R position, was beneficial to α-glucosidase inhibition. The most potent candidate in this series **9d** (IC_50_ = 3.2 ± 0.3 µM) was chosen for further biological evaluation. The enzyme kinetics assessments indicated that compound **9d** inhibited α-glucosidase in a competitive inhibition manner (*Ki* = 3.2 µM). According to the docking study, compound **9d** was well fitted in the active site of α-glucosidase through both hydrophobic and hydrogen interactions. Overall, it can be understood that the most potent derivative (**9d**) exhibited better inhibitory activity against a-glucosidase compared to lead compounds including **A**, to **G** reported compared to positive control reported in Fig. 1. In silico assessments confirmed the critical role of benzimidazole and aryl-acetamides to participate in interactions with the binding site of an enzyme.

Regarding that T2DM is public health concern nowadays, the inhibition of α-glucosidase is considered an efficient approach to target T2DM. It was shown that quinoline-based-benzo[d]imidazole bearing different acetamides constructed a new nucleus which provided a significant role for α-glucosidase inhibition. However, to better extract the SARs of this set of compounds, in the future project, heteroaryl or aliphatic substituents at the R position will be synthesized. Also, bioisosteric replacement of benzo[d]imidazole with other heteroaromatic rings will increase our insight into the design of more potent α-glucosidase inhibitors.

## Experimental

### Chemistry

All the reagents were purchased from commercial sources. ^1^H and ^13^C NMR spectra were determined by a Bruker FT-400 MHz spectrometer in DMSO-*d*_6_. All the chemical shifts were reported as (δ) values ppm. The MS spectra were recorded using an Agilent 7890A spectrometer at 70 eV. CHNOS analysis was performed using ECS4010 Costech Company. IR spectra were obtained with a Nicolet, FR -IR Magna 550. Melting-point were also recorded using Kofler hot-stage apparatus.

#### Synthesis of 2-chloroquinoline-3-carbaldehyde (2)^31^

To N, N-dimethylformamide (70.0 mmol) in the round-bottomed flask, phosphorus oxychloride (120.0 mmol) was added dropwise and the reaction mixture was stirred for 1 h at 0–5 °C. To this flask, N-phenylacetamide (30.0 mmol) was added and stirred for an extra 30 min followed by refluxing for 5–4 h under N_2_ atmosphere. After the reaction was completed (TLC monitoring), the mixture was poured into crushed ice under constant stirring. The precipitate obtained was vacuum filtered, washed with water, air-dried, and recrystallized from EtOAc to give the 2-chloroquinoline-3-carbaldehyde.

#### Synthesis of 2-mercaptoquinoline-3-carbaldehyde (3)^32^

The reaction was initiated by stirring the mixture of 2-chloroquinoline-3-carbaldehyde 2 (1 mmol) and sodium sulfide (1 mmol) for 2 h at room temperature in dry DMF (50 mL). Then, the reaction mixture was poured into crushed ice and made acidic with acetic acid. The product was filtered off, washed with water, and dried to give the desired 2-mercaptoquinoline-3-carbaldehyde that was further purified by recrystallization in ethanol.

#### Synthesis of 3-(1H-benzo[d]imidazol-2-yl)quinoline-2-thiol (5)^33^

2-Mercaptoquinoline-3-carbaldehyde (1 mmol) and o-phenylenediamine (1.2 mmol) were dissolved in 2 mL DMF. Under stirring at room temperature, 1 mmol of sodium metabisulfite is added and allowed to react at 120 °C for about 4 h. After completion of the reaction, the mixture was precipitated in ice water, filtered, and dried at room temperature.

#### Synthesis of 2-chloro-N-phenylacetamide derivatives (8a–r)^34^

To a solution of aniline derivatives (1 mmol) in DMF (4 mL), chloroacetylchloride was added at 0 °C. The mixture was stirred at room temperature for 5 h and poured into water and then filtered to get the **8a–r**. The obtained solids were then filtered, dried, and recrystallized from ethanol.

#### General method for synthesis of 2-((3-(1H-benzo[d]imidazol-2-yl)quinolin-2-yl)thio)-N-phenylacetamide derivatives (9a–r)^35^

A mixture of 3-(1H-benzo[d]imidazol-2-yl)quinoline-2-thiol (1 mmol) and potassium carbonate (1.5 mmol) in DMF were stirred at room temperature for 15–20 min. Afterward, *N*-chloroacetyl-aniline (1.2 mmol) was added to the above reaction mixture and stirred for an extra 4–5 h. After completion of the reaction, ice-cold water was added to the reaction mixture and stirred for 20 min. The obtained solid was filtered and washed with cold water several times. The acquired crude solid was purified by recrystallization from ethanol.

##### 2-((3-(1H-benzo[d]imidazol-2-yl)quinolin-2-yl)thio)-*N*-phenylacetamide (9a)

Brown solid; Yield: 93%; MP = 180–182 °C; IR (KBr, v_max_) 3310 (NH), 3025 (C–H Aromatic), 2970 (CH_2_ Aliphatic), 1675 (C=O) Cm^−1^; ^1^H NMR (400 MHz, DMSO-*d*_6_) δ 10.71 (s, 1H, NH), 9.07 (s, 1H, H_3_), 8.47 (s, 1H, H_4_), 8.19 (d, *J* = 8.00 Hz, 1H, H_8_), 8.10 (d, *J* = 8.60 Hz, 1H, H_5_), 7.86 (t, *J* = 8.40 Hz, 1H, H_7_), 7.81(d, *J* = 7.6 Hz, 2H, H_2_, H_6_), 7.65 (t, *J* = 8.00 Hz 1H, H_4_), 7.41 (t, *J* = 7.90 Hz, 2H, H_3_, H_5_), 7.16 (t, *J* = 7.40 Hz, 1H, H_3_) ppm. ^13^C NMR (100 MHz, DMSO-*d*_6_): δ 167.80, 157.98, 148.94, 147.10, 143.98, 139.79, 136.69, 135.03, 131.63, 129.24, 128.79, 127.65, 126.76, 125.08, 123.74, 123.63, 123.20, 123.40, 119.78, 119.48, 112.01, 36.55 ppm; ESI–MS (C_24_H_18_N_4_OS): calculated m/z 410.15 [M + H]^+^, observed m/z 410.10 [M + H]^+^ Anal. Calcd. For C_24_H_18_N_4_OS C, 70.22; H, 4.42; N, 13.65; Found: C, 70.39; H, 4.60; N, 13.76;

##### 2-((3-(1H-benzo[d]imidazol-2-yl)quinolin-2-yl)thio)-N-(2-fluorophenyl)acetamide (9b)

Brown solid; Yield: 87%; MP = 183–185 °C; IR (KBr, v_max_) 3325 (NH), 3060 (C–H Aromatic), 2950 (CH_2_ Aliphatic), 1680 (C=O) Cm^−1^; ^1^H NMR (400 MHz, DMSO-*d*_6_) δ 13.15 (s, 1H), 10.21 (s, 1H), 8.79 (s, 1H), 8.03 (d, *J* = 7.9 Hz, 1H), 7.98 (d, *J* = 8.4 Hz, 1H), 7.96–7.88 (m, 1H), 7.82 (t, *J* = 8.2 Hz, 1H), 7.61 (t, *J* = 7.4 Hz, 1H), 7.35–7.21 (m, 4H), 7.15–7.00 (m, 3H), 4.23 (s, 2H) ppm. ^13^C NMR (100 MHz, DMSO-*d*_6_): δ 168.45, 157.90, 154.95, 152.52 (CF, ^1^*J*_CF_ = 243 Hz), 148.91, 147.12, 136.82, 131.63, 128.77, 127.64, 126.90, 126.84, 126.78, 125.48, 125.40, 125.14, 124.86, 124.04, 123.22, 116.00, 115.81, 35.95 ppm; ESI–MS (C_24_H_17_FN_4_OS): calculated m/z 428.11 [M + H]^+^, observed m/z 428.20 [M + H]^+^, Anal. Calcd. for C_24_H_17_FN_4_OS: C, 67.27; H, 4.00; N, 13.08; Found: C, 67.45; H, 4.18; N, 13.24;

##### 2-((3-(1H-benzo[d]imidazol-2-yl)naphthalen-2-yl)thio)-N-(4-fluorophenyl)acetamide (9c)

Brown solid; Yield: 89%; MP = 189–191 °C; IR (KBr, v_max_) 3330 (NH), 3025 (C–H Aromatic), 2915 (CH_2_ Aliphatic),1640 (C=O) Cm^−1^; ^1^H NMR (400 MHz, DMSO-*d*_6_) δ 13.70 (s, 1H), 10.49 (s, 1H), 8.78 (s, 1H), 8.01 (d, *J* = 8.00 Hz, 1H), 7.92 (d, *J* = 8.30 Hz, 1H), 7.79 (t, *J* = 7.70 Hz, 1H), 7.73–7.56 (m, 5H), 7.33–7.26 (m, 2H), 7.15 (t, *J* = 8.8, 2H), 4.17 (s, 2H), ppm. ^13^C NMR (100 MHz, DMSO-*d*_6_): δ159.57, 157.95 (CF, ^1^*J*_CF_ = 238.1 Hz), 157.19, 148.96, 147.09, 136.68, 136.21, 131.63, 128.79, 127.3, 126.76, 125.08, 123.19, 121.25, 121.17, 115.92, 115.70, 36.49 ppm; ESI–MS (C_24_H_17_FN_4_OS): calculated m/z 428.11 [M + H]^+^, observed m/z 428.30 [M + H]^+^, Anal. Calcd. for C_25_H_18_FN_3_OS: C, 70.24; H, 4.24; N, 9.83; Found: C, 70.41; H, 4.47; N, 9.99.

##### 2-((3-(1H-benzo[d]imidazol-2-yl)quinolin-2-yl)thio)-N-(3-chlorophenyl)acetamide (9d)

Brown solid; Yield: 89%; MP = 190–192°; IR (KBr, v_max_) 3310 (NH), 3015 (C–H Aromatic), 2880 (CH_2_ Aliphatic), 1645 (C=O) Cm^−1^; ^1^H NMR (400 MHz, DMSO-*d*_6_) δ 13.6 (s, 1H), 10.65 (s, 1H), 8.79 (s, 1H), 8.01 (d, *J* = 8.10 Hz, 1H), 7.93–7.77 (m, 4H), 7.66–7.50 (m, 3H), 7.10 (d, *J* = 8.00 Hz, 1H), 4.18 (s, 3H) ppm. ^13^C NMR (100 MHz, DMSO-*d*_6_): δ 168.42, 167.60, 166.09, 162.75, 157.90, 150.04, 149.10, 147.06, 143.03, 141.29, 136.63, 136.21, 133.61, 131.56, 130.39, 128.76, 127.59, 126.73, 125.12, 123.23, 123.24, 123.02, 118.97, 117.86, 31.22 ppm; ESI–MS (C_24_H_17_ClN_4_OS): calculated m/z 444.08, [M + H]^+^, observed m/z 444.20, [M + H]^+^; Anal. Calcd. C_24_H_17_ClN_4_OS, C, 64.79; H, 3.85; N, 12.59; Found: C, 64.95; H, 4.02; N, 12.77.

##### 2-((3-(1H-benzo[d]imidazol-2-yl)quinolin-2-yl)thio)-N-(4-chlorophenyl)acetamide (9e)

Brown solid; Yield: 90%; MP = 185–187 °C IR (KBr, v_max_) 3275 (NH), 3030 (C–H Aromatic), 2980 (CH_2_ Aliphatic), 1680 (C=O) Cm^−1^; ^1^H NMR (400 MHz, DMSO-*d*_6_) δ 13.14 (s, 1H), 10.57 (s, 1H), 8.78 (s, 1H), 8.01 (d, *J* = 8.00 Hz, 1H), 7.89 (d, *J* = 8.40 Hz, 1H), 7.78 (t, *J* = 7.70 Hz, 2H), 7.68 (d, *J* = 8.50 Hz, 3H), 7.59 (t, *J* = 7.50 Hz, 1H), 7.37 (d, *J* = 8.50 Hz, 2H), 7.29 (d, *J* = 7.20 Hz, 2H) ppm. ESI–MS (C_24_H_17_ClN_4_OS): calculated m/z 444.08, [M + H]^+^, observed m/z 444.20,[M + H]^+^, ^13^C NMR (100 MHz, DMSO-*d*_6_): δ 168.06, 157.91, 148.93, 147.07, 138.77, 136.66, 131.64, 129.16, 128.79, 127.59, 127.14, 126.78, 125.05, 123.15, 120.99, 36.58 ppm; Anal. Calcd. for C_24_H_17_ClN_4_OS; C, 64.79; H, 3.85; N, 12.59; Found: C, 64.95; H, 3.99; N, 12.79.

##### 2-((3-(1H-benzo[d]imidazol-2-yl)quinolin-2-yl)thio)-N-(2-bromophenyl)acetamide (9f)

Brown solid; Yield: 93%; MP = 181–183 °C; IR (KBr, v_max_) 3300(NH), 3030 (C–H Aromatic), 2965 (CH_2_ Aliphatic), 1655 (C=O) Cm^−1^; ^1^H NMR (400 MHz, DMSO-*d*_6_) δ 13.14 (s, 1H), 10.43 (s, 1H), 8.77 (s, 1H), 8.01 (d, *J* = 7.90 Hz, 1H), 7.93 (d, *J* = 8.40 Hz, 1H), 7.80 (d, *J* = 5.50 Hz, 1H), 7.78 (d, *J* = 6.1 Hz, 1H), 7.67–7.56 (m, 3H), 7.35–7.24 (m, 4H), 7.04 (t, *J* = 7.3 Hz, 1H) ppm. ESI–MS (C_24_H_17_BrN_4_OS): calculated m/z 490.03, [M + H]^+^, observed m/z 488.10, [M + H]^+^, ^13^C NMR (100 MHz, DMSO-*d*_6_): δ 168.25, 157.53, 149.29, 147.09, 136.81, 136.69, 133.05, 131.53, 128.75, 128.52, 127.87, 126.91, 126.84, 125.93, 125.25, 123.60, 122.90, 116.69, 115.96, 35.84 ppm; Anal. Calcd. for C_24_H_17_BrN_4_OS C, 58.90; H, 3.50; N, 11.45; Found: C, 59.11; H, 3.69; N, 11.75.

##### 2-((3-(1H-benzo[d]imidazol-2-yl)quinolin-2-yl)thio)-N-(4-bromophenyl)acetamide (9g)

Brown solid; Yield: 95%; MP = 185–187°; IR (KBr, v_max_) 3340 (NH), 3025 (C–H Aromatic), 2870 (CH_2_ Aliphatic), 1640 (C=O) Cm^−1^; ^1^H NMR (400 MHz, DMSO-*d*_6_) δ 13.15 (s, 1H), 10.58 (s, 1H), 8.01 (d, *J* = 7.90 Hz, 1H), 7.90 (d, *J* = 8.10 Hz, 1H), 7.78 (t, *J* = 7.70 Hz, 2H), 7.64 (d, *J* = 8.50 Hz, 3H), 7.60–7.56 (m, 1H), 7.49 (d, *J* = 8.50 Hz 2H), 7.35–7.25 (m,2H), 4.17 (s, 2H) ppm. ESI–MS (C_24_H_17_BrN_4_OS): calculated m/z 490.03, [M + H]^+^, observed m/z 488.10, [M + H]^+^, ^13^C NMR (100 MHz, DMSO-*d*_6_): δ 168.10, 157.91, 148.94, 147.08, 143.98, 139.19, 136.66, 135.04, 132.07. 131.63, 128.78, 127.60, 126.77, 125.08, 123.76, 123.16, 122.41, 121.49, 121.40, 119.78, 115.18, 112.02, 36.62 ppm; Anal. Calcd. for C_24_H_17_BrN_4_OS; C, 58.90; H, 3.50; N, 11.45; Found: C, 59.05; H, 3.70; N, 11.62.

##### 2-((3-(1H-benzo[d]imidazol-2-yl)quinolin-2-yl)thio)-N-(2,6-dichlorophenyl)acetamide (9h)

Brown solid; Yield: 86%; MP = 188–190 °C; IR (KBr, v_max_) 3340 (NH), 3035 (C–H Aromatic), 2960 (CH_2_ Aliphatic), 1675 (C=O) Cm^−1^; ^1^H NMR (400 MHz, DMSO-*d*_6_) δ 13.15 (s, 1H), 9.92 (s, 1H), 8.81 (s, 1H), 8.03 (d, *J* = 8.00 Hz*,* 1H), 7.97 (d, *J* = 8.40 Hz, 1H), 7.86 (d, *J* = 8.80 Hz, 1H), 7.81 (t, *J* = 7.30 Hz, 1H), 7.75–7.57 (m, 4H), 7.37 (d, *J* = 8.80 Hz, 1H), 7.33–7.25 (m, 2H) 4.26 (s, 2H) ppm. ESI–MS (C_24_H_17_Cl_2_N_4_OS): calculated m/z 478.10, [M + H]^+^, observed m/z 444.20, [M + H]^+^, ^13^C NMR (100 MHz, DMSO-*d*_6_): δ 168.85, 157.60, 148.88, 147.10, 136.88, 134.69, 131.65, 129.30, 129.25, 128.79, 128.08, 127.77, 126.91, 126.45, 126.23, 125.19, 123.24, 35.80 ppm; Anal. Calcd. for C_24_H_16_Cl_2_N_4_OS C, 60.13; H, 3.36; N, 11.69; Found: C, 60.24; H, 3.49; N, 11.85.

##### 2-((3-(1H-benzo[d]imidazol-2-yl)quinolin-2-yl)thio)-N-(4-nitrophenyl)acetamide (9j)

Pale yellow solid; Yield: 93%; MP = 180–182 °C; IR (KBr, v_max_) 3320 (NH), 3020 (C–H Aromatic), 2965 (CH_2_ Aliphatic), 1670 (C=O), 1555–1350 (NO_2)_ Cm^−1^; ^1^H NMR (400 MHz, DMSO-*d*_6_) δ 13.60 (s, 1H), 11.80 (s, 1H), 8.79(s, 1H), 8.24 (d, *J* = 9.30 Hz, 1H), 8.01 (d, *J* = 7.70 Hz, 1H), 7.91 (d, J = 9.30, 2H), 7.86–7.74 (m, 3H), 7.63 (d, *J* = 7.70 Hz, 1H), 7.57 (t, *J* = 8.00 Hz, 1H), 7.35–7.25 (m, 2H), 4.21 (s, 2H) ppm. ESI–MS (C_24_H_17_N_5_O_3_S): calculated m/z 455.11, [M + H]^+^, observed m/z 455.20, [M + H]^+^, ^13^C NMR (100 MHz, DMSO-*d*_6_): δ 169.12, 157.79, 148.91, 147.01, 145.99, 143.96, 142.54, 136.61, 135.04, 131.67, 128.80, 127.49, 126.81, 125.60, 125.08, 123.79, 123.04, 122.43, 119.79, 119.08, 112.02, 36.80 ppm; ESI–MS (C_24_H_17_N_5_O_3_S C): calculated m/z 455.11 [M + H]^+^, observed m/z 455.20 [M + H]^+^; Anal. Calcd. for C_24_H_17_N_5_O_3_S C, 63.29; H, 3.76; N, 15.38; N, 7.31; Found: C, 63.49; H, 3.96; N, 15.55.

##### 2-((3-(1H-benzo[d]imidazol-2-yl)naphthalen-2-yl)thio)-N-(o-tolyl)acetamide (9k)

Brown solid; Yield: 86%; MP = 179–181 °C; IR (KBr, v_max_) 3360 (NH), 3070 (C–H Aromatic), 2980 (CH_2_ Aliphatic), 1680 (C=O) Cm^−1^; ^1^H NMR (400 MHz, DMSO-*d*_6_) δ 13.15 (s, 1H), 10.34 (s, 1H), 8.77 (s, 1H), 8.01 (d, *J* = 6.60 Hz, 1H), 7.93 (d, *J* = 7.10 Hz, 1H), 7.80 (d, *J* = 7.40 Hz, 2H), 7.66–7.46 (m, 4H), 7.30 (t, *J* = 9.50 Hz, 2H), 7.10 (d, *J* = 8.10 Hz, 2H), 4.15 (s, 2H), 2.20 (s, 3H), ppm. ^13^C NMR (100 MHz, DMSO-*d*_6_): δ 167.54, 158.01, 148.96, 147.11, 137.30, 136.70, 132.52, 131.61, 129.60, 128.77, 127.66, 126.74, 125.08, 123.23, 119.77, 119.51, 112.02, 36.54, 20.91 ppm; Anal. Calcd. for C_26_H_21_N_3_OS: C, 73.73; H, 5.00; N, 9.92; Found: C, 73.92; H, 5.19; N, 10.11.

##### 2-((3-(1H-benzo[d]imidazol-2-yl)naphthalen-2-yl)thio)-N-(p-tolyl)acetamide (9l)

Cream solid; Yield: 91%; MP = 181–183 °C; IR (KBr, v_max_) 3345 (NH), 3040 (C–H Aromatic), 2900 (CH-Aliphatic), 1670 (C=O) Cm^−1^; ^1^H NMR (400 MHz, DMSO-*d*_6_) δ 13.15 (s, 1H), 10.34 (s, 1H), 8.77 (s, 1H), 8.01 (d, *J* = 7.30 Hz, 1H), 7.93 (d, *J* = 7.70 Hz, 1H), 7.79 (t, *J* = 7.70 Hz, 2H), 7.63–7.48 (m, 4H), 7.29 (s, 2H), 7.10 (d, *J* = 8.20 Hz, 2H), 4.16 (s, 2H), 2.23 (s, 3H) ppm. ESI–MS (C_26_H_21_N_3_OS): calculated m/z 424.14, [M + H]^+^, observed m/z 424.10, [M + H]^+ 13^C NMR (100 MHz, DMSO-*d*_6_): δ 162.6, 160.2, 147.4, 141.2, 140.1, 136.5, 133.7, 132.2, 131.0, 129.4, 128.4, 128.3, 126.2, 126.0, 124.0, 120.9, 28.1, 16.1 ppm; Anal. Calcd. for C_26_H_21_N_3_OS C, 73.73; H, 5.00; N, 9.92; Found: C, 73.82; H, 5.14; N, 9.99.

##### 2-((3-(1H-benzo[d]imidazol-2-yl)quinolin-2-yl)thio)-N-(4-methoxyphenyl)acetamide (9m)

Cream solid; Yield: 93%; MP = 191–193 °C; IR (KBr, v_max_) 3340 (NH), 3030 (C–H Aromatic), 2910 (CH-Aliphatic), 1680 (C=O) Cm^−1^; ^1^H NMR (400 MHz DMSO-*d*_6_) δ 13.14 (s, 1H), 10.27 (s, 1H), 8.77 (s, 1H), 8.02 (d, *J* = 7.70 Hz, 1H), 7.95 (d, *J* = 8.40 Hz, 1H), 7.80 (t, *J* = 8.30 Hz, 1H), 7.59 (t, *J* = 7.90 Hz, 1H), 7.54 (d, *J* = 9.00 Hz, 2H), 7.34–7.25 (m, 2H), 6.88 (d, *J* = 9.10 Hz, 1H), 4.15 (s, 2H), 3.71 (s, 3H) ppm. ^13^C NMR (100 MHz, DMSO-*d*_6_): δ 167.23, 158.02, 155.60, 148.96, 147.12, 136.71, 132.94, 131.62, 128.78, 127.68, 126.75, 125.08, 123.24, 121.03, 114.33, 55.58, 36.43 ppm; ESI–MS (C_25_H_20_N_4_O_2_S): calculated m/z 424.14 [M + H]^+^, observed m/z 424.10 [M + H]^+^; Anal. Calcd. for C_25_H_20_N_4_O_2_S; C, 68.16; H, 4.58; N, 12.72; Found C, 68.35; H, 4.76; N, 12.90.

##### 2-((3-(1H-benzo[d]imidazol-2-yl)quinolin-2-yl)thio)-N-(4-ethylphenyl)acetamide (9n)

Brown solid;Yield:93%;MP = 178–180 °C; IR (KBr, v_max_) 3300 (NH), 3020 (C–H Aromatic), 2975(CH_2_ Aliphatic), 1670 (C=O) Cm^−1^; ^1^H NMR (400 MHz, DMSO-*d*_6_) δ 13.12 (s, 1H), 10.34 (s, 1H), 8.78 (s, 1H), 8.01 (d, *J* = 8.00 Hz, 1H), 7.95 (d, *J* = 8.40 Hz, 1H), 7.79 (t, *J* = 7.30 Hz, 1H), 7.75–7.65 (m, 2H), 7.58 (t, *J* = 7.60 Hz, 1H), 7.54 (d, *J* = 8.40 Hz, 2H), 7.30 (d, *J* = 6.10 Hz, 2H), 7.13 (d, *J* = 8.30 Hz, 2H), 4.17 (s, 2H), 2.53 (d, *J* = 7.70 Hz, 2H), 1.14 (t, *J* = 7.30 Hz, 3H) ppm. ESI–MS (C_26_H_22_N_4_OS): calculated m/z 438.55, [M + H]^+^, observed m/z 438.10,[M + H]^+^, ^13^C NMR (100 MHz, DMSO-*d*_6_): δ 167.55, 158.00, 148.97, 147.11, 139.01, 137.47, 136.70, 131.61, 128.78, 128.41, 127.68, 126.75, 125.08, 123.24, 119.61, 36.52, 28.06, 16.18, 16.12 ppm; Anal. Calcd. for C_26_H_22_N_4_OS; C, 71.21; H, 5.06; N, 12.78; Found: C, 71.39; H, 5.26; N, 12.97.

##### 2-((3-(1H-benzo[d]imidazol-2-yl)quinolin-2-yl)thio)-N-(2,3-dimethylphenyl)acetamide (9o)

Brown solid; Yield: 93%; MP = 185–187 °C IR; (KBr, v_max_) 3300 (NH), 3020 (C–H Aromatic), 2975 (CH_2_ Aliphatic) 1675 (C=O) Cm^−1^; ^1^H NMR (400 MHz, DMSO-*d*_6_) δ 13.6 (s, 1H), 9.76 (s, 1H), 8.79 (s, 1H), 8.03 (d, *J* = 8.90 Hz, 1H), 8.01 (d, *J* = 7.90 Hz, 1H), 7.83 (t, *J* = 8.30 Hz, 1H), 7.61 (t, *J* = 7.90 Hz, 1H), 7.30 (d, *J* = 6.60, 2H), 7.15 (d, *J* = 7.50 Hz, 1H), 7.06–6.92 (m, 4H), 4.22 (s, 1H), 2.20 (s, 3H), 2.02 (s, 3H), ppm. ^13^C NMR (100 MHz, DMSO-*d*_6_): δ 167.73, 157.98, 149.00, 147.19, 137.38, 136.87, 136.66, 131.60, 131.57, 128.81, 127.77, 127.33, 126.77, 125.62, 125.15, 123.78, 123.39, 35.74, 20.59, 14.47 ppm; ESI–MS (C_26_H_22_N_4_OS): calculated m/z 438.15 [M + H]^+^, observed m/z 438.10 [M + H]^+^; Anal. Calcd. for C_26_H_22_N_4_OS C, 71.21; H, 5.06; N, 12.78; Found C, 71.39; H, 5.26; N, 12.91.

##### 2-((3-(1H-benzo[d]imidazol-2-yl)quinolin-2-yl)thio)-N-(2,6-dimethylphenyl)acetamide (9p)

Brown solid; Yield: 93%; MP = 185–187 °C; IR (KBr, v_max_) 3325 (NH), 3045 (C–H Aromatic), 2980(CH_2_ Aliphatic) 1665 (C=O) Cm^−1^; ^1^H NMR (400 MHz, DMSO-*d*_6_) δ 13.11 (s, 1H), 9.57 (s, 1H), 8.77 (s, 1H), 8.03 (t, *J* = 8.40 Hz, 2H), 7.84 (t, *J* = 7.70 Hz, 1H), 7.62 (t, *J* = 7.20 Hz, 2H), 7.35–7.25 (m, 2H), 7.07–6.98 (m, 4H), 4.24 (s, 1H), 2.05 (s, 6H) ppm. ESI–MS (C_26_H_22_N_4_OS): calculated m/z 438.15 [M + H]^+^, observed m/z 438.10 [M + H]^+^; ^13^C NMR (100 MHz, DMSO-*d*_6_): δ 162.6, 161.6, 147.4, 140.9, 139.5, 133.7, 132.7, 132.3, 131.0, 129.4, 128.9, 128.3, 127.9, 127.5, 126.3, 126.0, 123.3, 43.3 ppm; Anal. Calcd. for C_26_H_22_N_4_OS C, 71.21; H, 5.06; N, 12.78; Found C, 71.39; H, 5.26; N, 12.91.

##### 2-((3-(1H-benzo[d]imidazol-2-yl)quinolin-2-yl)thio)-N-(naphthalen-2-yl)acetamide (9q)

Cream solid; Yield: 91%; MP = 182–184° C; IR (KBr, v_max_) 3340 (NH), 3030 (C–H Aromatic), 2900 (CH-Aliphatic), 1670 (C=O) Cm^−1^; ^1^H NMR (400 MHz, DMSO-*d*_6_) δ 13.16 (s, 1H), 10.33 (s, 1H), 8.80 (s, 1H), 8.10 (d, *J* = 8.30 Hz, 1H), 8.05 (d, *J* = 7.90 Hz, 2H), 7.92 (d, *J* = 7.8 Hz, 1H), 7.86–7.80 (m, 1H), 7.79–7.66 (m, 3H), 7.63 (d, *J* = 7.70 Hz, 2H), 7.53–7.44 (m, 2H), 7.37 (t, *J* = 7.60 Hz, 1H), 7.34–7.24 (m, 2H), 4.36 (s, 2H), ppm. ESI–MS (C_28_H_20_N_4_OS): calculated m/z 460.14 [M + H]^+^, observed m/z 460.10 [M + H]^+^; ^13^C NMR (100 MHz, DMSO-*d*_6_): δ 168.50, 149.01, 147.21, 136.87, 134.23, 134.13, 131.61, 128.84, 128.54, 128.40, 127.79, 126.80, 126.46, 126.10, 126.06, 125.83, 125.18, 123.40, 122.18, 35.94 ppm; Anal. Calcd. for C_28_H_20_N_4_OS; C, 73.02; H, 4.38; N, 12.17; Found: C, 73.32; H, 4.55; N, 12.34.

##### 2-((3-(1H-benzo[d]imidazol-2-yl)quinolin-2-yl)thio)-N-benzylacetamide (9r)

Brown solid; Yield: 94%; MP = 183–185 °C; IR (KBr, v_max_) 3310(NH) , 3045(C-H Aromatic), 2975 (CH_2_ Aliphatic) 1655 (C=O) Cm^−1^; ^1^H NMR (400 MHz, DMSO-*d*_6_) δ 13.11 (s, 1H), 8.76 (s, 1H), 8.71 (t, *J* = 6.00 Hz, 1H), 8.03 (d, *J* = 8.00 Hz, 1H), 7.90 (d, *J* = 8.5 Hz, 1H), 7.81 (t, *J* = 7.70 Hz 1H), 7.62 (t, *J* = 7.60 Hz, 2H), 7.34–7.25 (m, 2H), 7.21–7.13 (m, 6H), 4.31 (d, *J* = 6.00 Hz, 2H), 4.06 (s,1H) ppm. ESI–MS (C_26_H_22_N_4_OS): calculated m/z 424.14 [M + H]^+^, observed m/z 424.10 [M + H]^+^; ^13^C NMR (100 MHz, DMSO-*d*_6_): δ 168.67, 157.83, 149.00, 147.15, 139.83, 136.79, 131.51, 128.73, 128.55, 127.90, 127.45, 127.05, 126.73, 125.10, 123.39, 42.83, 35.17 ppm; Anal. Calcd. for C_25_H_20_N_4_OS: C, 70.73; H, 4.75; N, 13.20; Found: C, 70.92; H, 4.90; N, 13.38.

##### 2-((3-(1H-benzo[d]imidazol-2-yl)quinolin-2-yl)thio)-N-(4-fluorobenzyl)acetamide (9s)

Brown solid;Yield:89%; MP = 186–188 °C; IR (KBr, v_max_) 3350 (NH), 3060 (C–H Aromatic), 2975 (CH_2_ Aliphatic), 1670 (C=O) cm^−1^; ^1^H NMR (400 MHz, DMSO-*d*_6_) δ 8.74 (d, *J* = 14.20 Hz, 2H), 8.01 (d, *J* = 8.00 Hz, 1H), 7.90–7.75 (m, 2H), 7.70 (s, 2H), 7.60 (s, 1H), 7.39–7.17 (m, 4H), 6.95 (t, *J* = 8.9, 2H), 4.29 (s, 2H), 4.04 (s, 2H), ppm. ESI–MS (C_25_H_19_FN_4_OS): calculated m/z 442.14 [M + H]^+^, observed m/z 442.10 [M + H]^+^; ^13^C NMR (100 MHz, DMSO-*d*_6_): δ 168.75, 162.67, 160.27 (CF, ^1^*J*_CF_ = 248.00 Hz), 157.81, 149.10, 147.10, 136.74, 136.04, 131.43, 129.49, 129.41, 128.71, 127.84, 126.71, 125.10, 123.45, 122.98, 115.30, 115.10, 42.20, 35.21 ppm; Anal. Calcd. for C_25_H_19_FN_4_OS: C, 67.86; H, 4.33; N, 12.66; Found: C, 68.04; H, 4.52; N, 12.81.

### α-Glucosidase inhibitory assay

The α-glucosidase inhibitory activities of all synthesized derivatives were assayed according to the previously reported procedure^9,36^.

### Enzyme kinetic studies

The mode of inhibition of the most active compound (**9c**), identified with the lowest IC_50_, was investigated against α-glucosidase at different concentrations of *p*-nitrophenyl *α*-d-glucopyranoside (4–16 mM) as substrate in the absence and presence of **9c** at different concentrations (0, 0.8, 1.6, and 3.2 µM). A Lineweaver–Burk plot was generated to identify the type of inhibition and the Michaelis–Menten constant (*K*_m_) value was determined from the plot between reciprocal of the substrate concentration (1/[S]) and reciprocal of enzyme rate (1/V) over various inhibitor concentrations. The experimental inhibitor constant (*K*_i_) value was constructed by secondary plots of the inhibitor concentration [I] versus *K*_m_^6^.

### Molecular docking

To perform the molecular modeling investigations, the Maestro Molecular Modeling platform (version 10.5) by Schrödinger, LLC was used. The X-ray crystal structure of the receptor was downloaded from the PDB database (PDB ID: 5NN8). The protein is then prepared using a protein preparation wizard. At this point, all water molecules and co-crystallized ligands were removed, the missing side chains and loops were filled using the prime tool, and PROPKA assigned H-bonds at pH 7.4. To prepare the ligands, the 2D structures of the ligands were drawn in ChemDraw (ver. 16) and converted into SDF files, which were used further by the ligprep module. Ligands were prepared by OPLS_2005 force field using EPIK at a target pH of 7.0 ± 2. The grid box was generated for each binding site using entries with a box size of 25 Å, all derivatives were docked on binding sites using induced-fit docking, reporting 10 poses per ligand to form the final complex^9,37^.

## Supplementary Information

Supplementary Information.

## Data Availability

All data generated or analyzed during this study are included in this published article and its Supplementary Information files.
