# Supplementary material for: Design, synthesis, and in silico studies of quinoline-based-benzo[d]imidazole bearing different acetamide derivatives as potent α-glucosidase inhibitors
Source: Sci Rep. 2022 Aug 18;12:14019. doi: 10.1038/s41598-022-18455-7 (PMC9386204; doi:10.1038/s41598-022-18455-7)

^{1}H-NMR of 2-((3-(1H-benzo[d]imidazol-2-yl)quinolin-2-yl)thio)-N-phenylacetamide (9a)

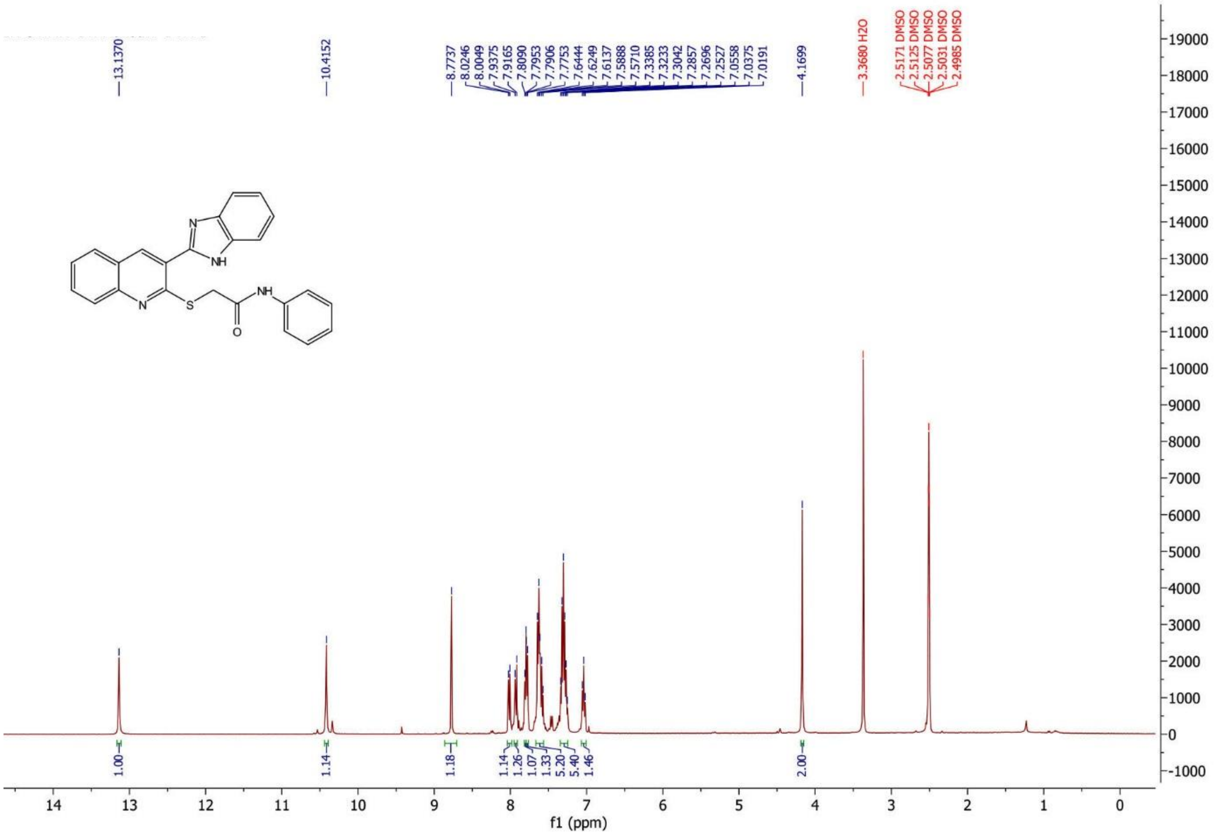

^{13}C-NMR of 2-((3-(1H-benzo[d]imidazol-2-yl)quinolin-2-yl)thio)-N-phenylacetamide (9a)

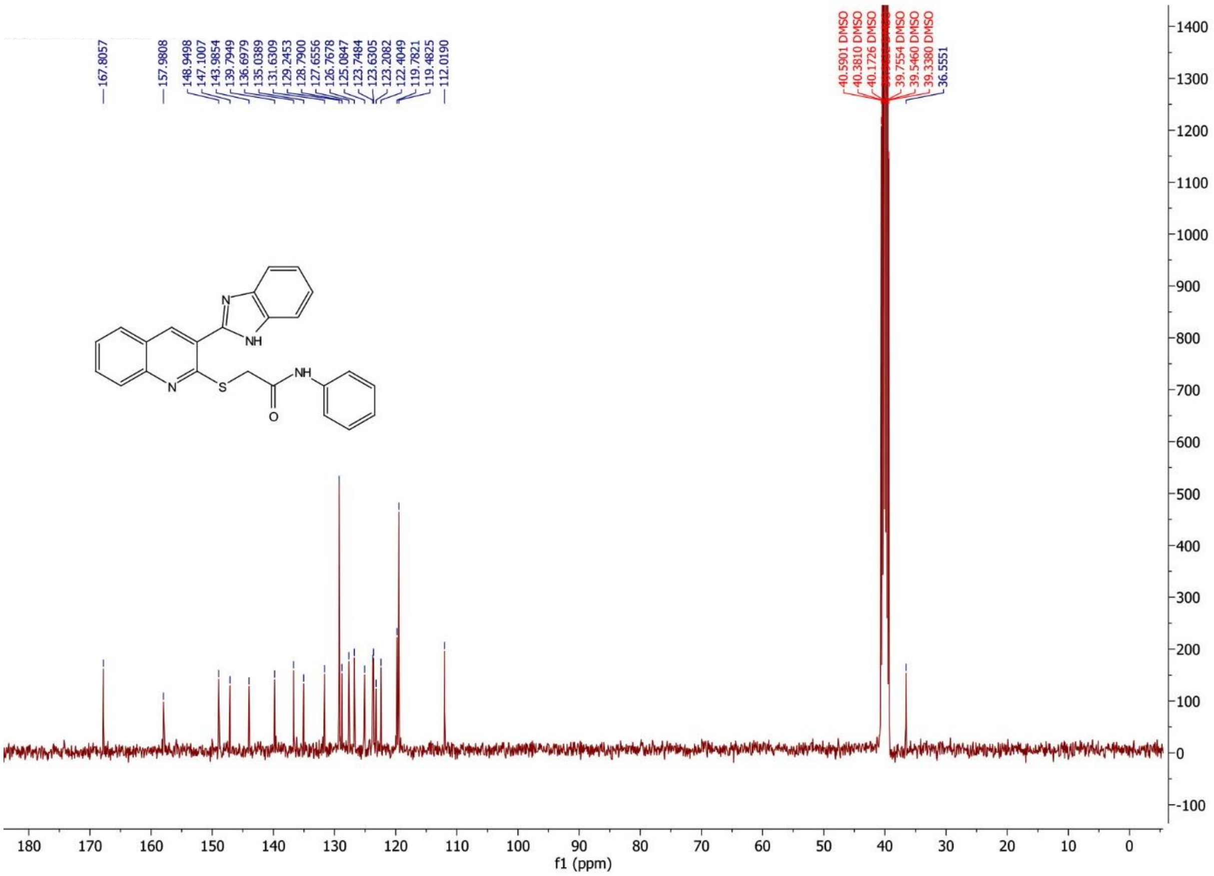

Mass of 2-((3-(1H-benzo[d]imidazol-2-yl)quinolin-2-yl)thio)-N-phenylacetamide (9a)

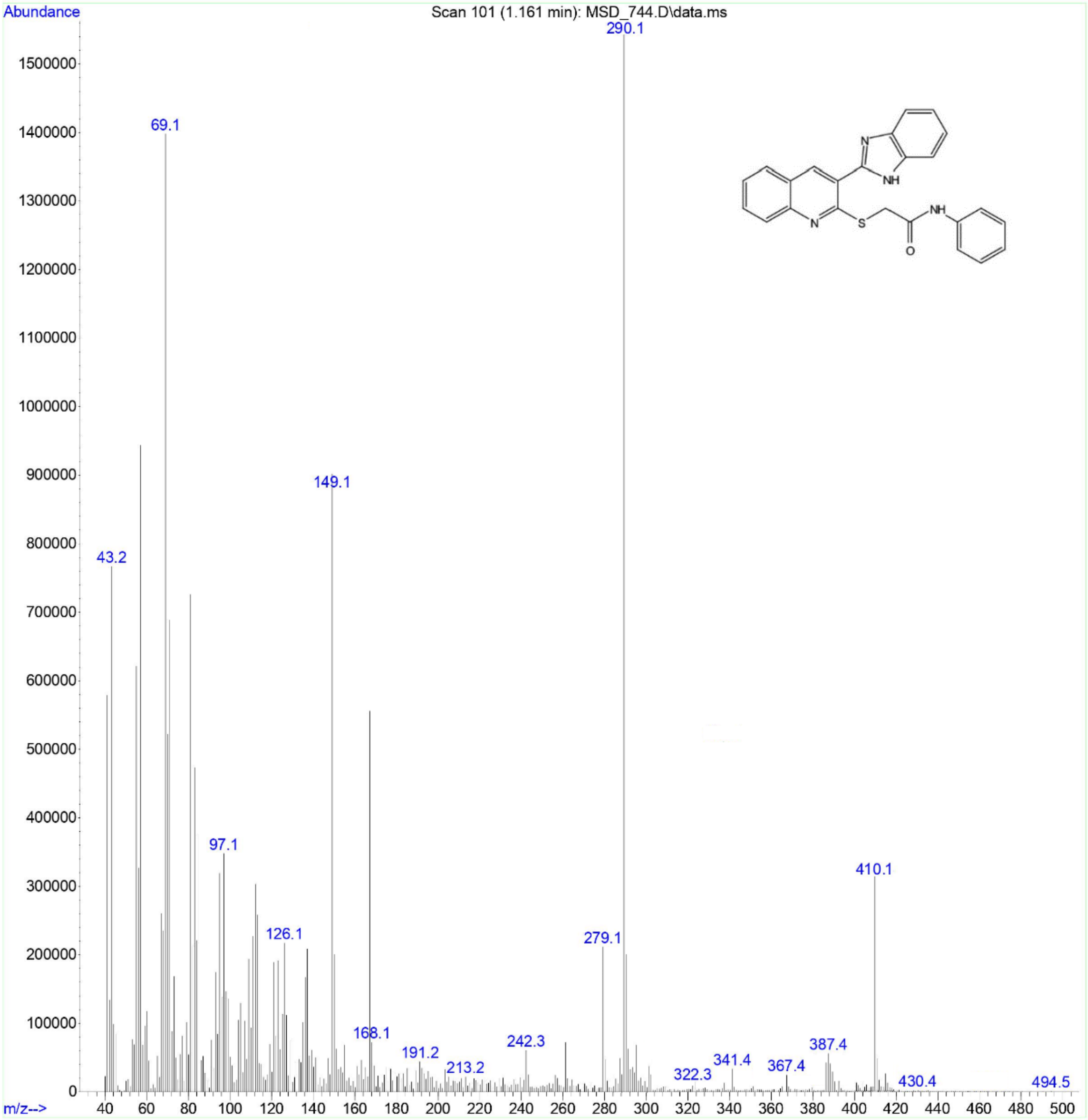

^{1}H-NMR of 2-((3-(1H-benzo[d]imidazol-2-yl)quinolin-2-yl)thio)-N-(2-fluorophenyl)acetamide (9b)

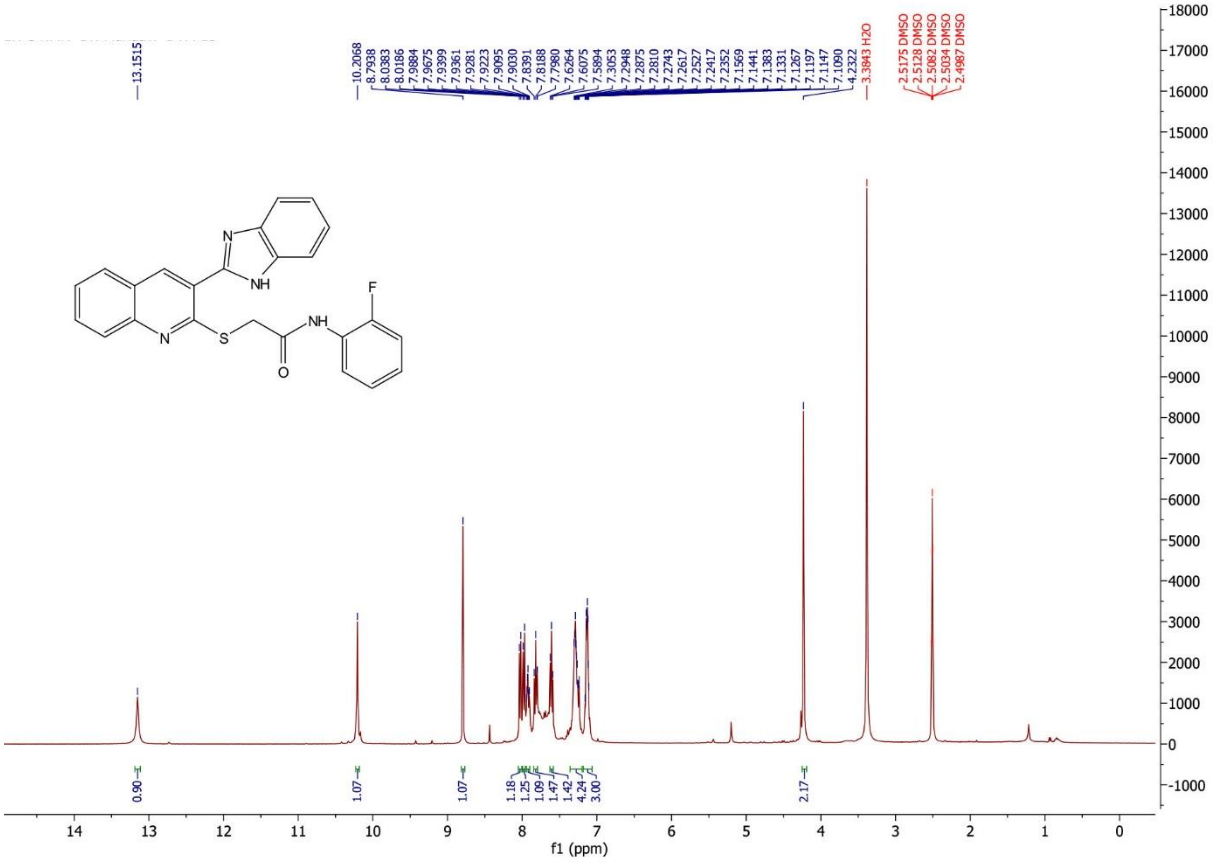

[13] C-NMR of 2-((3-(1H-benzo[d]imidazol-2-yl)quinolin-2-yl)thio)-N-(2-fluorophenyl)acetamide (9b)

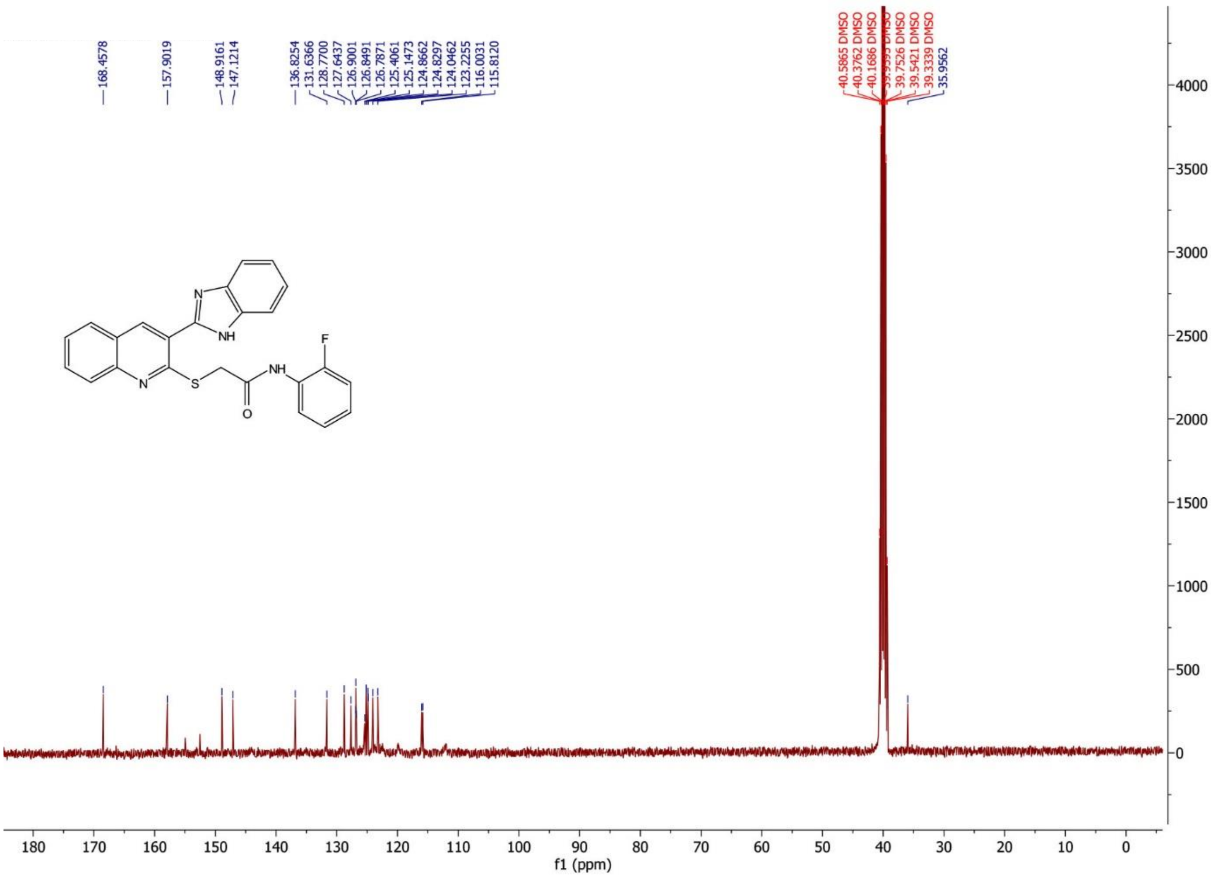

Mass of 2-((3-(1H-benzo[d]imidazol-2-yl)quinolin-2-yl)thio)-N-(2-fluorophenyl)acetamide (9b)

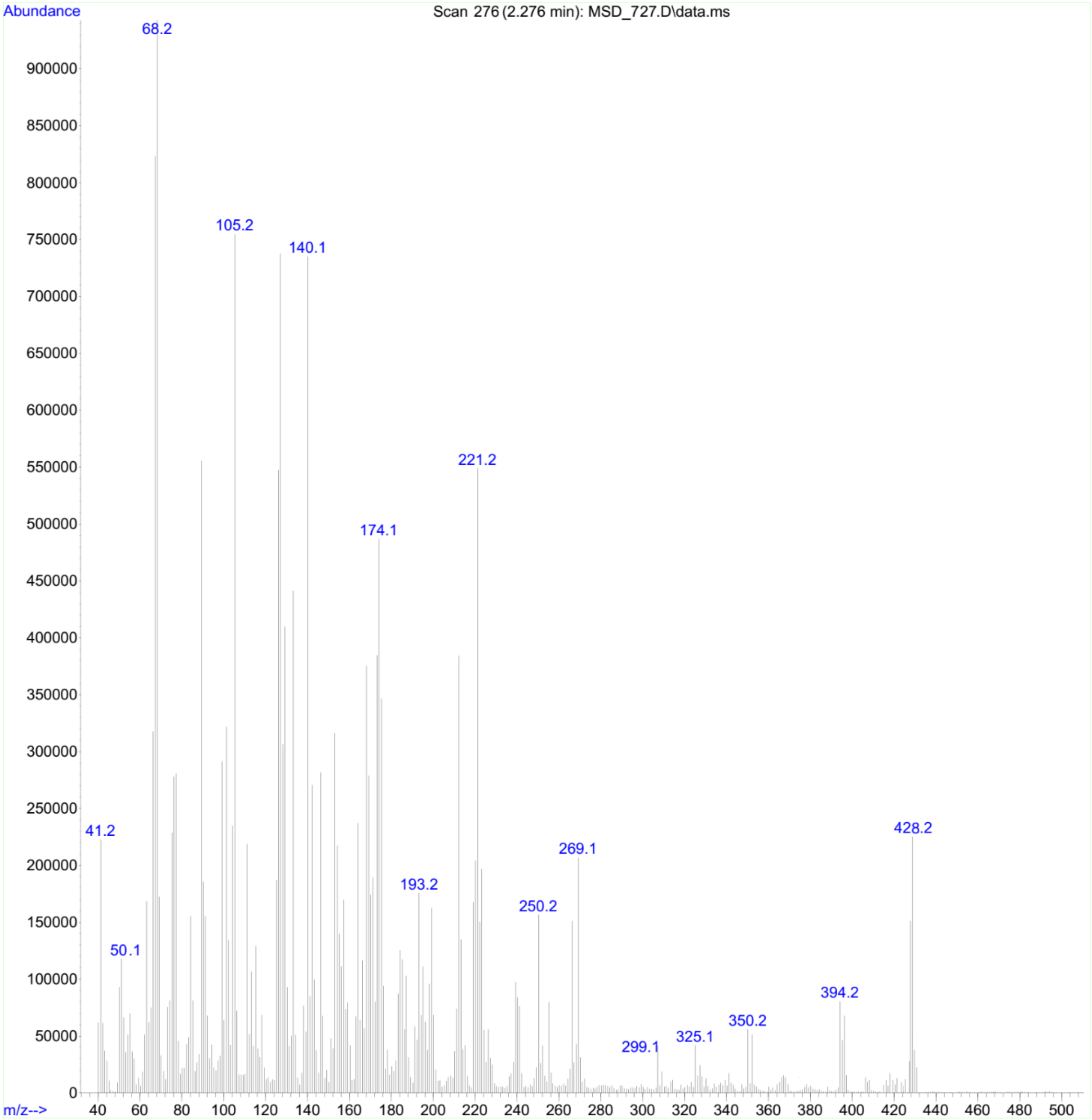

^{1}H-NMR of 2-((3-(1H-benzo[d]imidazol-2-yl)quinolin-2-yl)thio)-N-(4-fluorophenyl)acetamide

(9c)

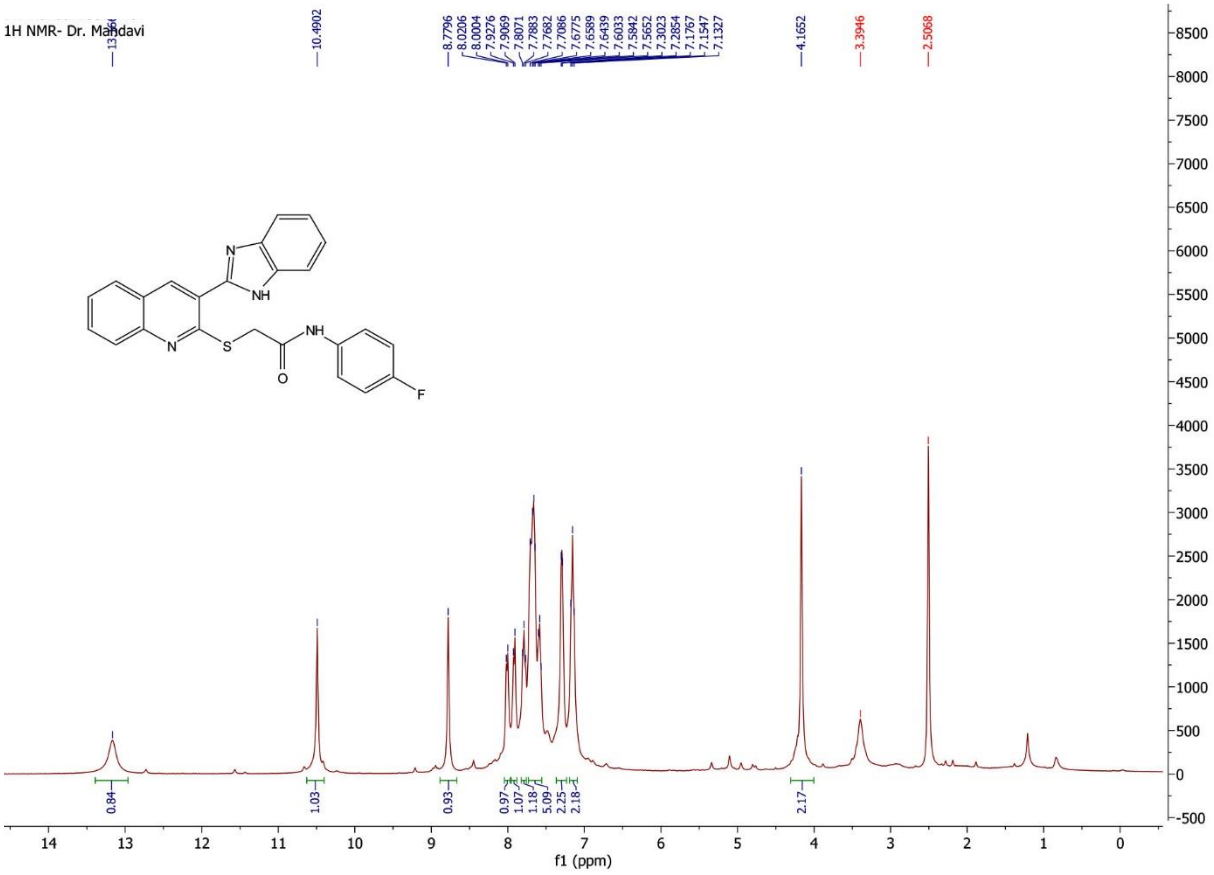

^{13}C-NMR of 2-((3-(1H-benzo[d]imidazol-2-yl)quinolin-2-yl)thio)-N-(4-fluorophenyl)acetamide

(9c)

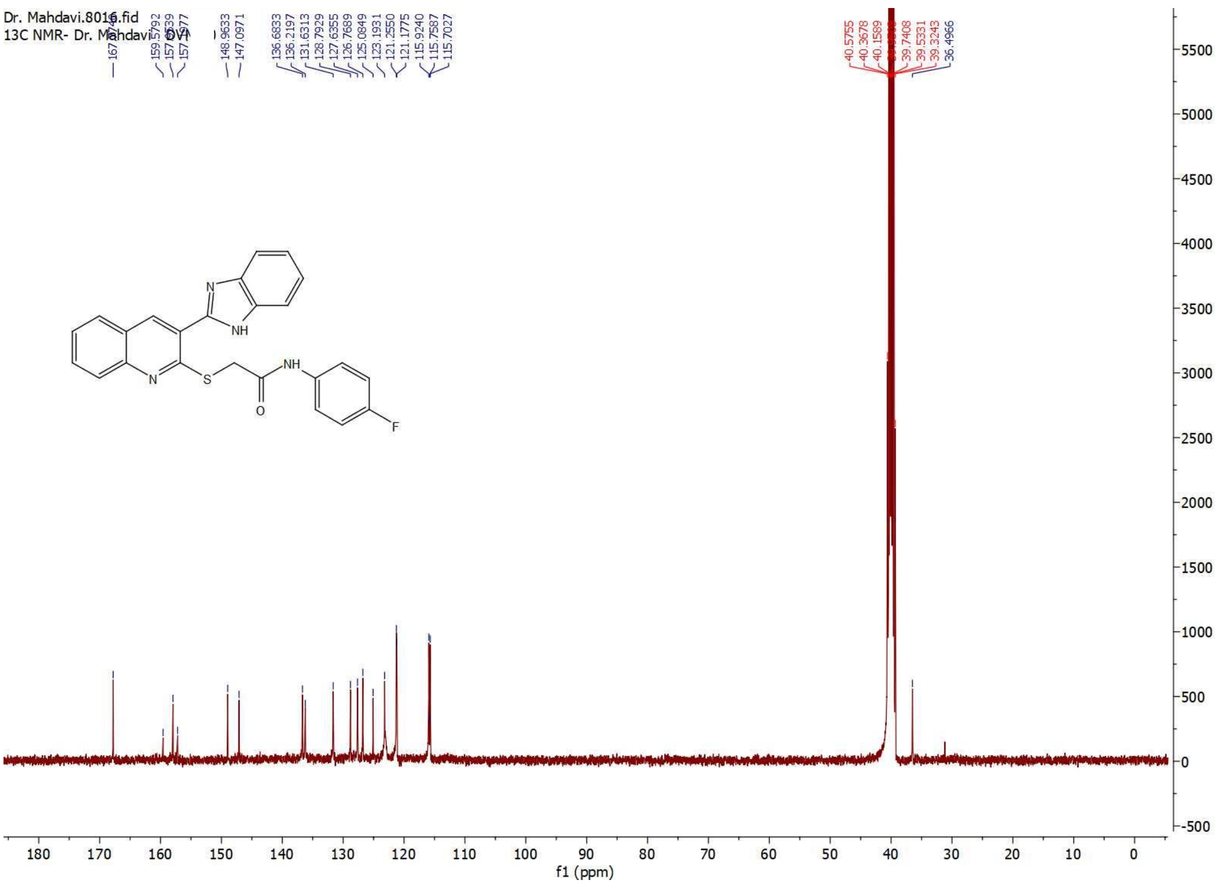

*Mass of 2-((3-(1H-benzo[d]imidazol-2-yl)quinolin-2-yl)thio)-N-(4-fluorophenyl)acetamide (**9c**)*

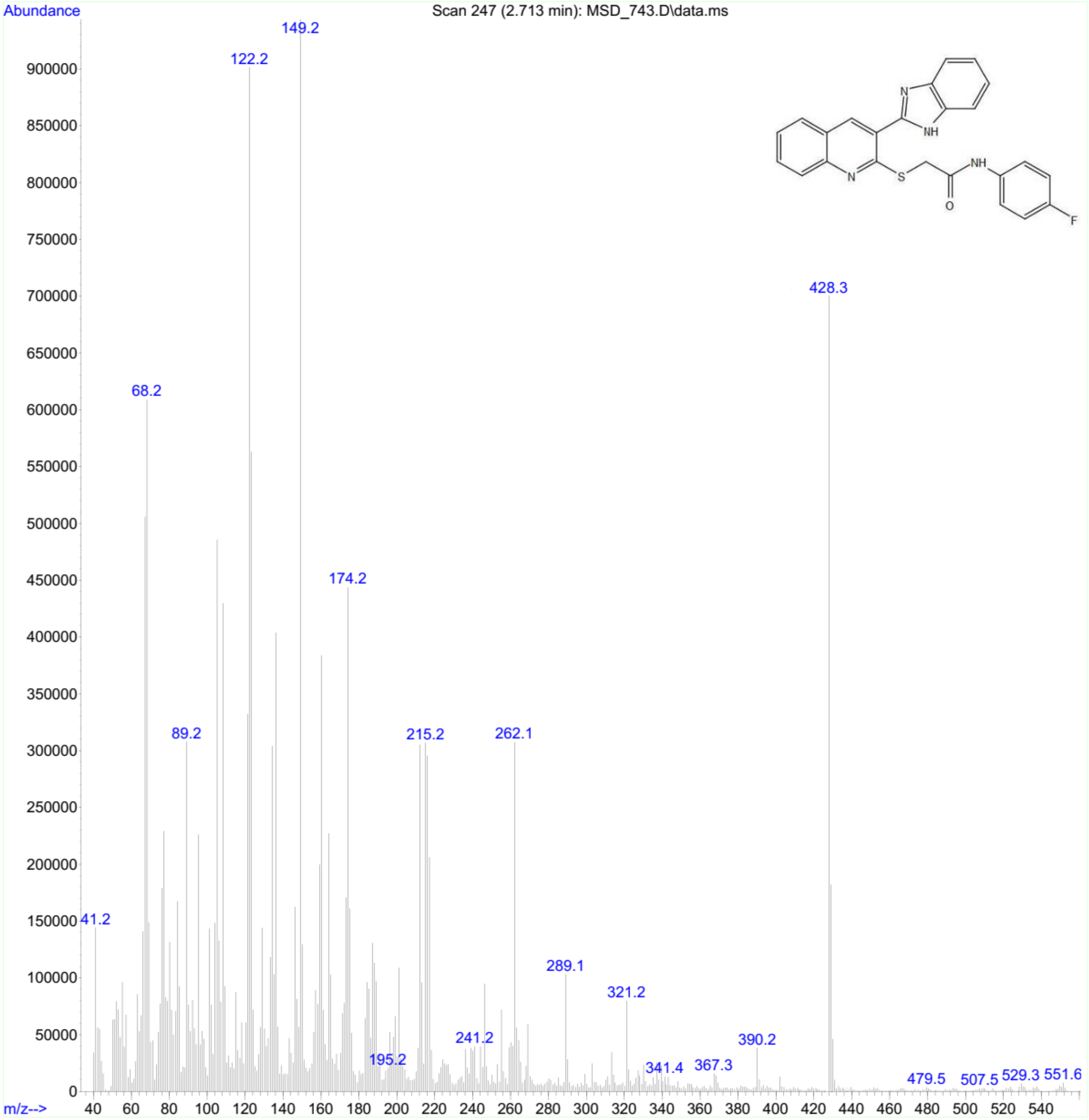

^{1}H-NMR of 2-((3-(1H-benzo[d]imidazol-2-yl)quinolin-2-yl)thio)-N-(3-chlorophenyl)acetamide ***(9d)***

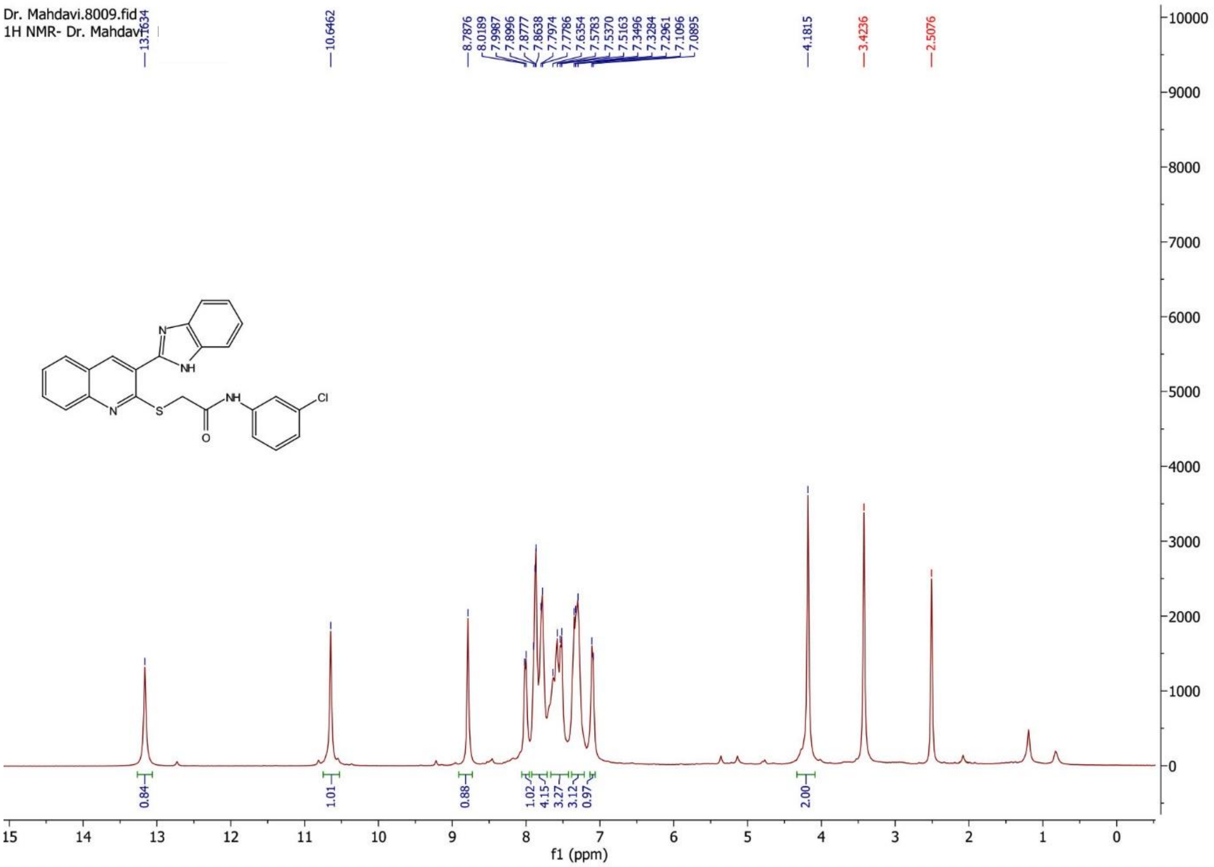

^{13}C-NMR of 2-((3-(1H-benzo[d]imidazol-2-yl)quinolin-2-yl)thio)-N-(3-chlorophenyl)acetamide

(9d)

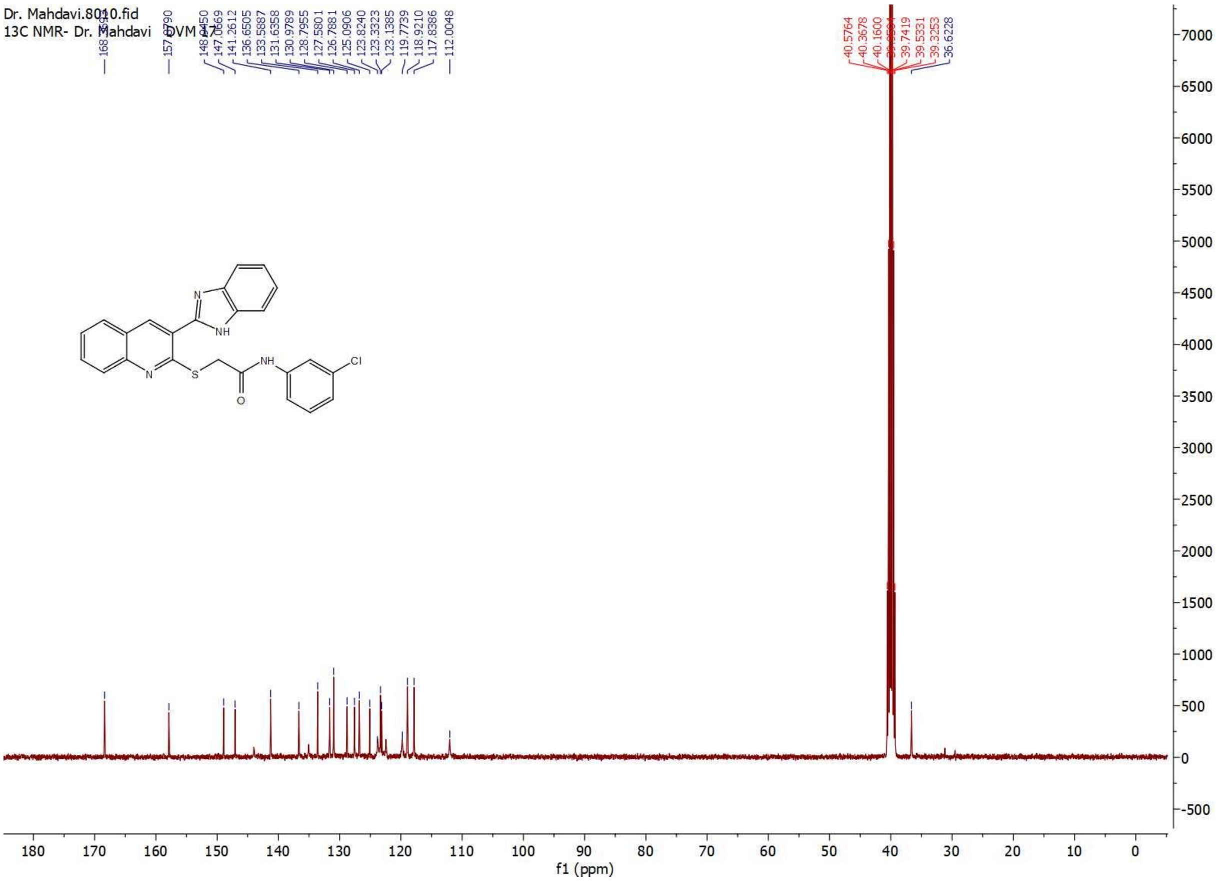

*Mass of 2-((3-(1H-benzo[d]imidazol-2-yl)quinolin-2-yl)thio)-N-(3-chlorophenyl)acetamide (**9d**)*

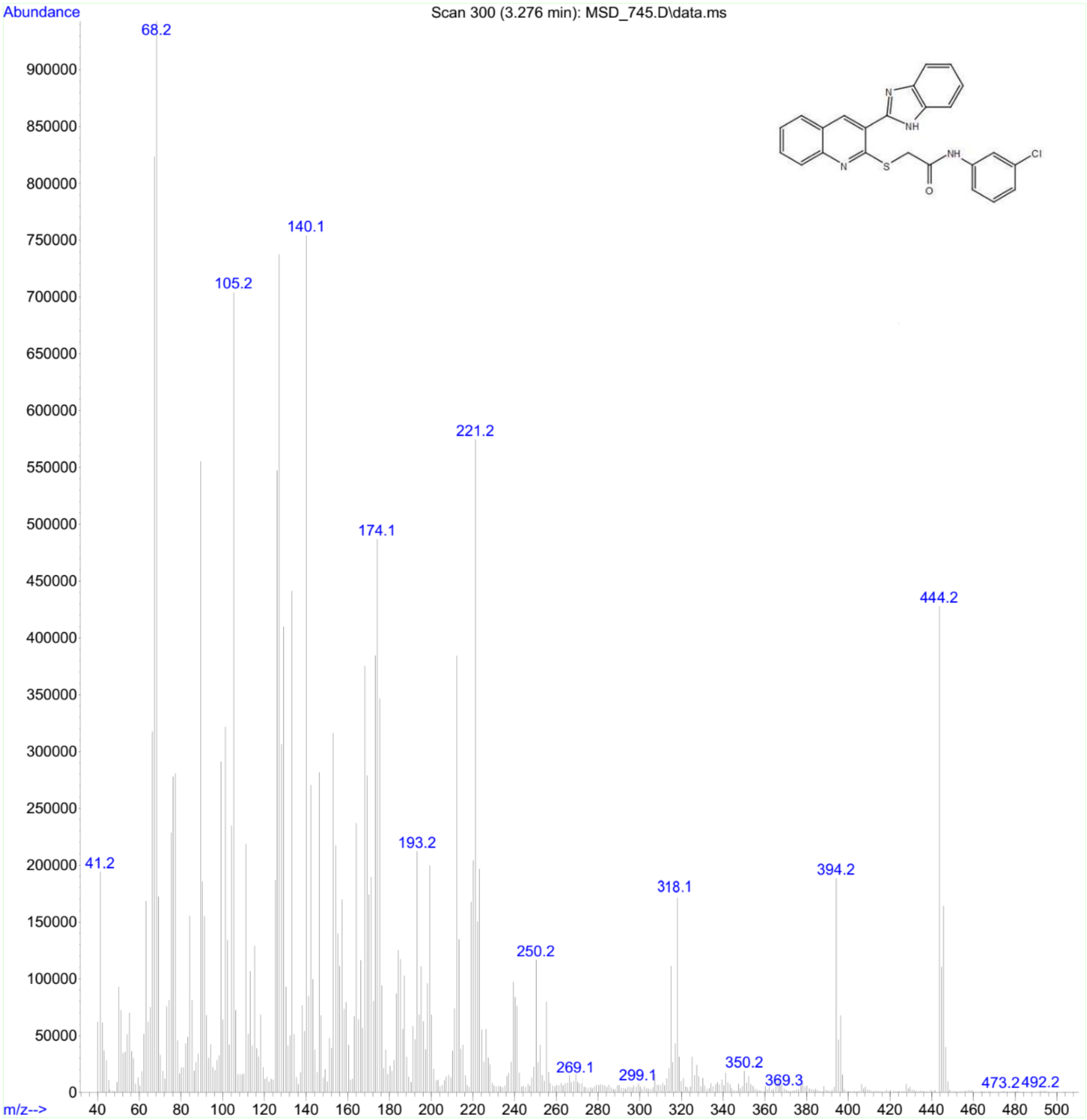

^{1}H-NMR of 2-((3-(1H-benzo[d]imidazol-2-yl)quinolin-2-yl)thio)-N-(4-chlorophenyl)acetamide

(9e)

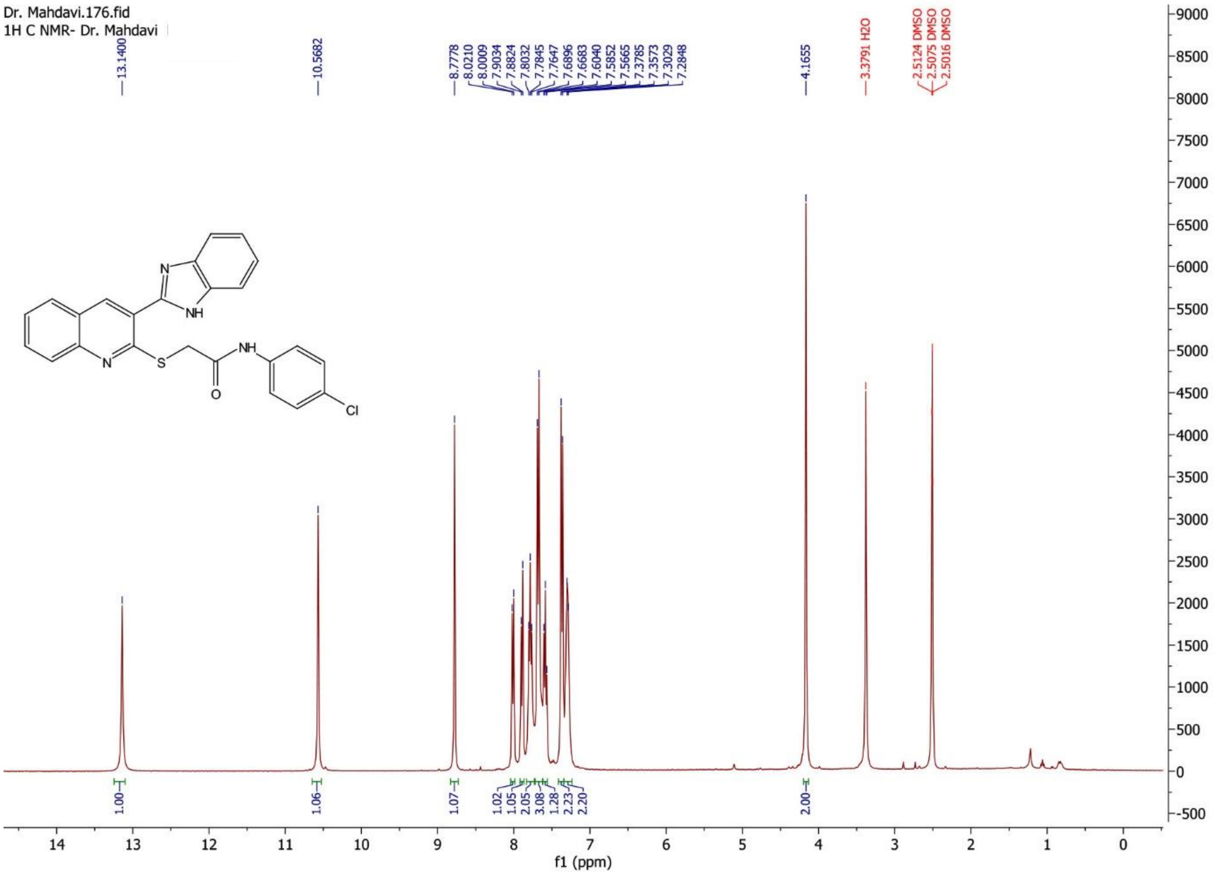

^{13}C-NMR of 2-((3-(1H-benzo[d]imidazol-2-yl)quinolin-2-yl)thio)-N-(4-chlorophenyl)acetamide

(9e)

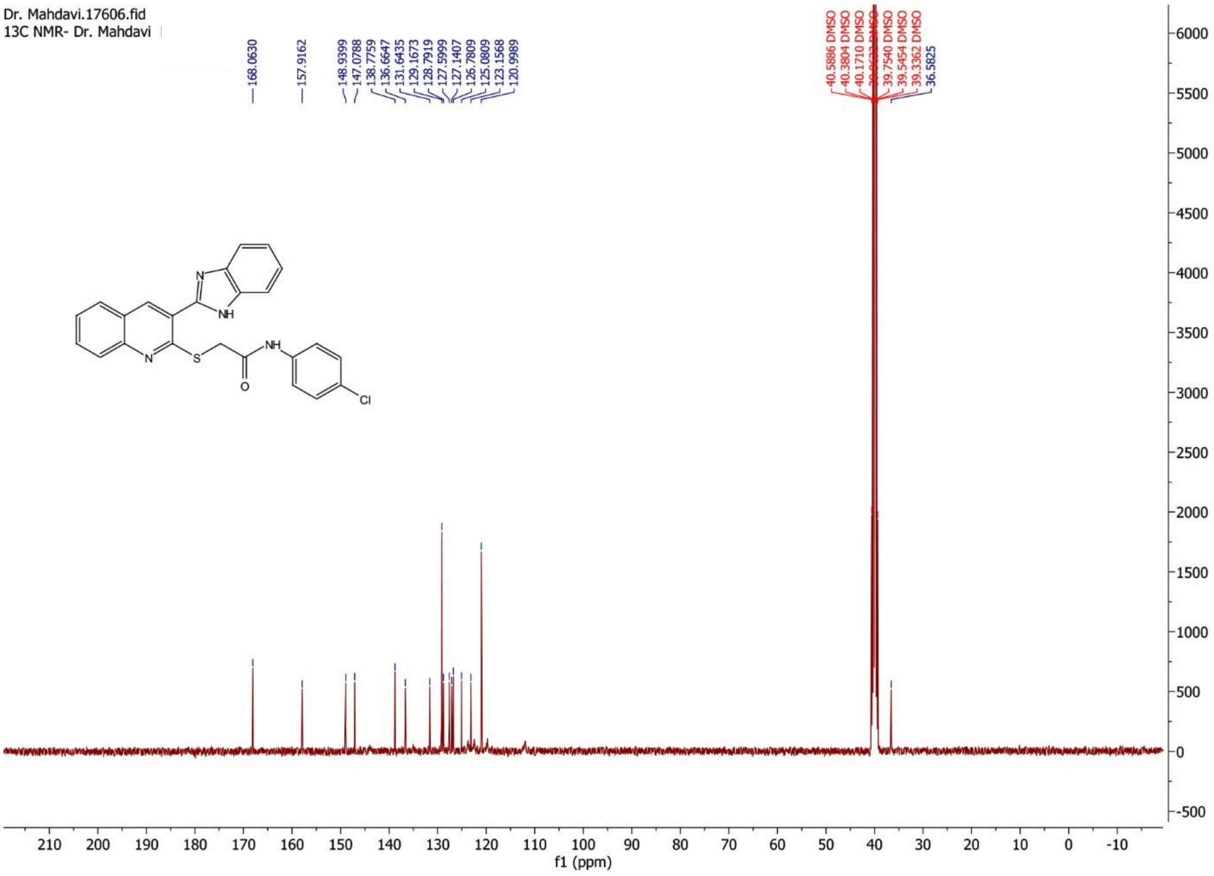

*Mass of 2-((3-(1H-benzo[d]imidazol-2-yl)quinolin-2-yl)thio)-N-(4-chlorophenyl)acetamide (**9e**)*

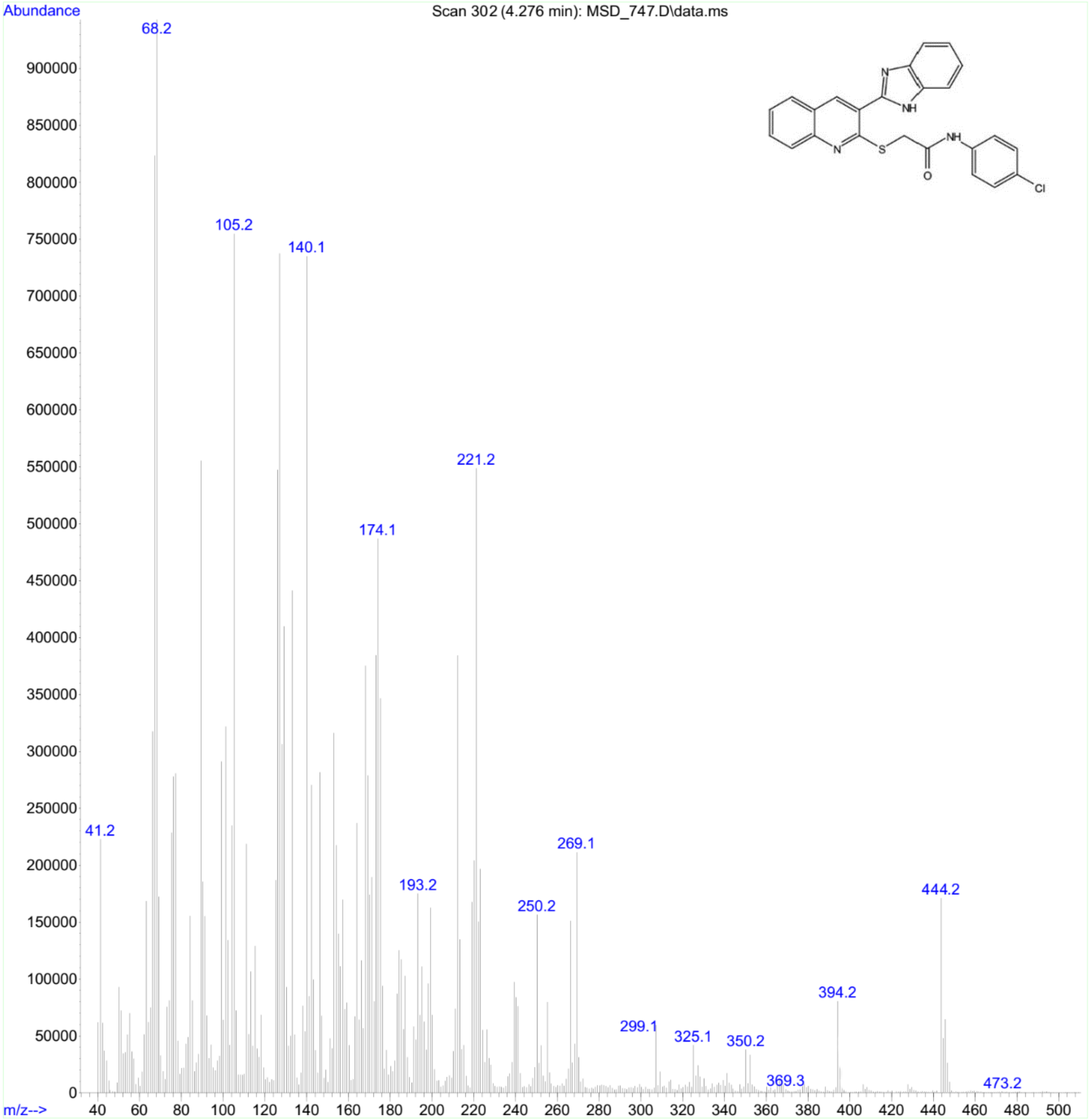

^{1}H-NMR of 2-((3-(1H-benzo[d]imidazol-2-yl)quinolin-2-yl)thio)-N-(2-bromophenyl)acetamide ***(9f)***

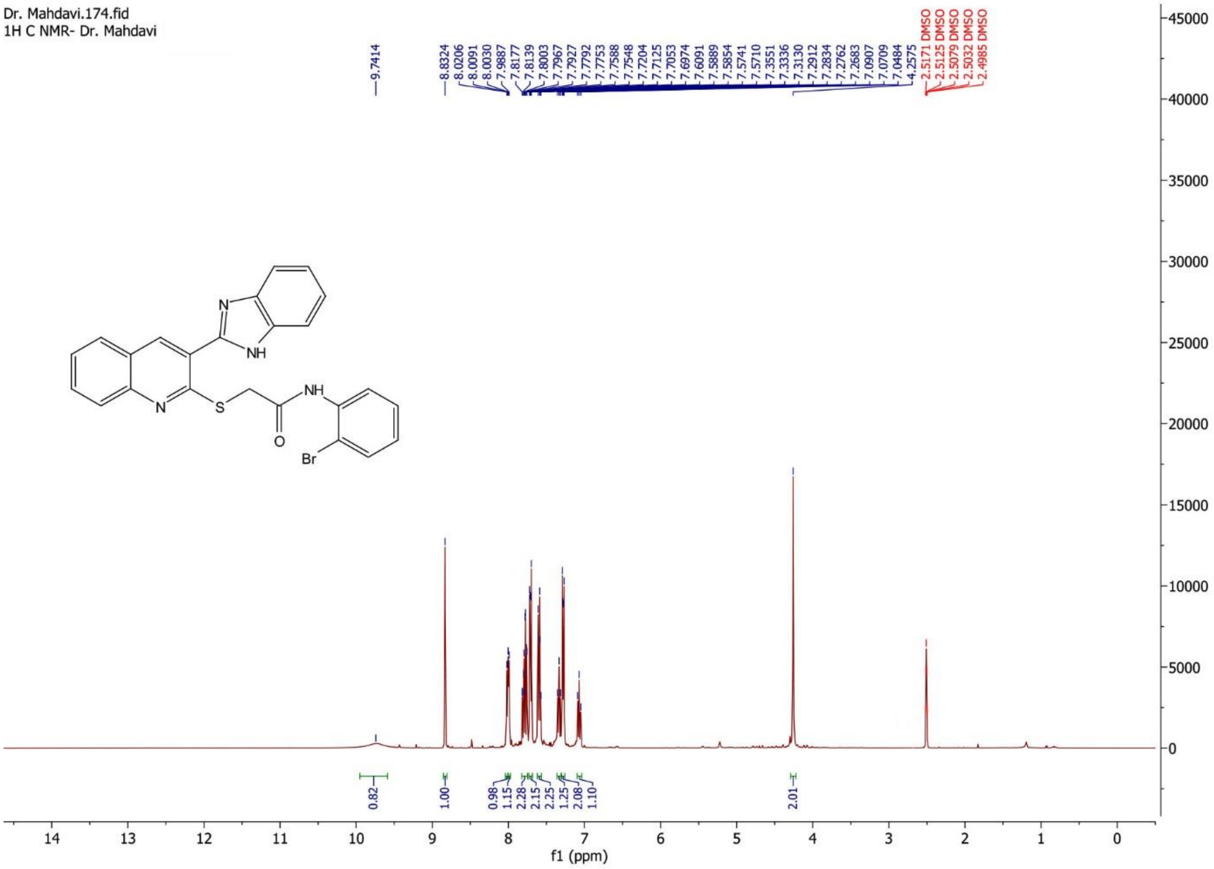

^{13}C-NMR of 2-((3-(1H-benzo[d]imidazol-2-yl)quinolin-2-yl)thio)-N-(2-bromophenyl)acetamide ***(9f)***

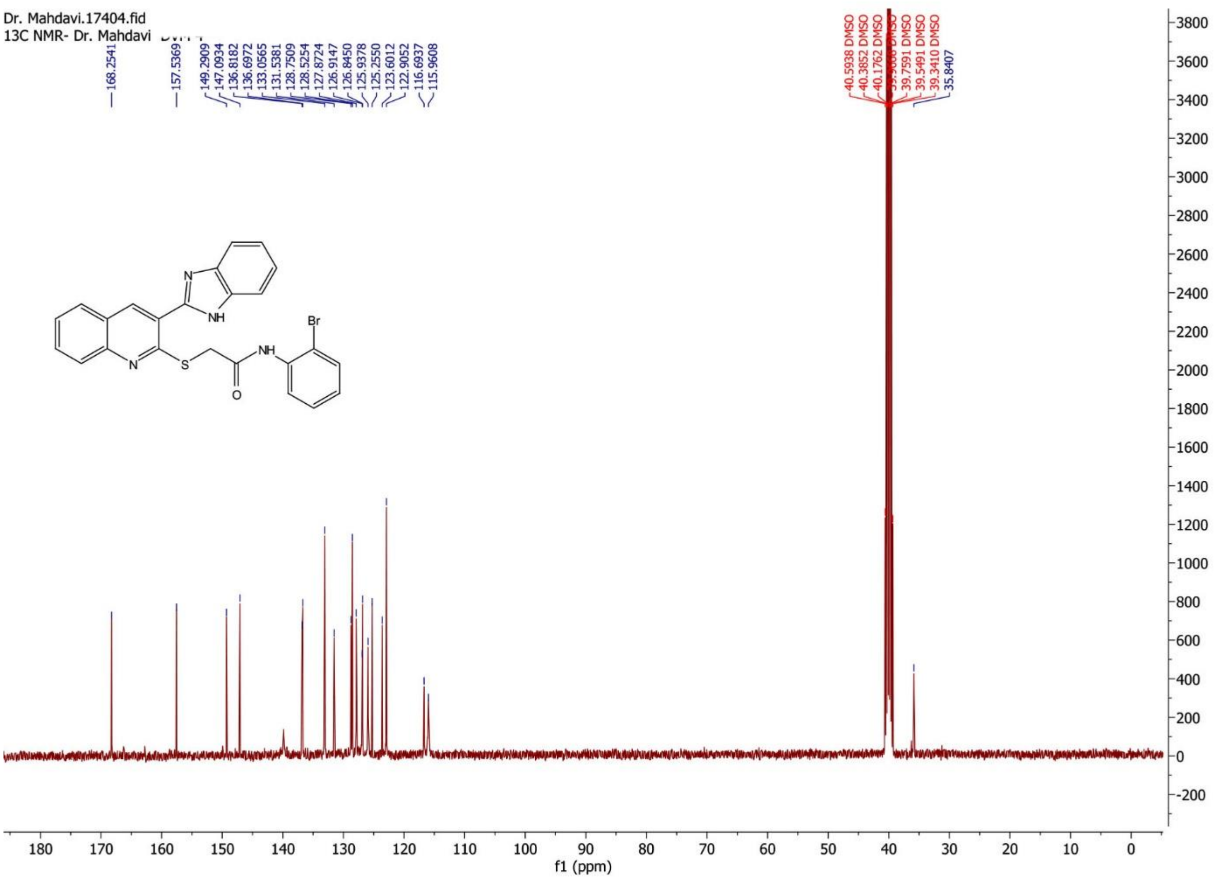

*Mass of 2-((3-(1H-benzo[d]imidazol-2-yl)quinolin-2-yl)thio)-N-(2-bromophenyl)acetamide (**9f**)*

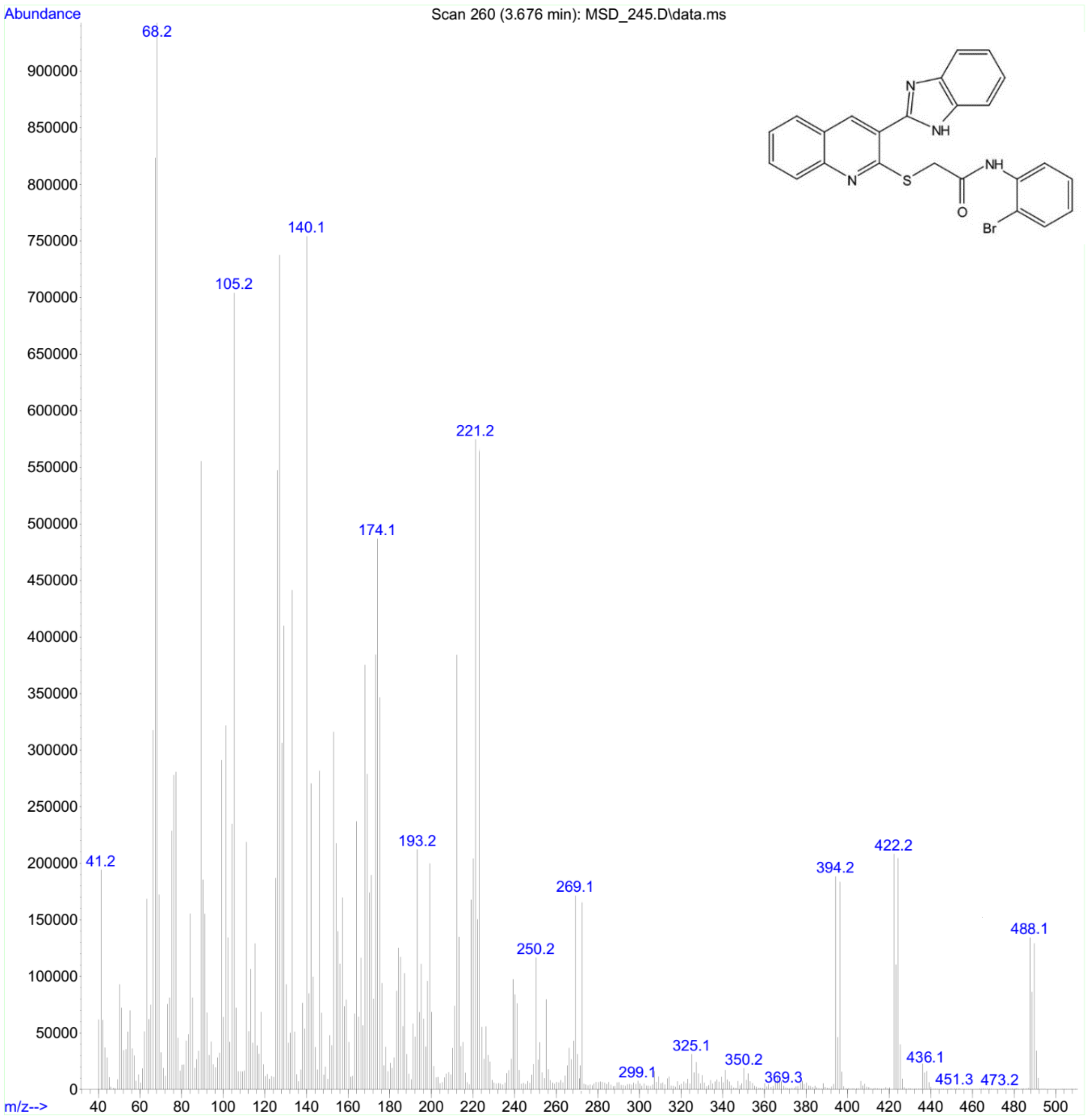

^{1}H-NMR of 2-((3-(1H-benzo[d]imidazol-2-yl)quinolin-2-yl)thio)-N-(4-bromophenyl)acetamide

(9g)

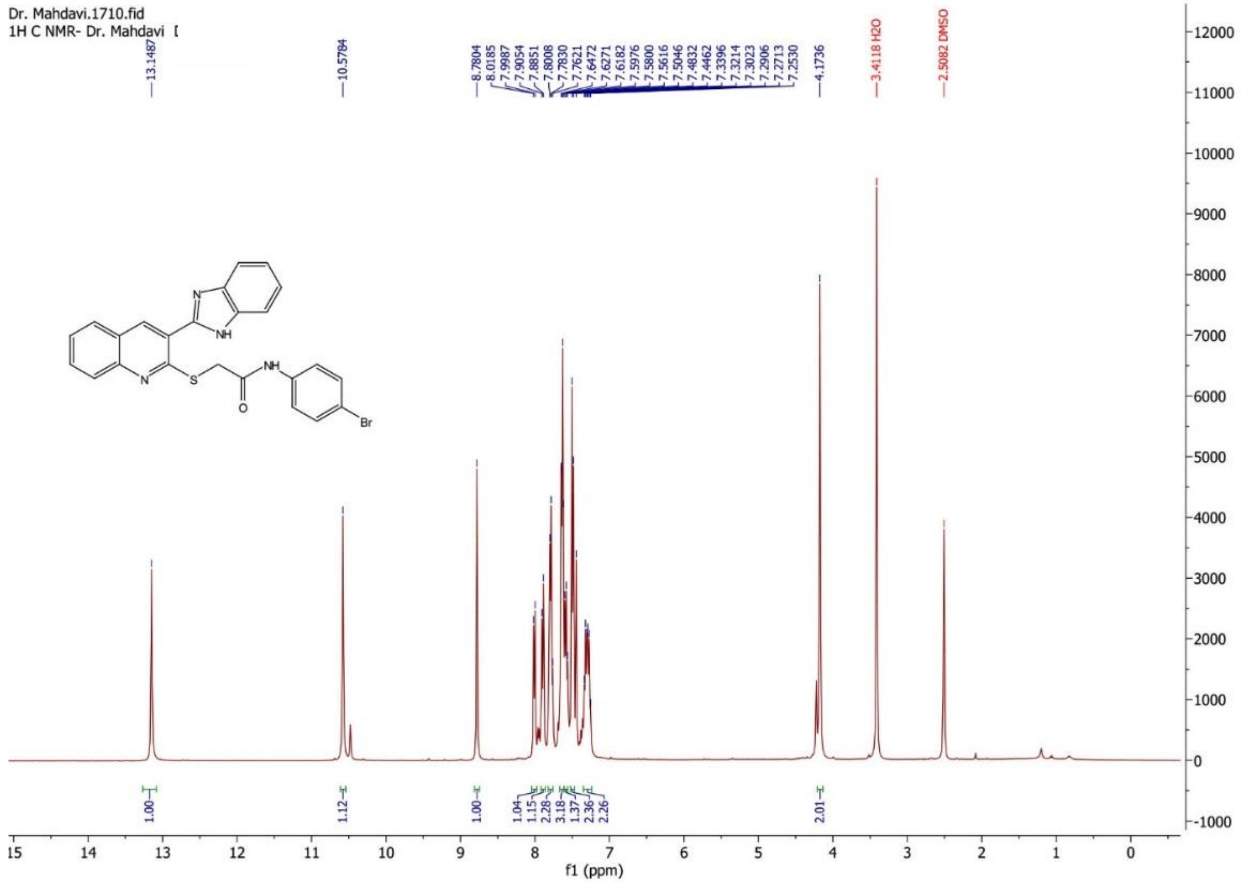

^{13}C-NMR of 2-((3-(1H-benzo[d]imidazol-2-yl)quinolin-2-yl)thio)-N-(4-bromophenyl)acetamide

(9g)

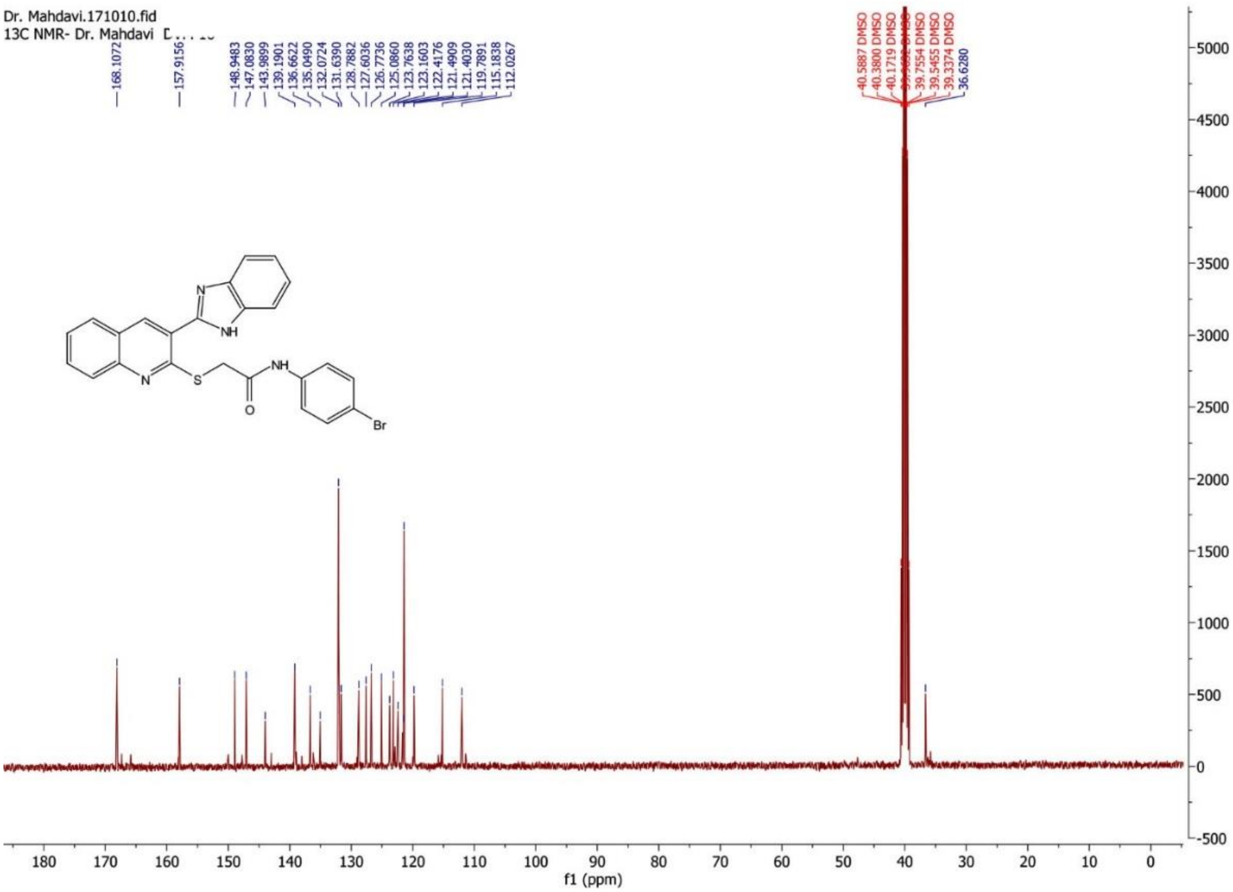

*Mass of 2-((3-(1H-benzo[d]imidazol-2-yl)quinolin-2-yl)thio)-N-(4-bromophenyl)acetamide (**9g**)*

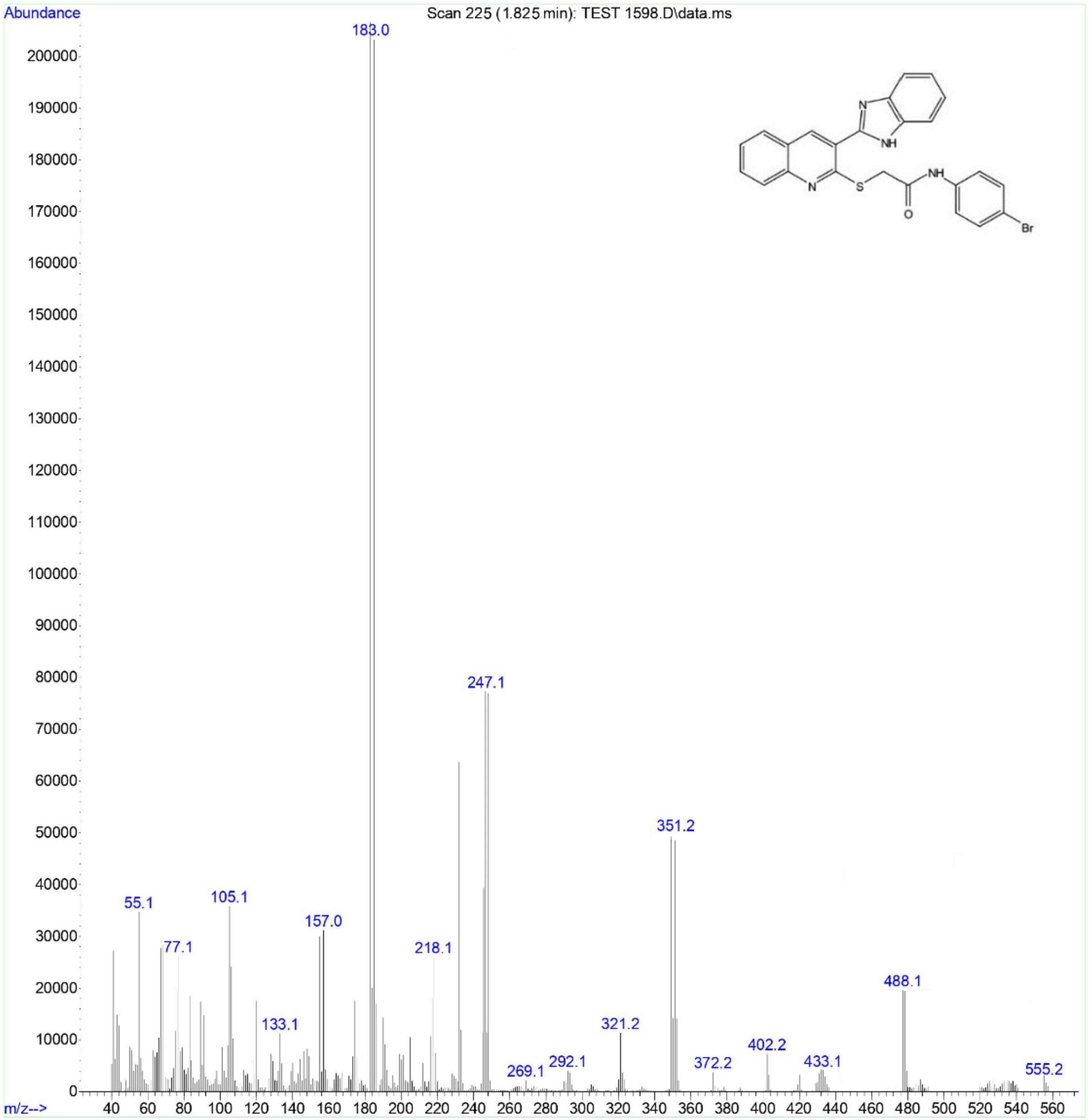

*^{1}H-NMR of 2-((3-(1H-benzo[d]imidazol-2-yl)quinolin-2-yl)thio)-N-(2,6-dichlorophenyl)acetamide (**9h**)*

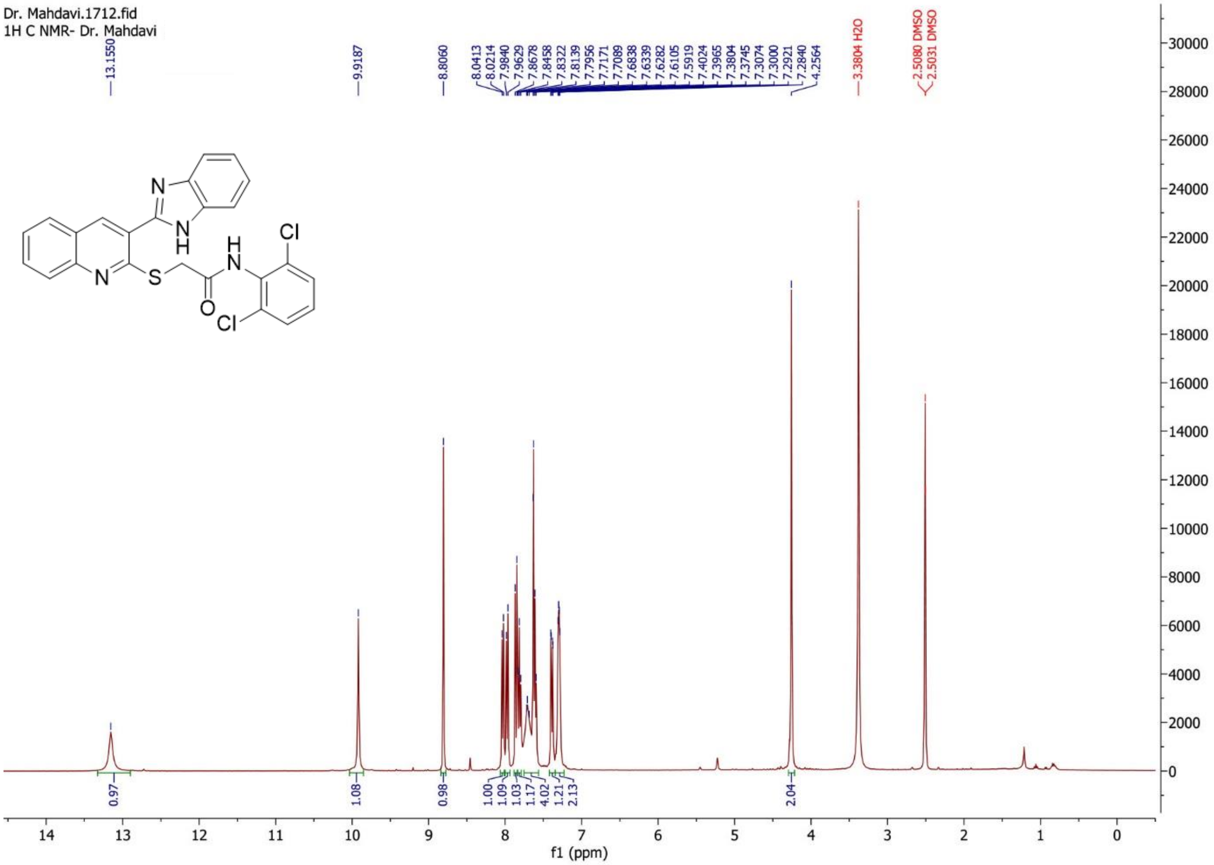

^{13}*C-NMR of 2-((3-(1H-benzo[d]imidazol-2-yl)quinolin-2-yl)thio)-N-(2,6-dichlorophenyl)acetamide (**9h**)*

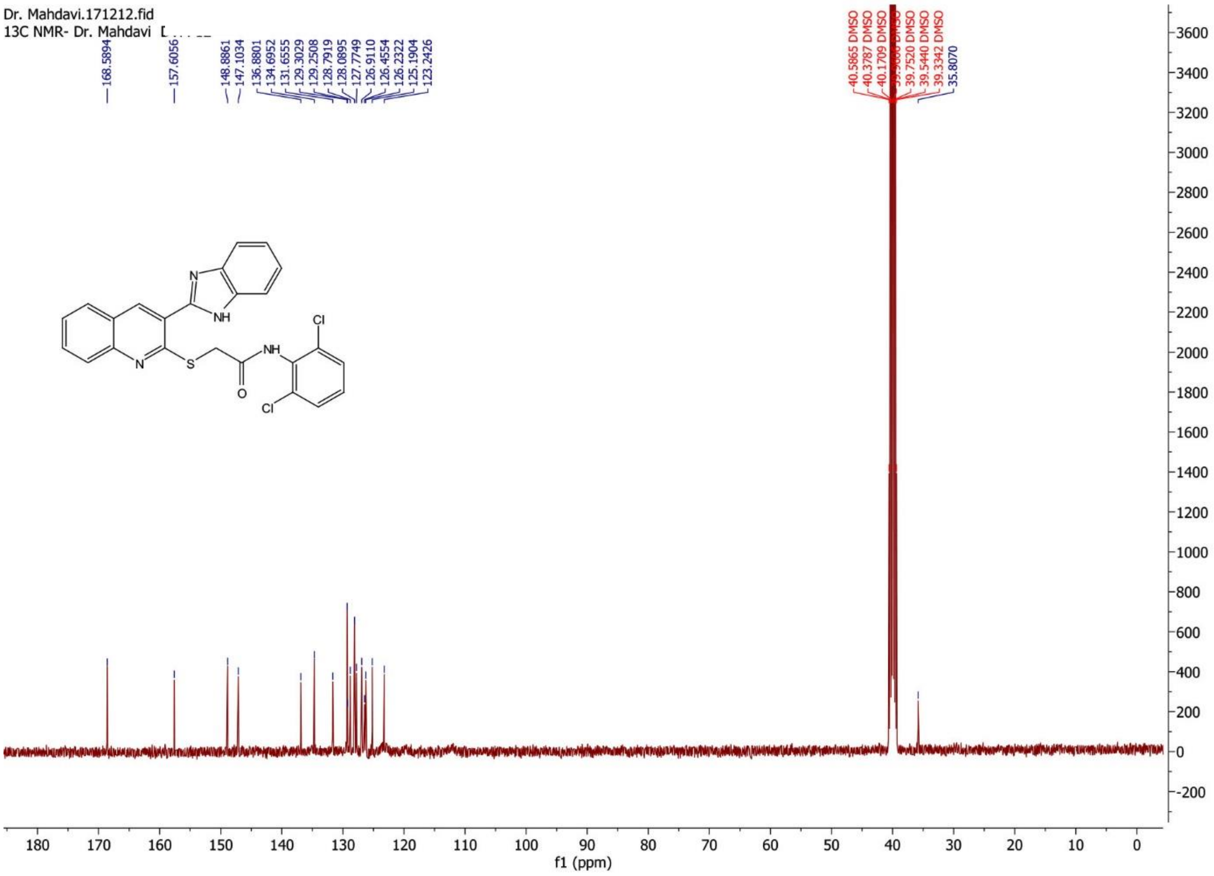

*^{13}C-NMR of 2-((3-(1H-benzo[d]imidazol-2-yl)quinolin-2-yl)thio)-N-(2,6-dichlorophenyl)acetamide (**9h**)*

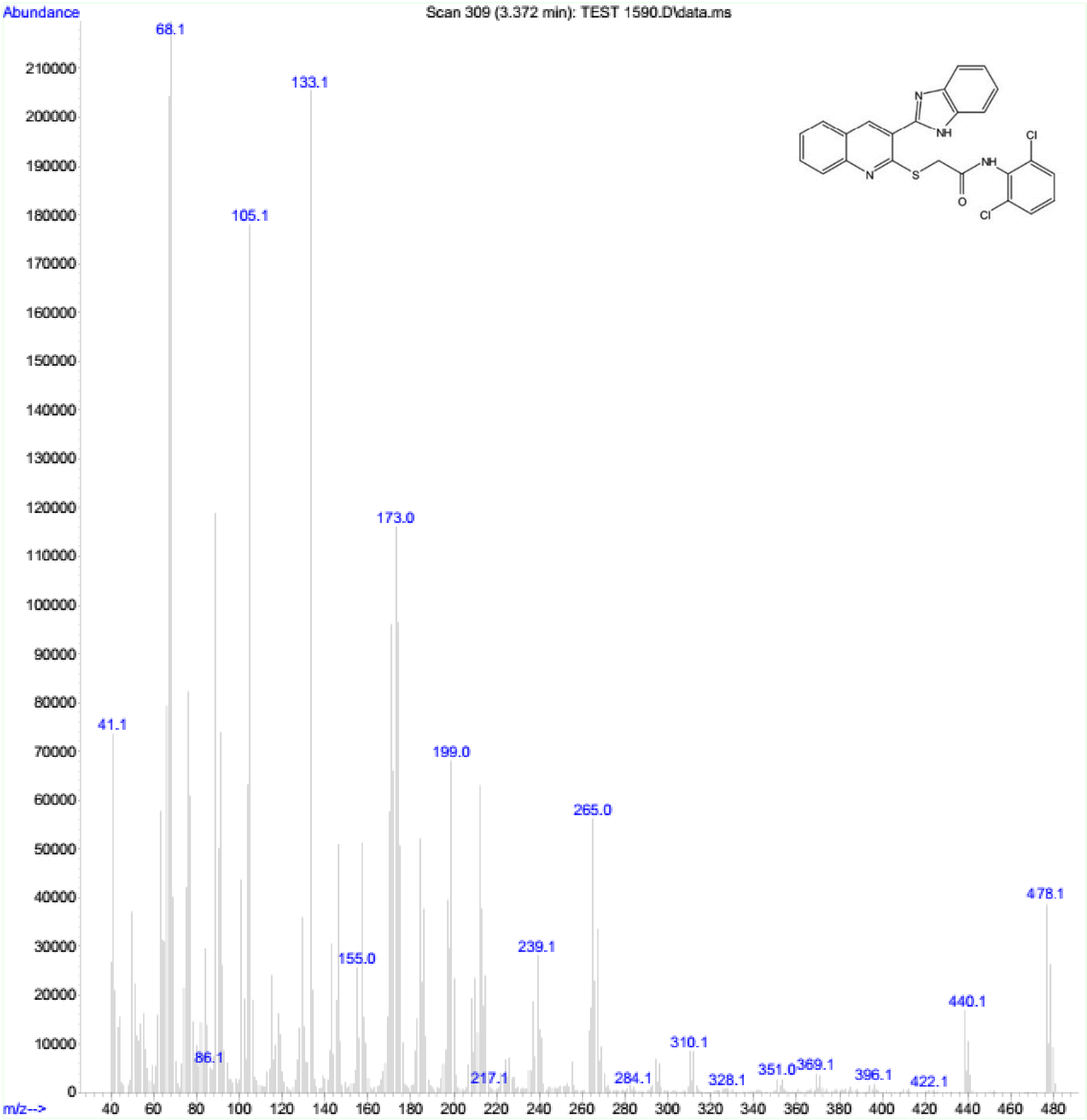

*^{1}H-NMR of 2-((3-(1H-benzo[d]imidazol-2-yl)quinolin-2-yl)thio)-N-(4-nitrophenyl)acetamide(**9i**)*

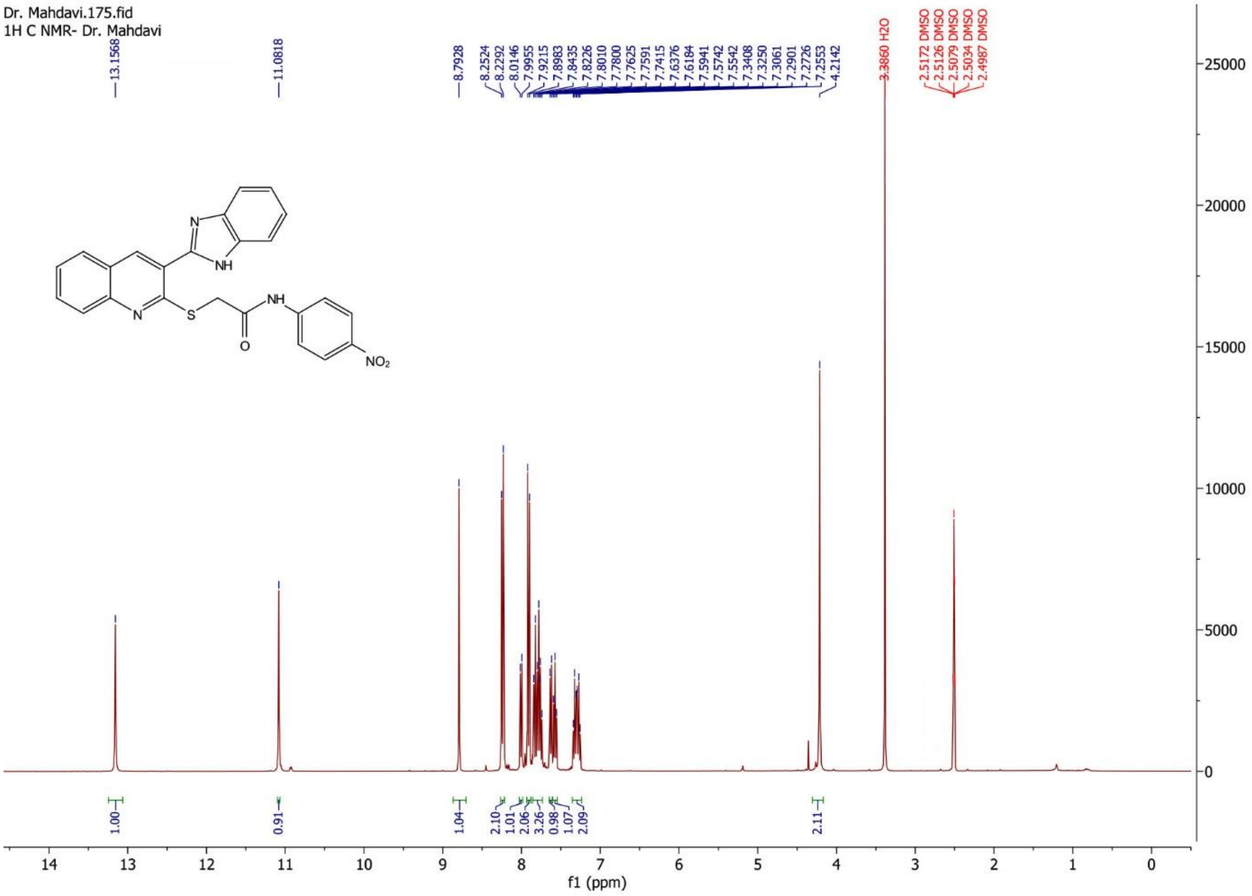

^{13}C-NMR of 2-((3-(1H-benzo[d]imidazol-2-yl)quinolin-2-yl)thio)-N-(4-nitrophenyl)acetamide(

***9i*)**

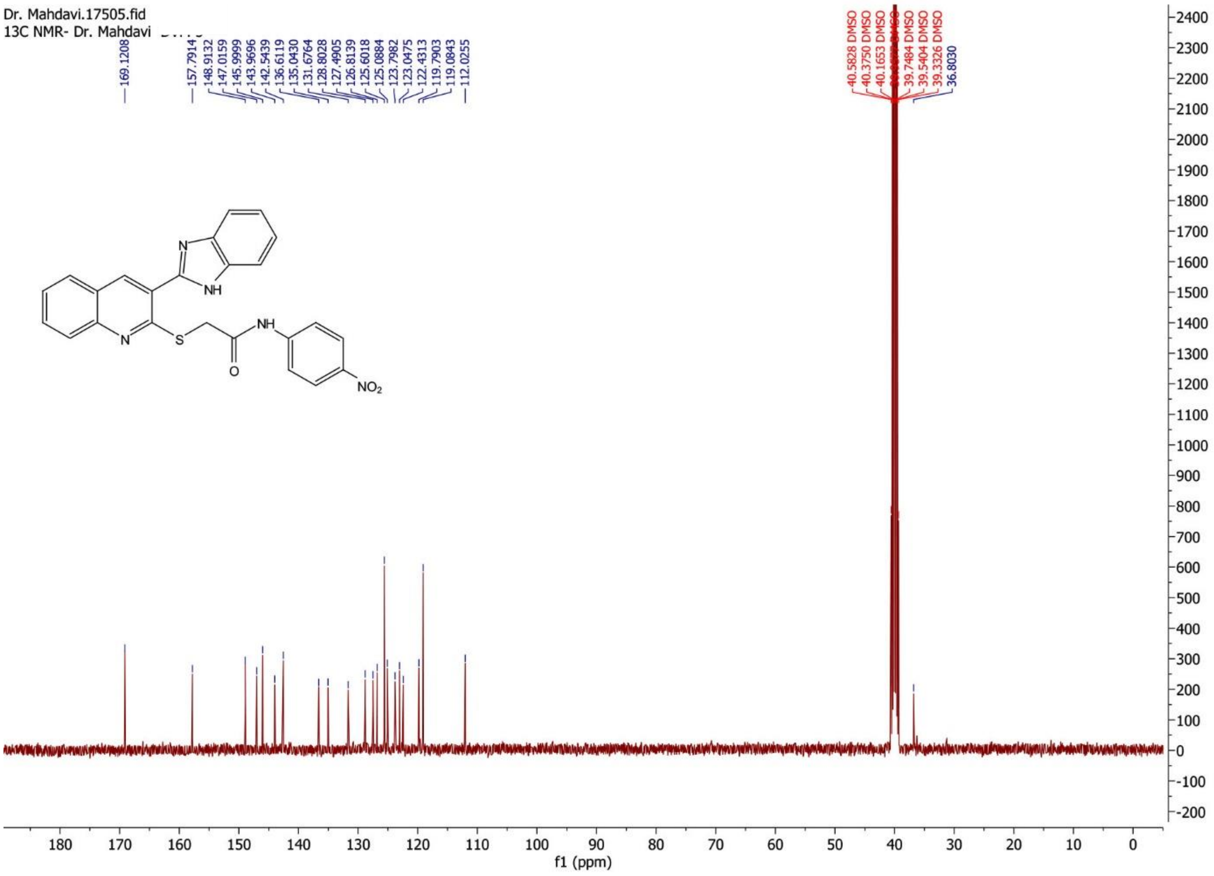

Mass of 2-((3-(1H-benzo[d]imidazol-2-yl)quinolin-2-yl)thio)-N-(4-nitrophenyl)acetamide(***9i*)**

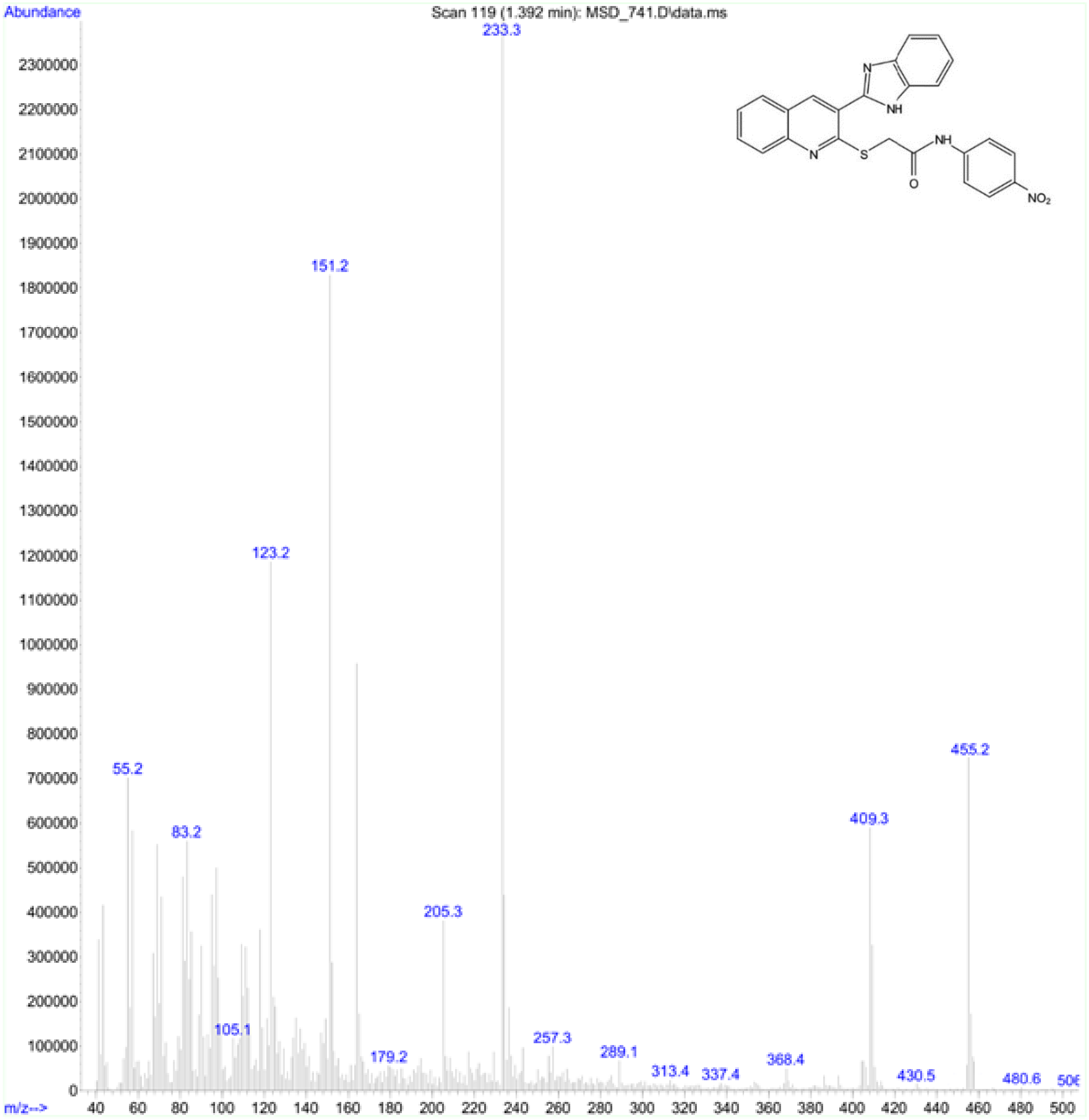

Abundance
Scan 119 (1.392 min): MSD_741.D\data.ms
233.3
151.2
123.2
55.2
83.2
105.1
179.2
205.3
257.3
289.1
313.4
337.4
368.4
409.3
430.5
455.2
480.6
m/z-->
N
NH
S
O
NH
NO2

^{1}H-NMR of 2-((3-(1H-benzo[d]imidazol-2-yl)quinolin-2-yl)thio)-N-(o-tolyl)acetamide(**9j**)

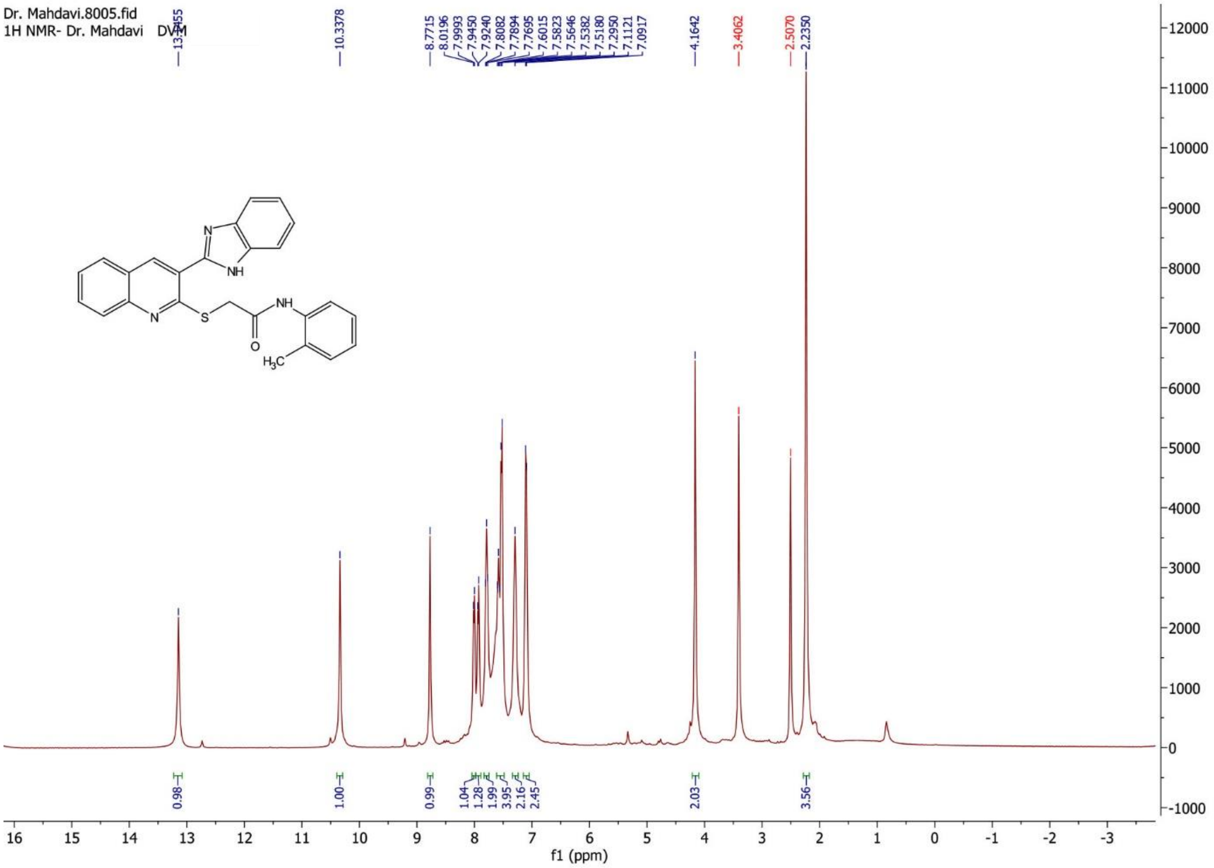

^{13}*C-NMR of 2-((3-(1H-benzo[d]imidazol-2-yl)quinolin-2-yl)thio)-N-(o-tolyl)acetamide(**9j**)*

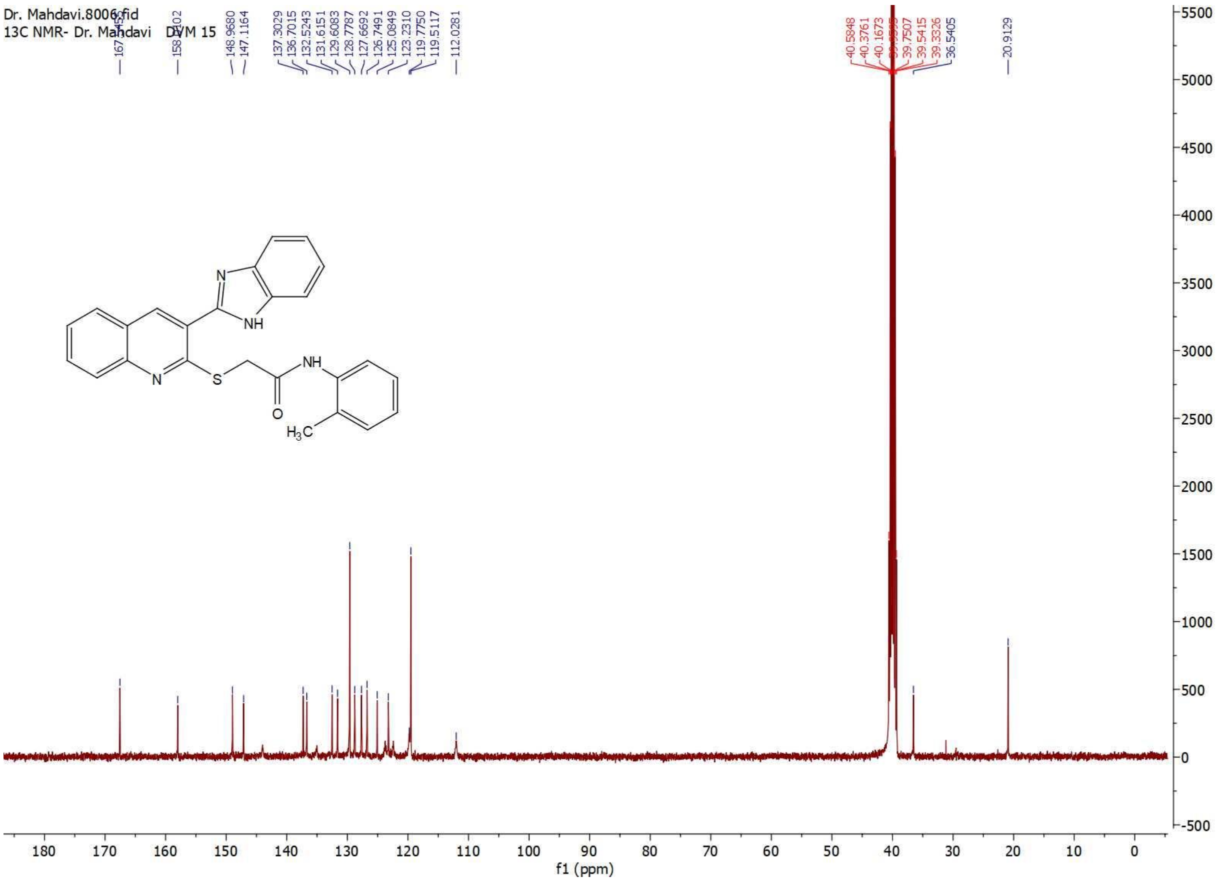

*Mass of 2-((3-(1H-benzo[d]imidazol-2-yl)quinolin-2-yl)thio)-N-(o-tolyl)acetamide(**9j**)*

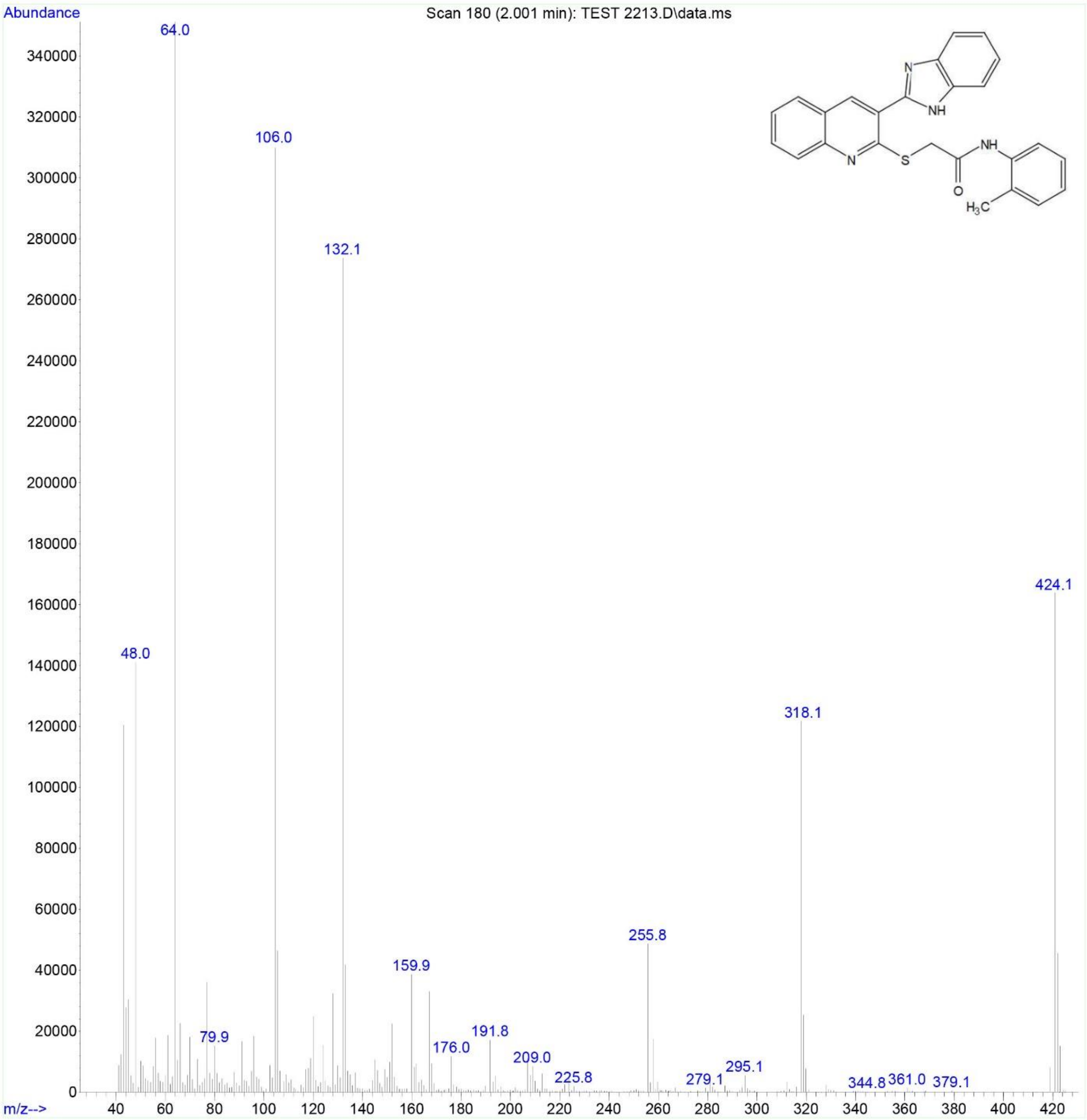

^{1}H-NMR of 2-((3-(1H-benzo[d]imidazol-2-yl)quinolin-2-yl)thio)-N-(p-tolyl)acetamide(**9k**)

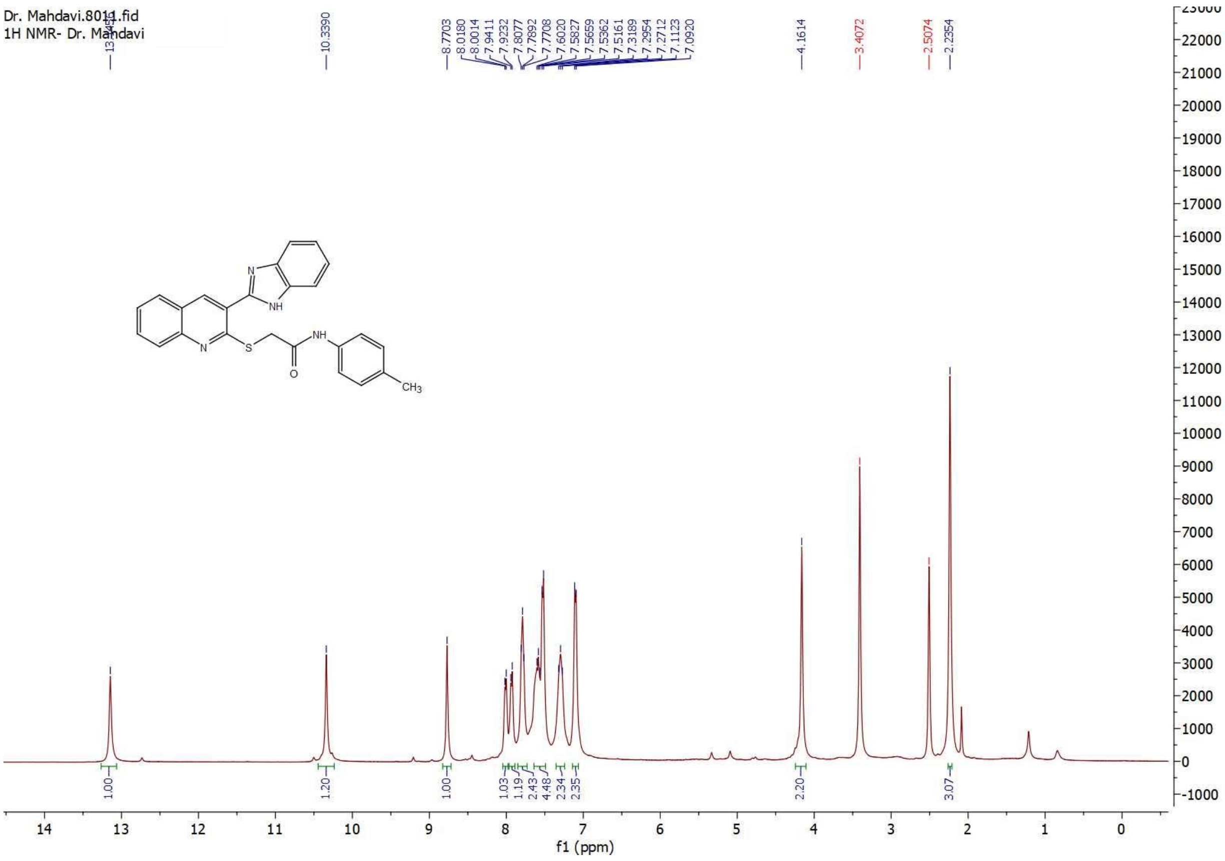

*^{13}C-NMR of 2-((3-(1H-benzo[d]imidazol-2-yl)quinolin-2-yl)thio)-N-(p-tolyl)acetamide(**9k**)*

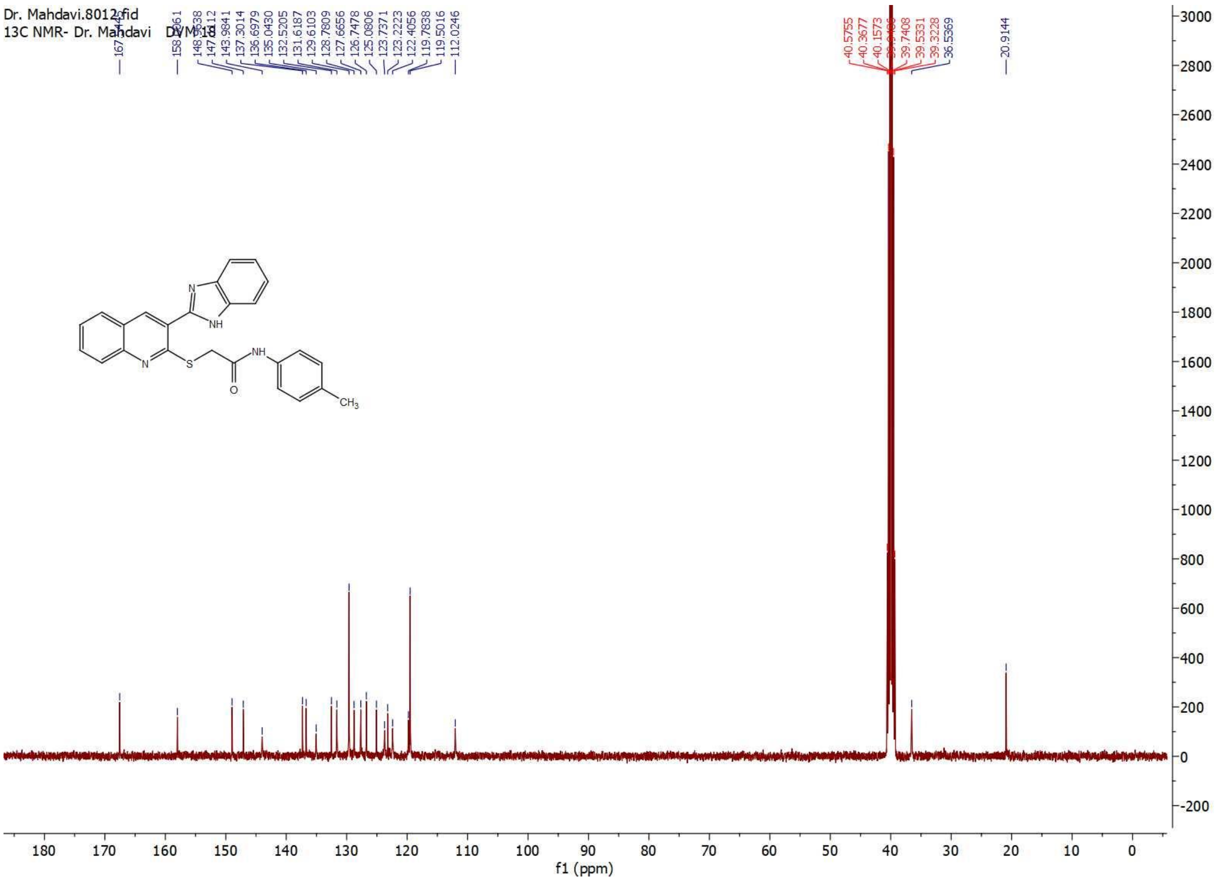

*Mass of 2-((3-(1H-benzo[d]imidazol-2-yl)quinolin-2-yl)thio)-N-(p-tolyl)acetamide(**9k**)*

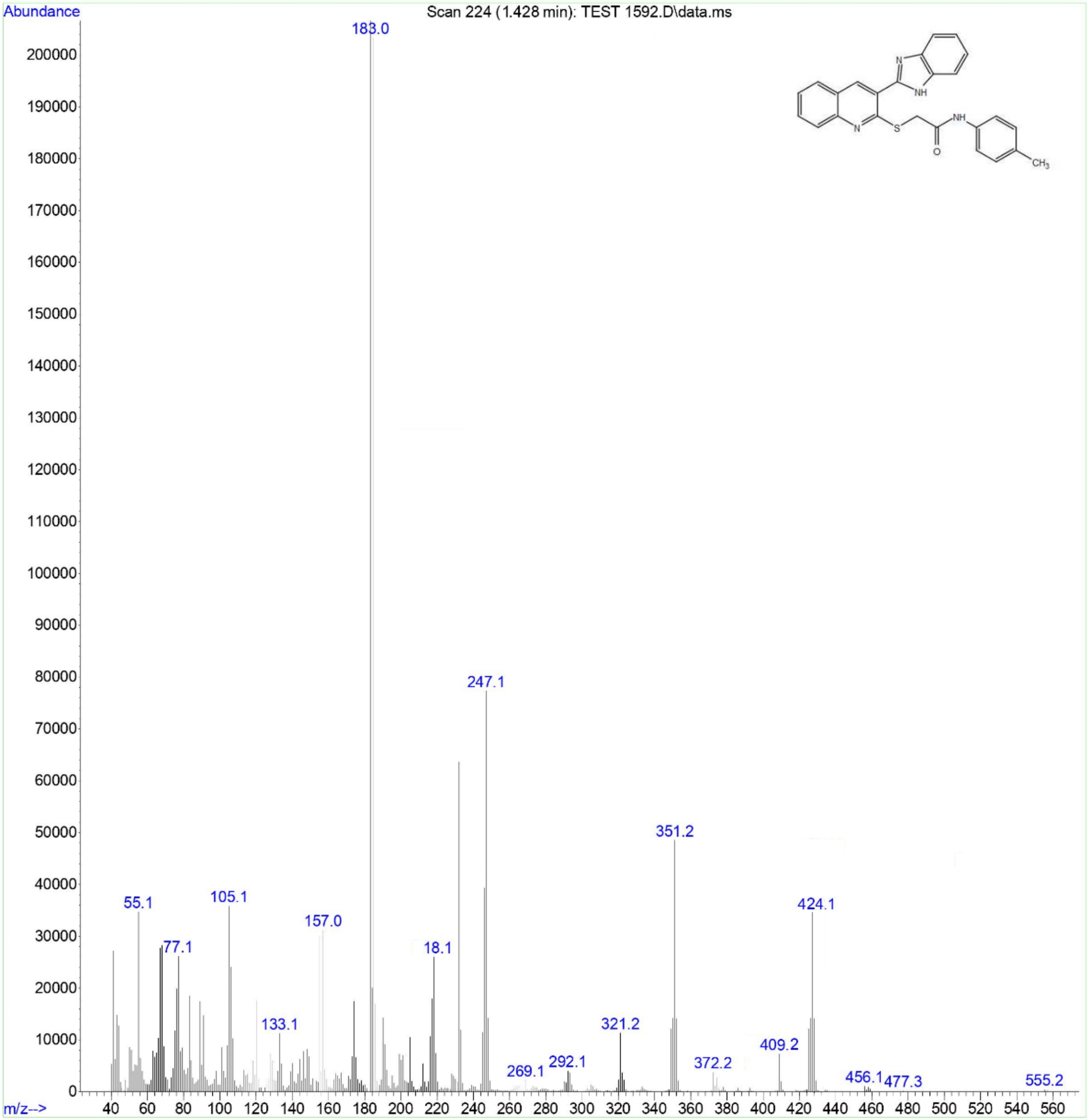

^{1}H-NMR of 2-((3-(1H-benzo[d]imidazol-2-yl)quinolin-2-yl)thio)-N-(4-methoxyphenyl)acetamide

(9l)

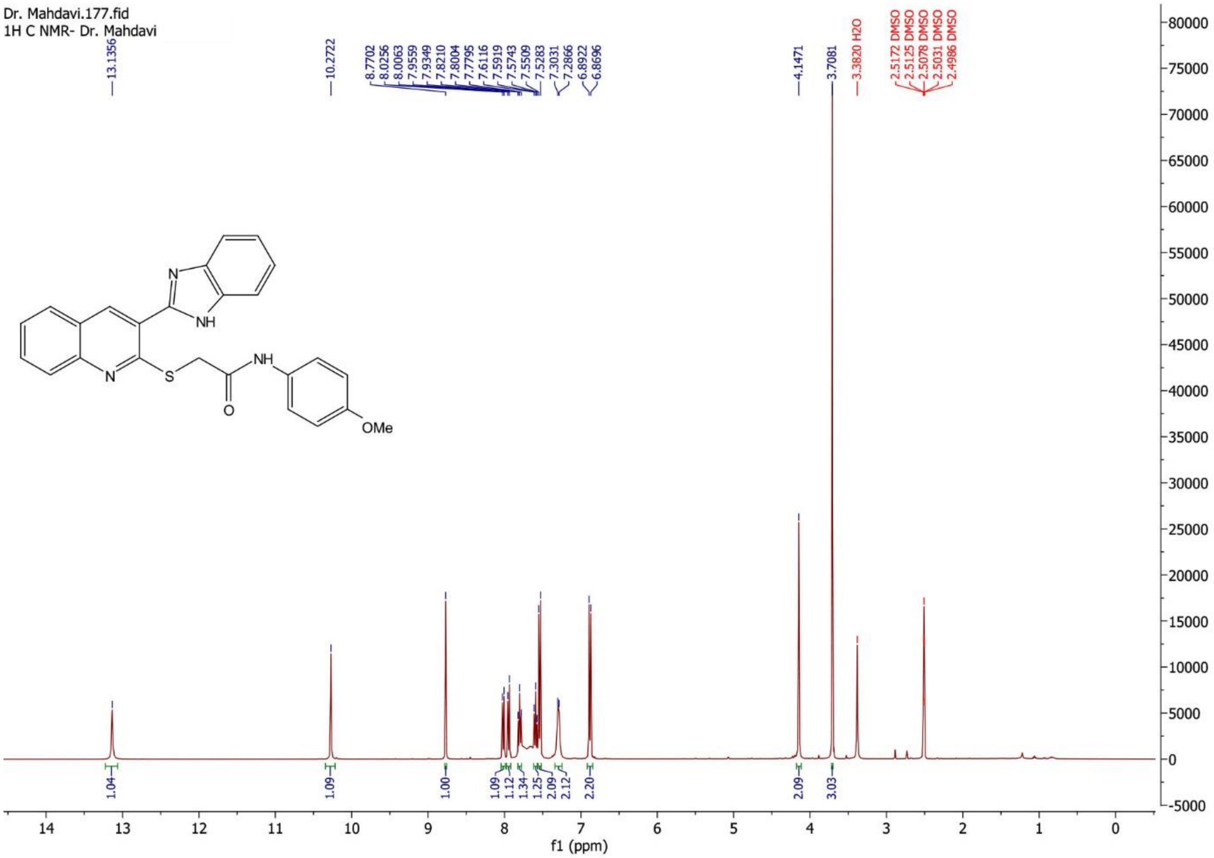

^{13}C-NMR of 2-((3-(1H-benzo[d]imidazol-2-yl)quinolin-2-yl)thio)-N-(4-methoxyphenyl)acetamide ***(9l)***

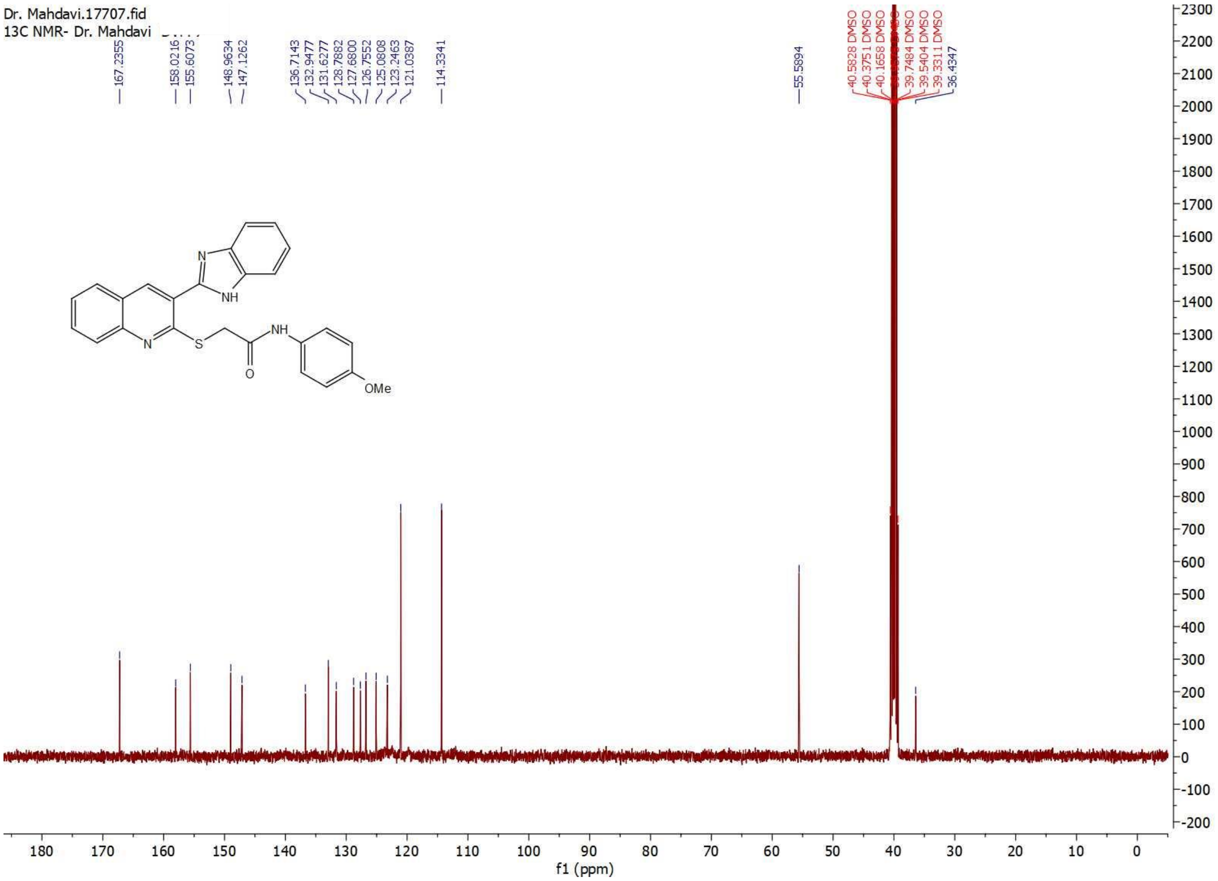

*Mass of 2-((3-(1H-benzo[d]imidazol-2-yl)quinolin-2-yl)thio)-N-(4-methoxyphenyl)acetamide (**9l**)*

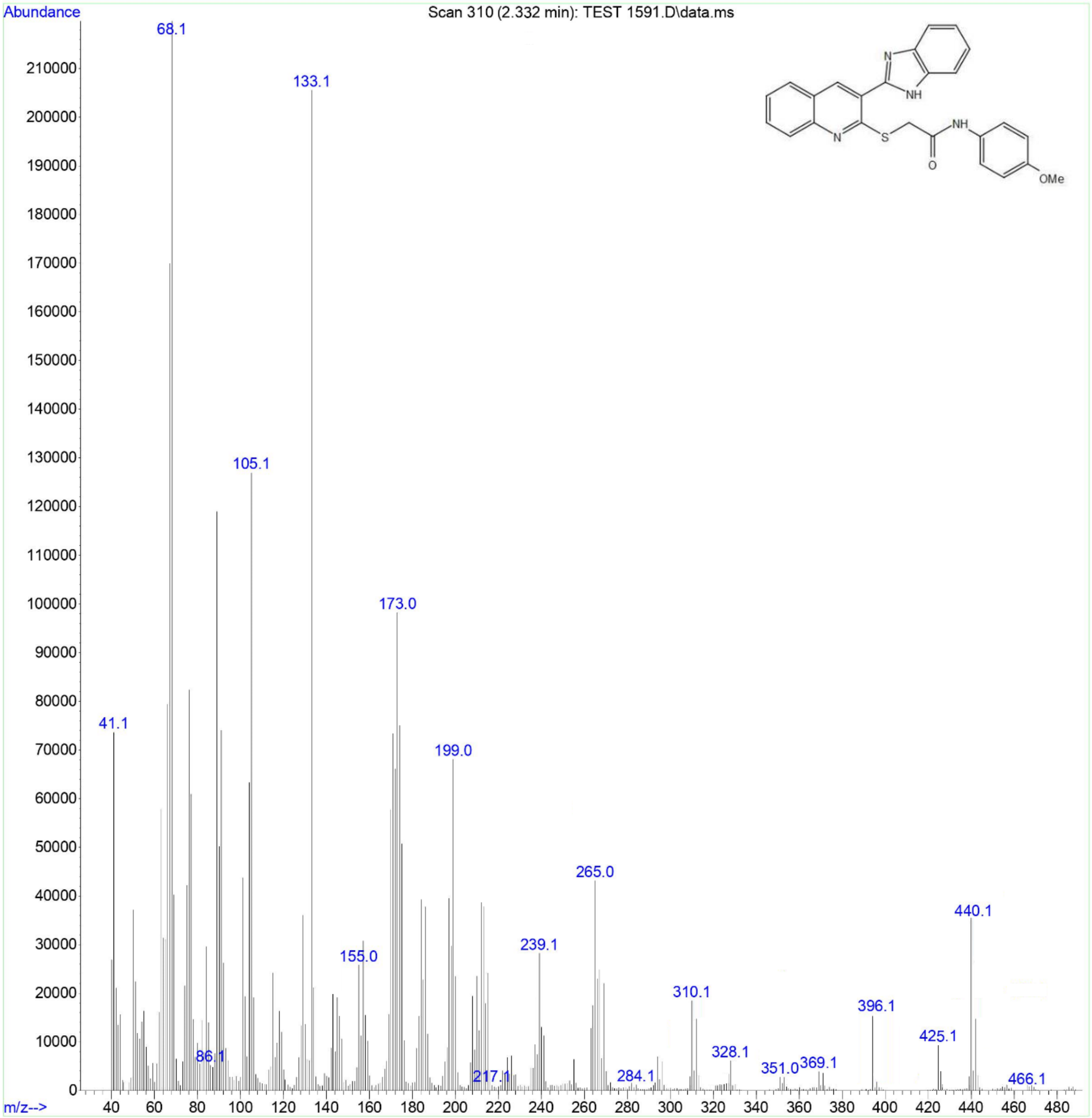

^{1}H-NMR of 2-((3-(1H-benzo[d]imidazol-2-yl)quinolin-2-yl)thio)-N-(4-ethylphenyl)acetamide (**9m**)

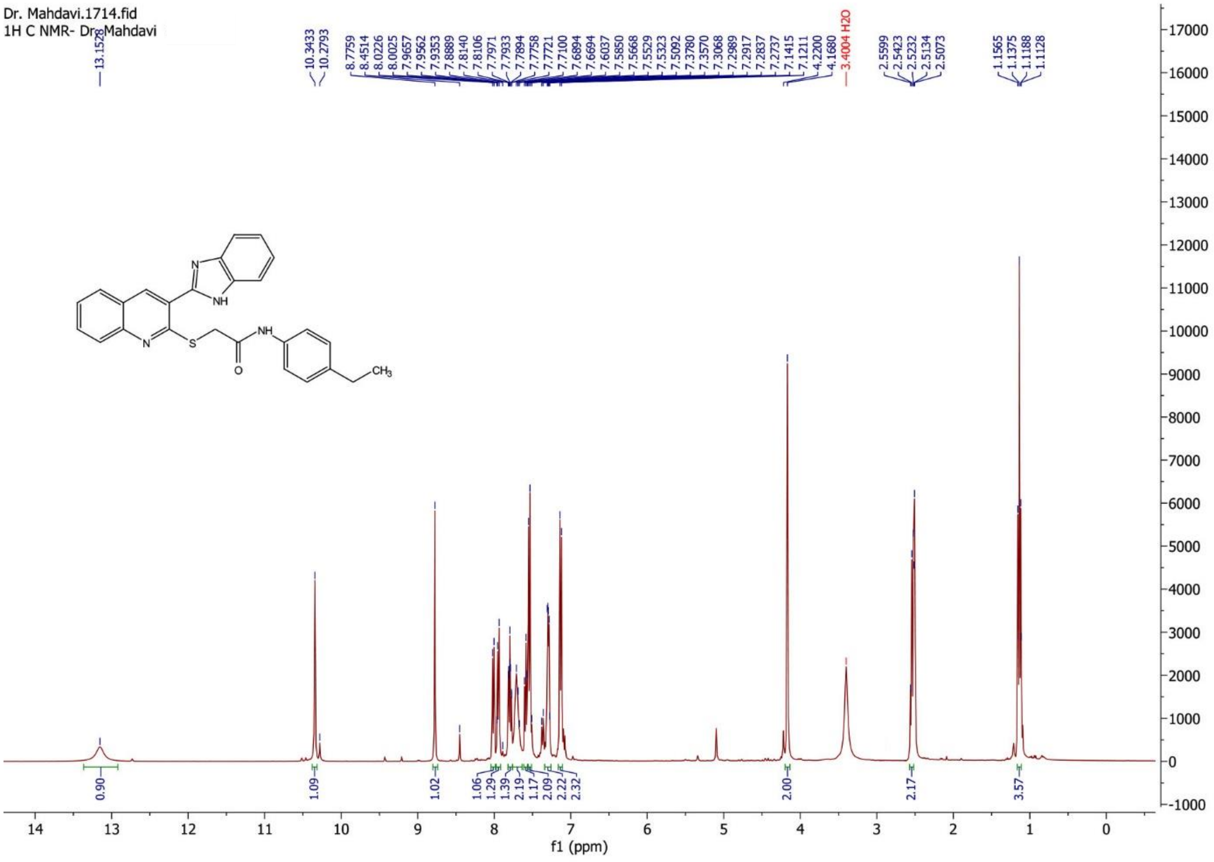

^{13}C-NMR of 2-((3-(1H-benzo[d]imidazol-2-yl)quinolin-2-yl)thio)-N-(4-ethylphenyl)acetamide

(9m)

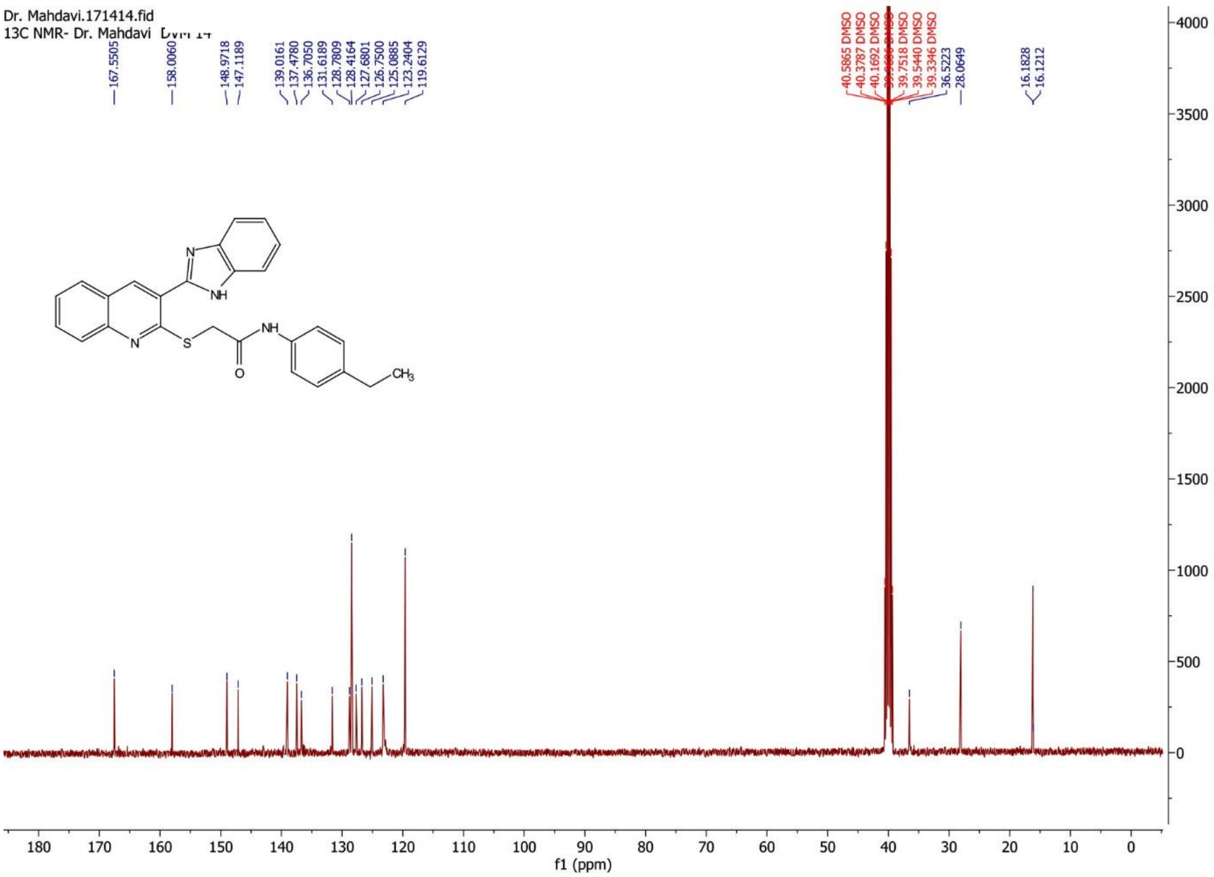

*Mass of 2-((3-(1H-benzo[d]imidazol-2-yl)quinolin-2-yl)thio)-N-(4-ethylphenyl)acetamide (**9m**)*

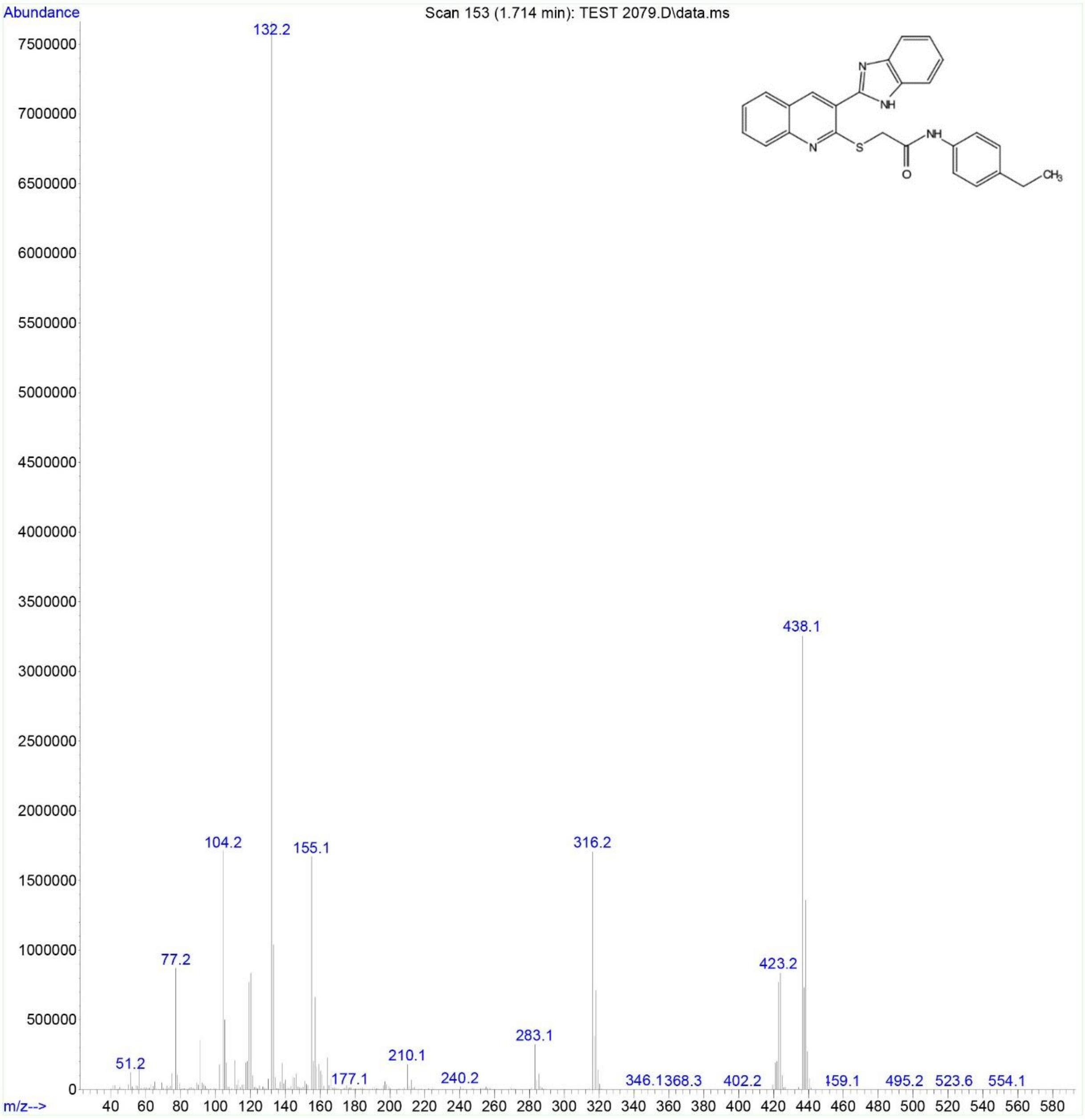

*^{1}H-NMR of 2-((3-(1H-benzo[d]imidazol-2-yl)quinolin-2-yl)thio)-N-(2,3-dimethylphenyl)acetamide (**9n**)*

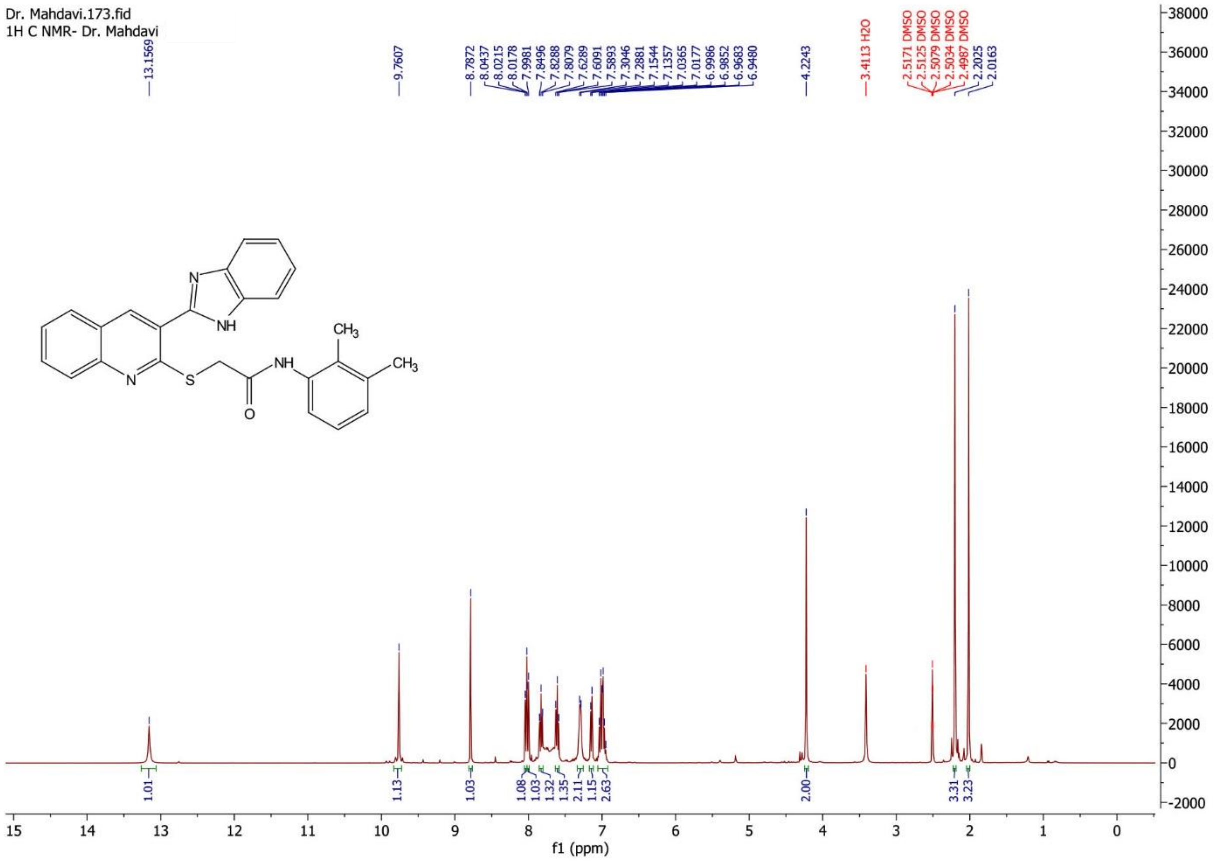

*[31]C-NMR of 2-((3-(1H-benzo[d]imidazol-2-yl)quinolin-2-yl)thio)-N-(2,3-dimethylphenyl)acetamide (**9n**)*

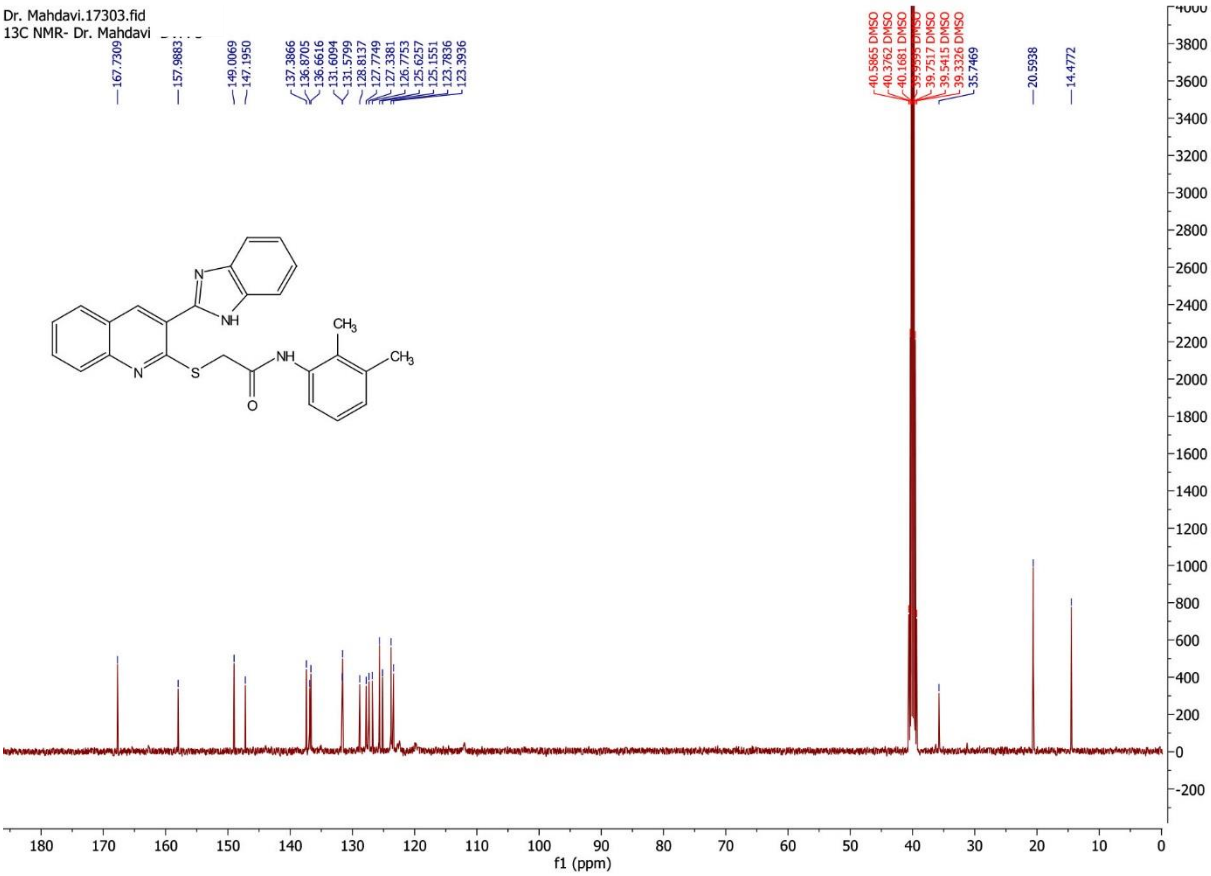

*Mass of 2-((3-(1H-benzo[d]imidazol-2-yl)quinolin-2-yl)thio)-N-(2,3-dimethylphenyl)acetamide (**9n**)*

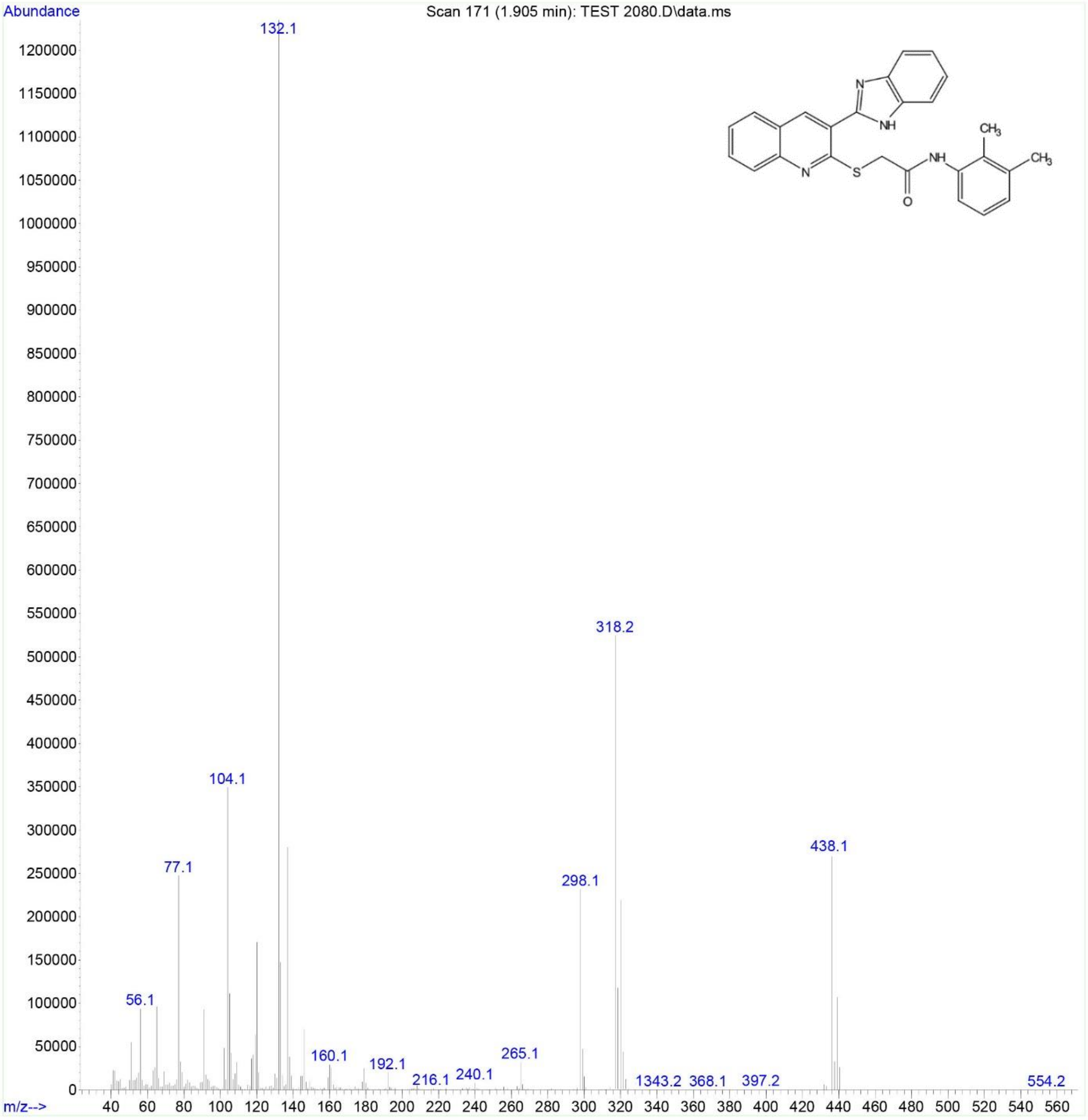

*^{1}H-NMR of 2-((3-(1H-benzo[d]imidazol-2-yl)quinolin-2-yl)thio)-N-(2,6-dimethylphenyl)acetamide (**9o**)*

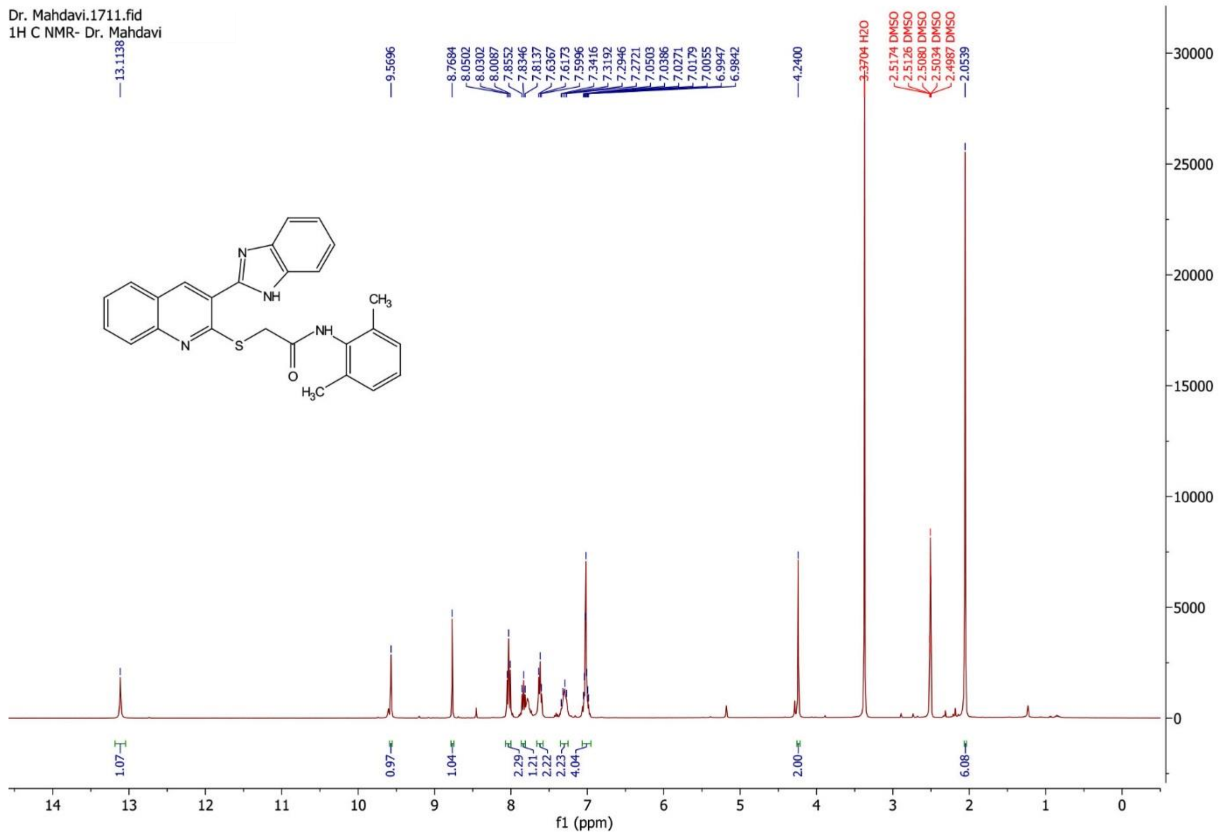

*^{13}C-NMR of 2-((3-(1H-benzo[d]imidazol-2-yl)quinolin-2-yl)thio)-N-(2,6-dimethylphenyl)acetamide (**9o**)*

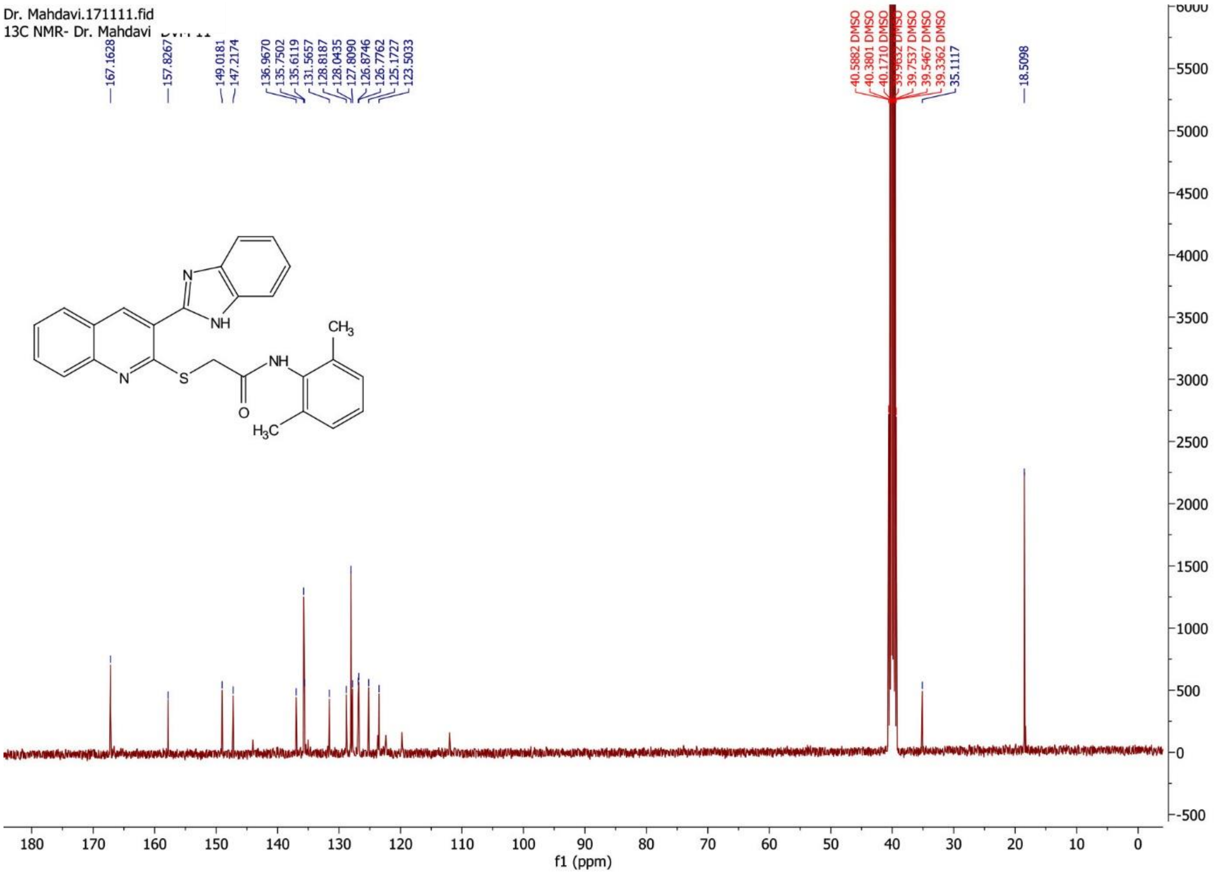

*Mass of 2-((3-(1H-benzo[d]imidazol-2-yl)quinolin-2-yl)thio)-N-(2,6-dimethylphenyl)acetamide (**9o**)*

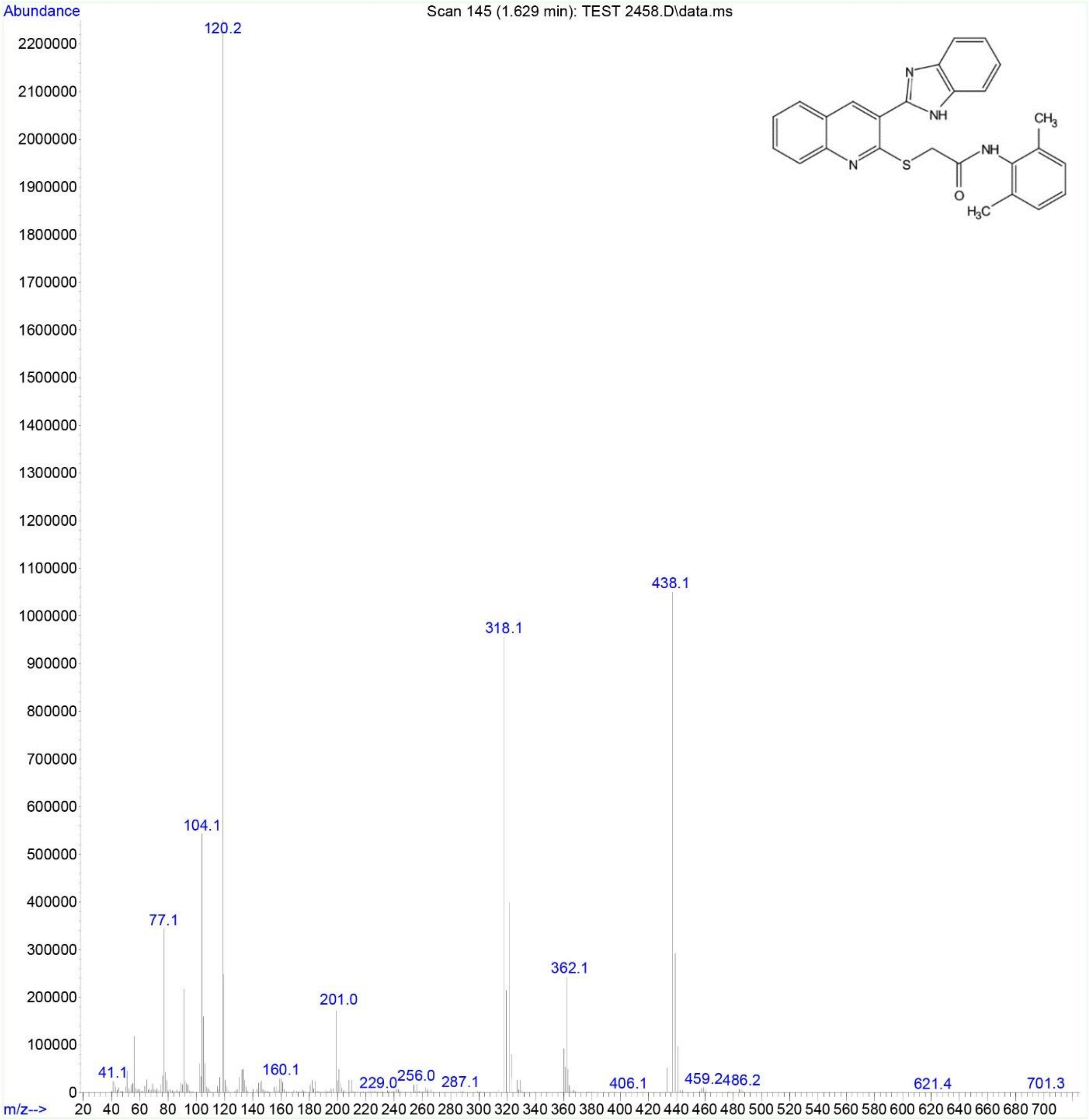

^{1}H-NMR of 2-((3-(1H-benzo[d]imidazol-2-yl)quinolin-2-yl)thio)-N-(naphthalen-2-yl)acetamide(**9p**)

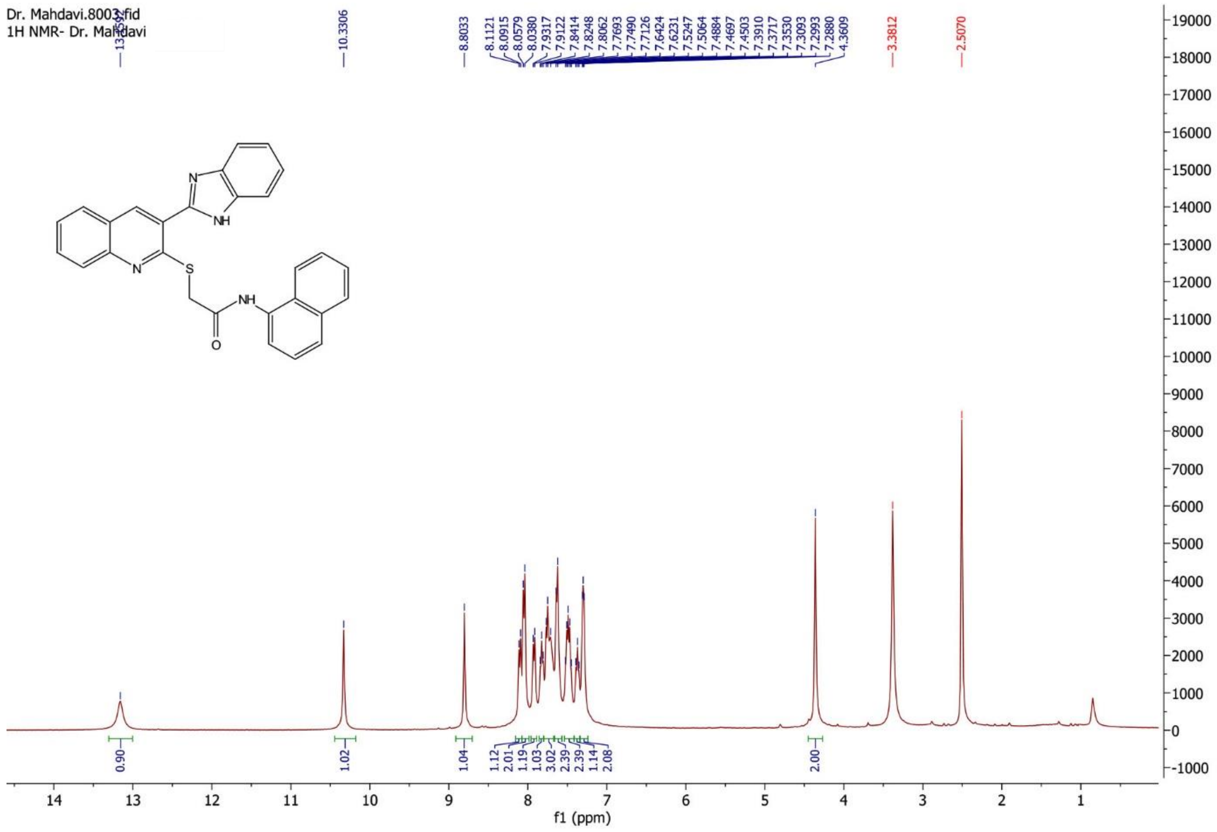

*^{13}C-NMR of 2-((3-(1H-benzo[d]imidazol-2-yl)quinolin-2-yl)thio)-N-(naphthalen-2-yl)acetamide(**9p**)*

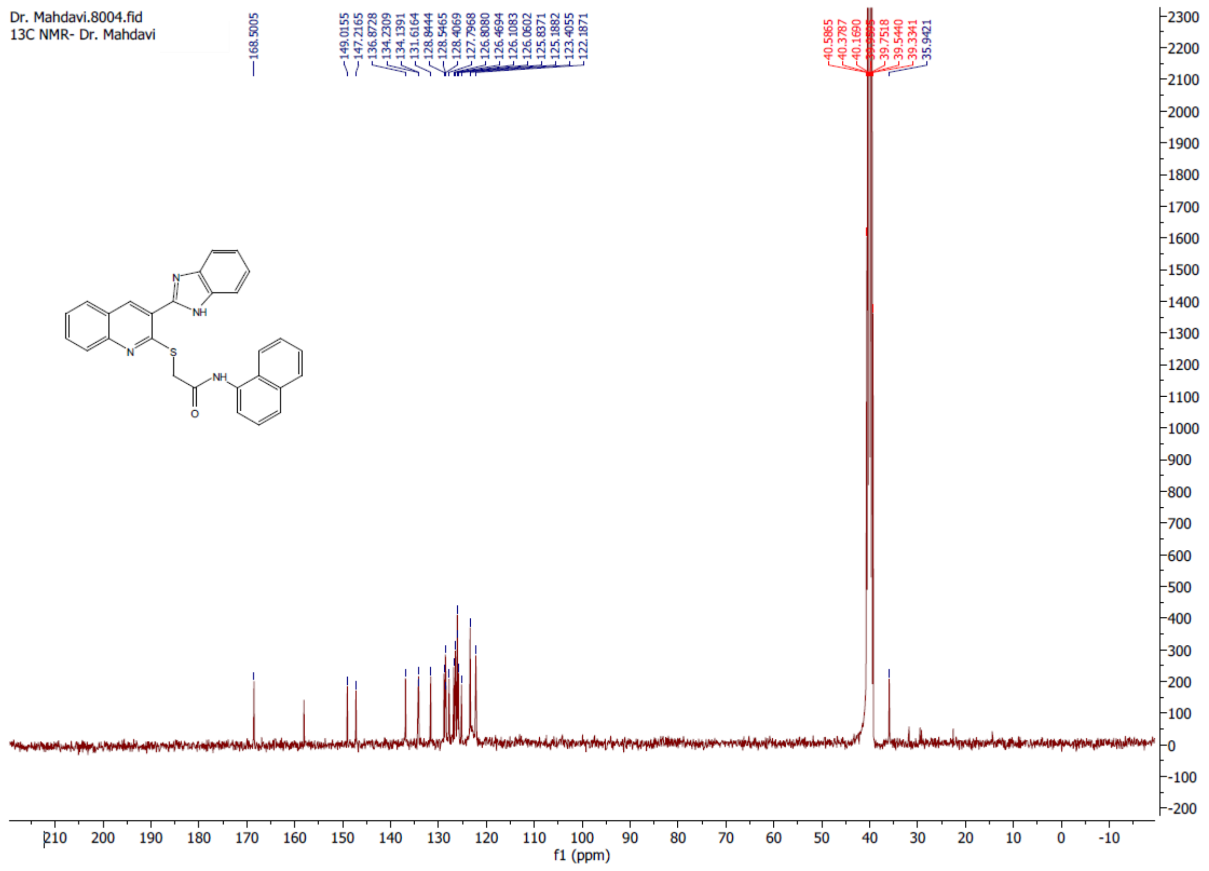

*Mass of 2-((3-(1H-benzo[d]imidazol-2-yl)quinolin-2-yl)thio)-N-(naphthalen-2-yl)acetamide(**9p**)*

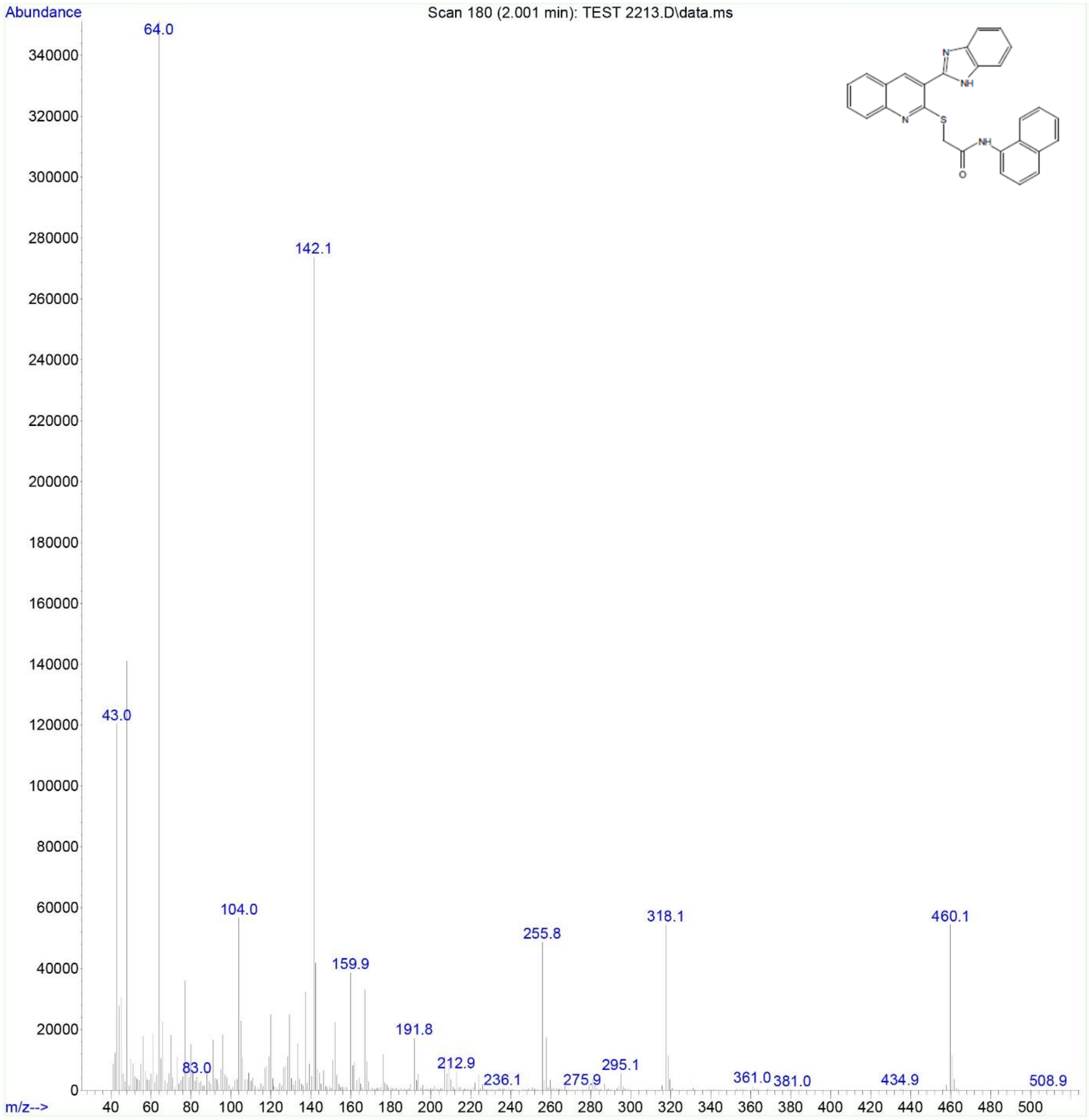

*1H-NMR of 2-((3-(1H-benzo[d]imidazol-2-yl)quinolin-2-yl)thio)-N-benzylacetamide (**9q**)*

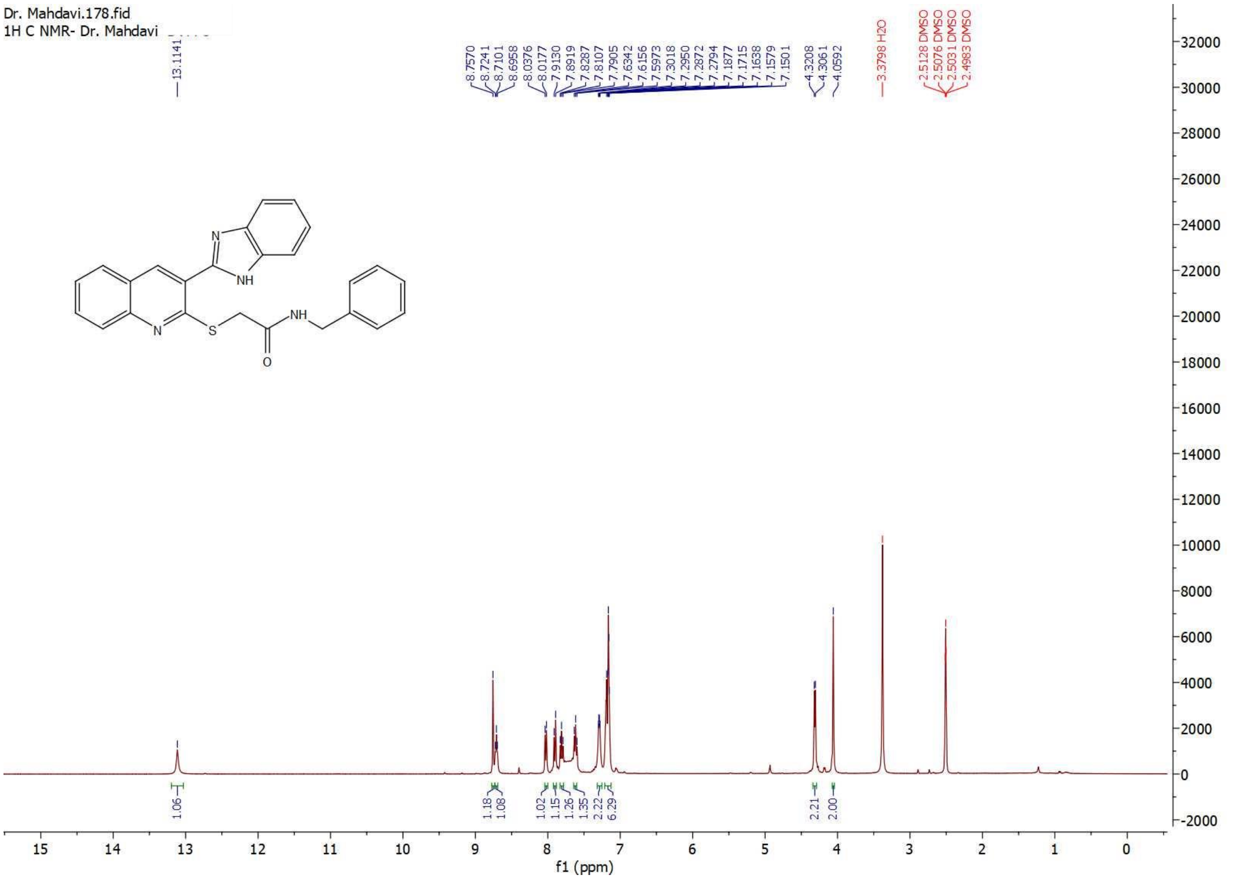

*13C-NMR of 2-((3-(1H-benzo[d]imidazol-2-yl)quinolin-2-yl)thio)-N-benzylacetamide (**9q**)*

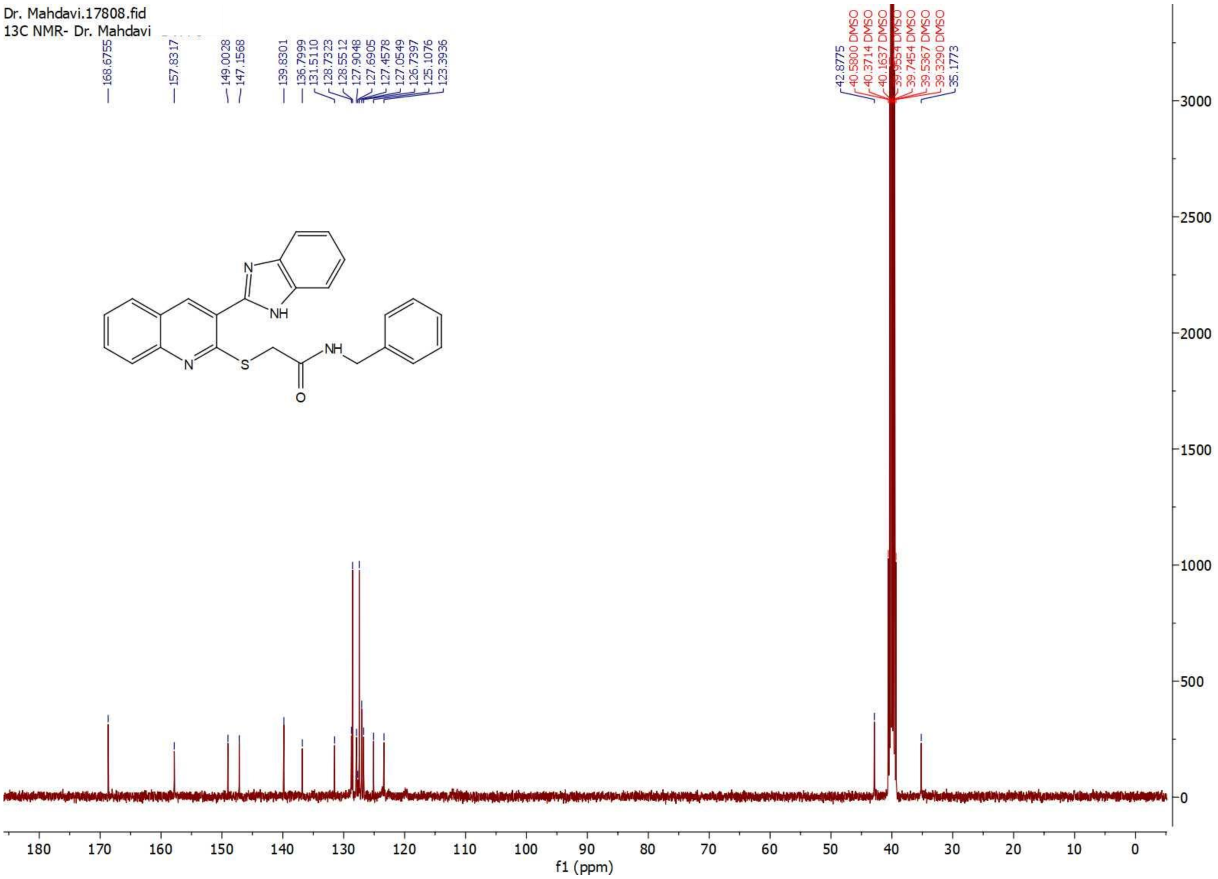

*Mass of 2-((3-(1H-benzo[d]imidazol-2-yl)quinolin-2-yl)thio)-N-benzylacetamide (**9q**)*

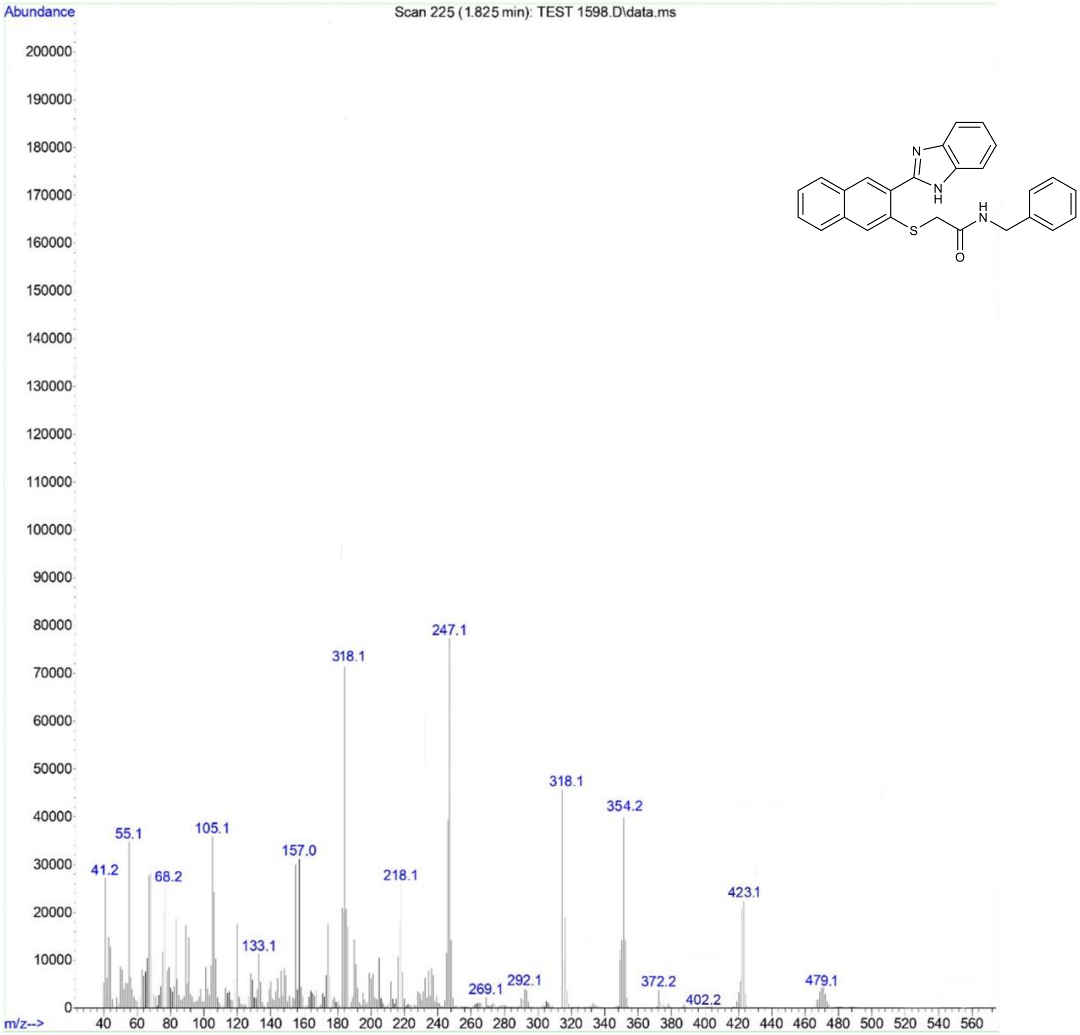

*^{1}H-NMR of 2-((3-(1H-benzo[d]imidazol-2-yl)quinolin-2-yl)thio)-N-(4-fluorobenzyl)acetamide(**9r**)*

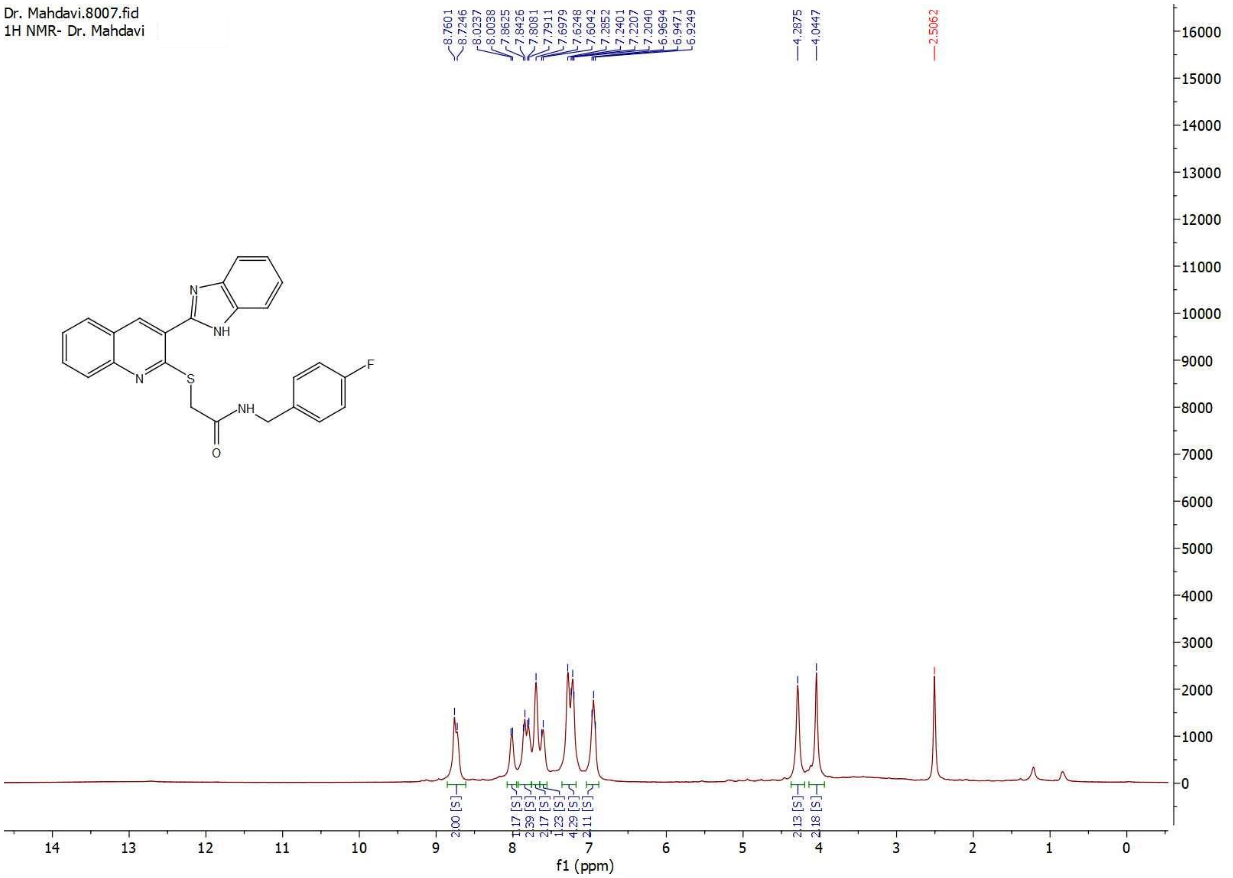

*^{13}C-NMR of 2-((3-(1H-benzo[d]imidazol-2-yl)quinolin-2-yl)thio)-N-(4-fluorobenzyl)acetamide(**9r**)*

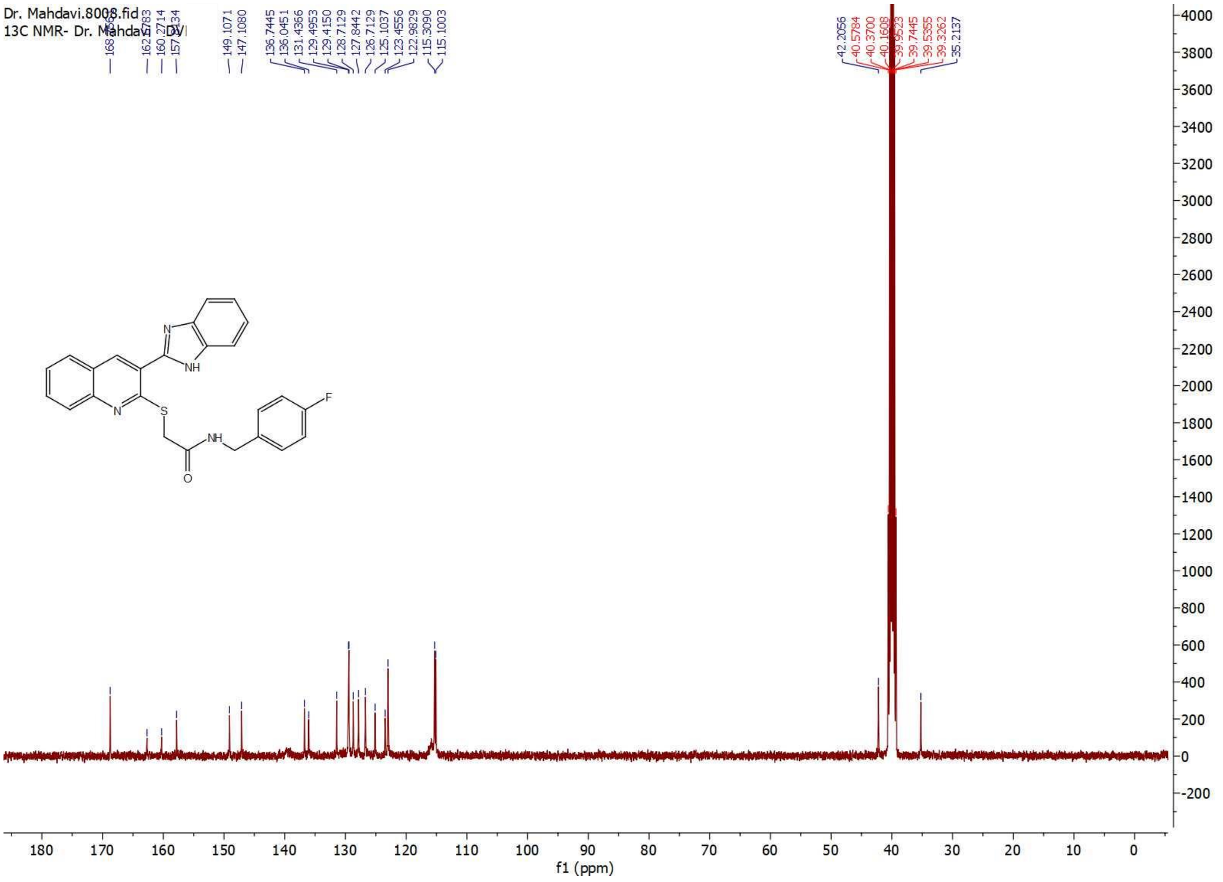

*Mass of 2-((3-(1H-benzo[d]imidazol-2-yl)quinolin-2-yl)thio)-N-(4-fluorobenzyl)acetamide(**9r**)*

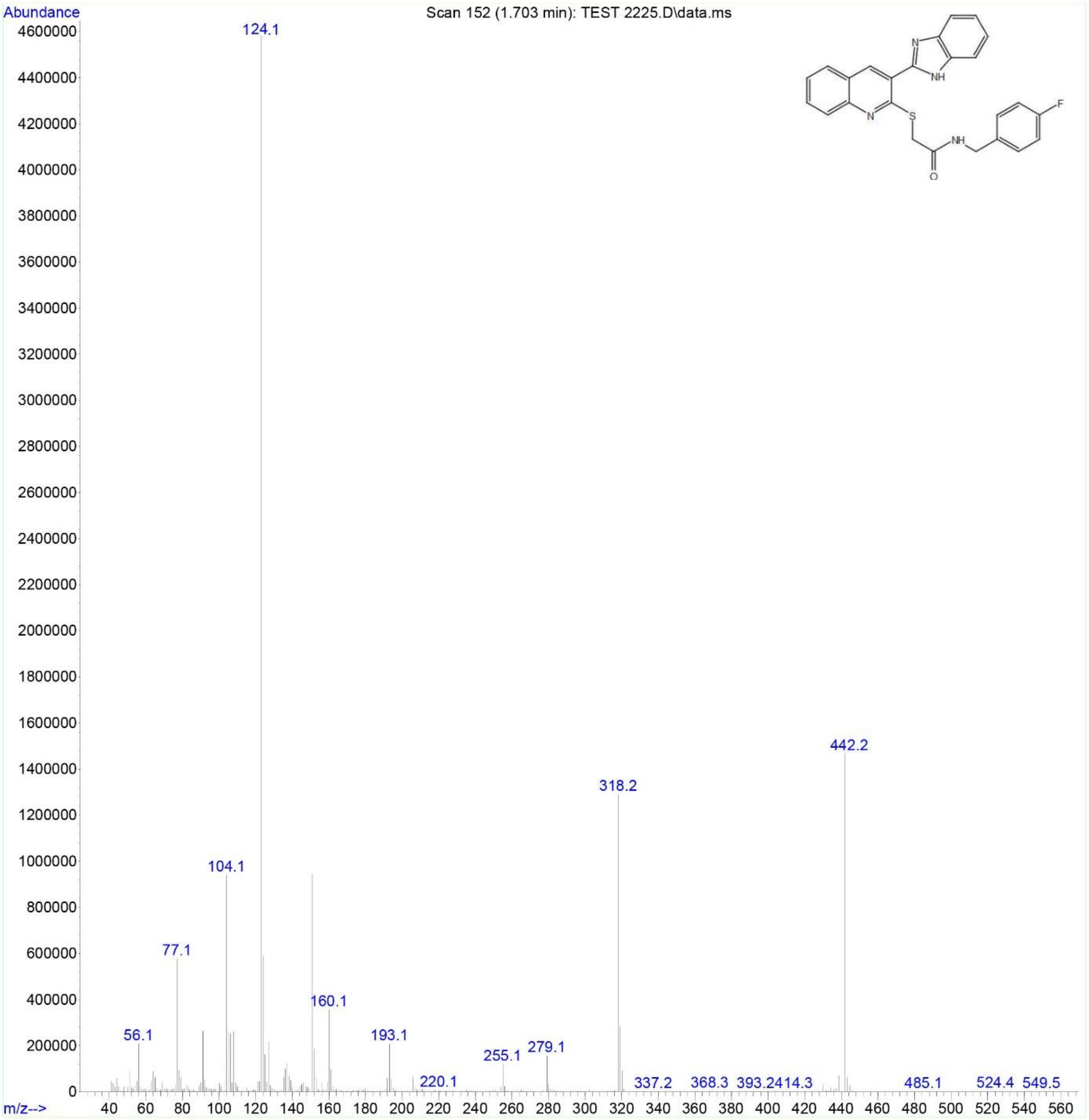

Supplement: Supplementary file 1 — Supplementary Information. [file 41598_2022_18455_MOESM1_ESM.pdf]
